# Trapping the substrate radical of heme synthase AhbD

**DOI:** 10.3389/fchem.2024.1430796

**Published:** 2024-07-25

**Authors:** Lorenz Heidinger, Isabelle Fix, Thorsten Friedrich, Gunhild Layer

**Affiliations:** ^1^ Institut für Biochemie, Fakultät für Chemie und Pharmazie, Albert-Ludwigs-Universität Freiburg, Freiburg, Germany; ^2^ Institut für Pharmazeutische Wissenschaften, Fakultät für Chemie und Pharmazie, Albert-Ludwigs-Universität Freiburg, Freiburg, Germany

**Keywords:** siroheme-dependent heme biosynthesis, radical SAM enzymes, iron-coproporphyrin III, EPR spectroscopy, electron transfer

## Abstract

The heme synthase AhbD catalyzes the last step of the siroheme-dependent heme biosynthesis pathway, which is operative in archaea and sulfate-reducing bacteria. The AhbD-catalyzed reaction consists of the oxidative decarboxylation of two propionate side chains of iron-coproporphyrin III to the corresponding vinyl groups of heme *b*. AhbD is a Radical SAM enzyme employing radical chemistry to achieve the decarboxylation reaction. Previously, it was proposed that the central iron ion of the substrate iron-coproporphyrin III participates in the reaction by enabling electron transfer from the initially formed substrate radical to an iron-sulfur cluster in AhbD. In this study, we investigated the substrate radical that is formed during AhbD catalysis. While the iron-coproporphyrinyl radical was not detected by electron paramagnetic resonance (EPR) spectroscopy, trapping and visualization of the substrate radical was successful by employing substrate analogs such as coproporphyrin III and zinc-coproporphyrin III. The radical signals detected by EPR were analyzed by simulations based on density functional theory (DFT) calculations. The observed radical species on the substrate analogs indicate that hydrogen atom abstraction takes place at the β-position of the propionate side chain and that an electron donating ligand is located in proximity to the central metal ion of the porphyrin.

## 1 Introduction

**FIGURE 1:**
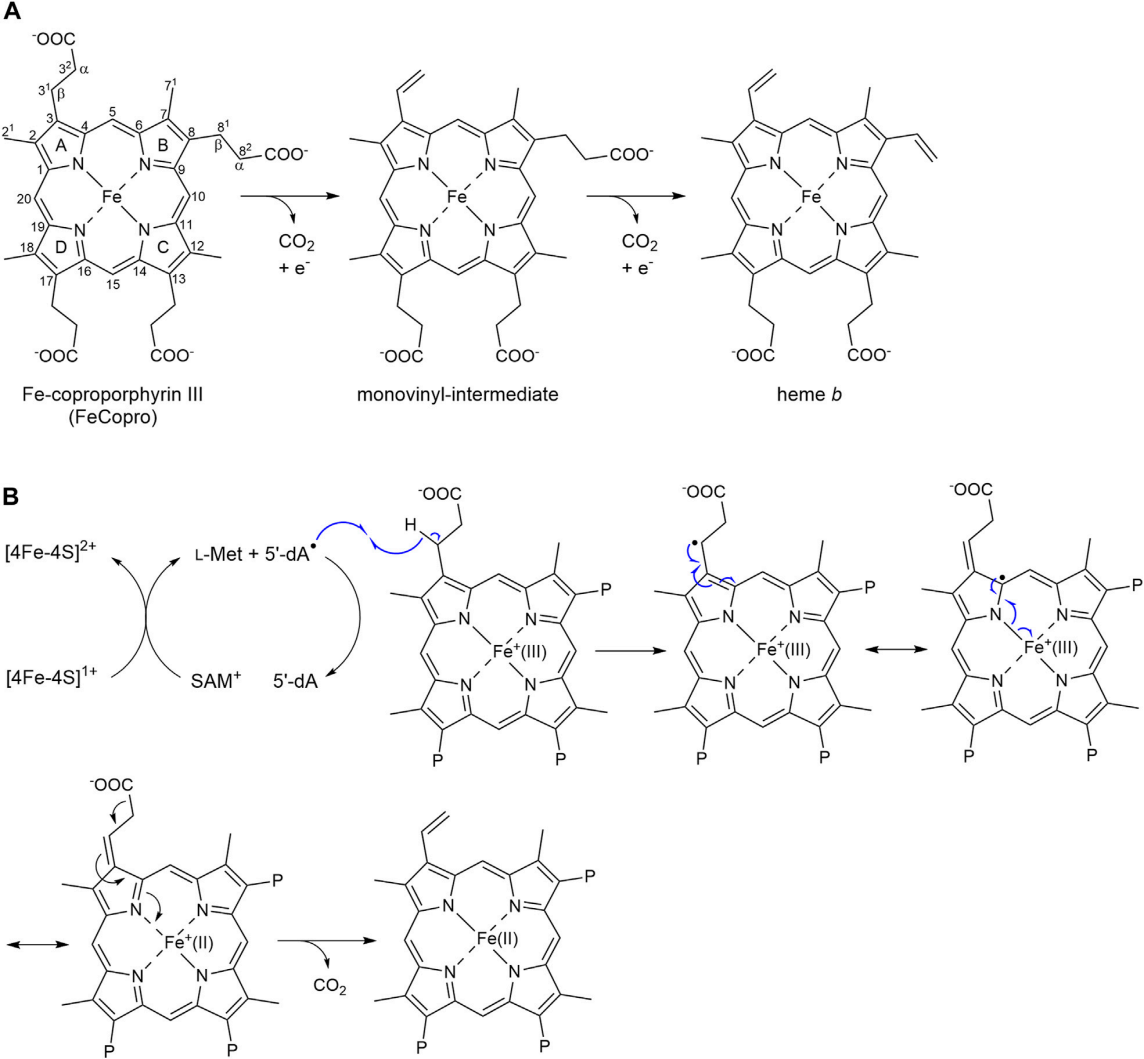
Reaction catalyzed by AhbD. **(A)** AhbD catalyzes the oxidative decarboxylation of two propionate side chains of iron-coproporphyrin III (FeCopro) to the corresponding vinyl groups of heme *b*. The decarboxylation reactions take place stepwise via a monovinyl-intermediate. The order of the decarboxylation reactions (first at C3 or C8) is not known. Numbering of the carbon atoms is according to IUPAC and pyrrole rings are denoted with A-D. The α- and β-positions of the propionate side chains are indicated. **(B)** Potential reaction mechanism for substrate radical formation by hydrogen atom abstraction at C3^1^ (or C8^1^) and radical quenching by reduction of the central iron ion from Fe(III) to Fe(II) (Kühner et al., 2016). Only some of the mesomeric structures of the delocalized substrate radical are shown. P = propionate.

Heme plays an important role as prosthetic group in proteins involved in gas transport and sensing, electron transfer as well as catalysis (Paoli et al., 2002). Depending on the organism, heme *b* is synthesized by either one of three different pathways (Dailey et al., 2017). In archaea and sulfate-reducing bacteria, the siroheme-dependent (SHD) heme biosynthesis pathway is found, which consists of three enzymatic steps converting siroheme into heme *b* (Bali et al., 2011). In the first step, siroheme decarboxylase AhbA/B catalyzes the decarboxylation of two acetate side chains to methyl groups (Palmer et al., 2014). Then, two acetate side chains are completely removed by the enzyme AhbC yielding iron-coproporphyrin III (FeCopro). In the last step of the SHD route, heme synthase AhbD is responsible for the oxidative decarboxylation of two propionate side chains of FeCopro to the corresponding vinyl groups of heme *b* (Bali et al., 2011). The overall reaction proceeds stepwise via a monovinyl-intermediate (Figure 1A) (Lobo et al., 2014; Kühner et al., 2016), however, the actual sequence of the two decarboxylation reactions is not known.

AhbD belongs to the family of Radical *S*-adenosyl-l-methionine (SAM) enzymes (Sofia et al., 2001). As such, AhbD carries a [4Fe-4S] cluster, which is coordinated by three conserved cysteine residues and one molecule of SAM constituting the fourth cluster ligand. This cofactor arrangement is common to all Radical SAM enzymes and is used to initiate radical catalysis (Frey et al., 2008). In the first step, the Radical SAM [4Fe-4S]^2+^ cluster is reduced to the 1+ state. The electron is further transferred to the cluster-bound SAM, which is cleaved to methionine and a cluster-bound 5′-deoxyadenosyl radical (5′-dA^•^) termed intermediate Ω (Broderick et al., 2018). Then, the 5′-dA^•^ liberated from Ω abstracts a hydrogen atom from the respective substrate yielding 5′-deoxyadenosine (5′-dA) and a substrate radical. The following reactions converting the substrate radical into the final product are different for each individual Radical SAM enzyme (Holliday et al., 2018). For AhbD, it was proposed that the hydrogen atom abstractions from the substrate or monovinyl-intermediate occur at the β-positions (C3^1^ and C8^1^, Figure 1A) of the propionate side chains of pyrrole rings A and B (Bali et al., 2011), although there is no experimental evidence for this regiochemistry so far. The single electron of the resulting substrate or intermediate radical can delocalize over the iron-porphyrin ring system and the central metal ion (Figure 1B) (Kühner et al., 2016). The release of CO_2_ with formation of the vinyl group might be accompanied by the reduction of the central iron ion from the Fe(III) to the Fe(II) state representing the radical quenching step of the AhbD reaction. For the second decarboxylation reaction, the Fe(II) has to be reoxidized to Fe(III), and it was proposed that the two auxiliary [4Fe-4S] clusters within AhbD could be involved in electron transfer (Kühner et al., 2016; Fix et al., 2023). Overall, it was suggested that the central iron ion of FeCopro participates in AhbD catalysis by enabling electron transfer from the substrate radical to an electron acceptor (Kühner et al., 2016). This proposal was supported by the observation that substrate analogs such as coproporphyrin III (Copro) and zinc-coproporphyrin III (ZnCopro) served as very poor substrates resulting in strongly diminished amounts of decarboxylated reaction products. However, SAM cleavage and production of 5′-dA was observed in the presence of all three substrate analogs indicating that substrate radical formation was possible (Kühner et al., 2016).

In this study, we aimed to detect and characterize the substrate radical of the AhbD reaction. For this purpose, we performed electron paramagnetic resonance (EPR) measurements with samples containing purified AhbD, SAM, sodium dithionite and FeCopro or the substrate analogs Copro and ZnCopro. In the presence of the substrate analogs, signals for organic radicals were detected, which exhibited similar patterns of hyperfine splitting. Spectral simulations based on density functional theory (DFT) calculations were performed in order to assess the structure of the respective porphyrinyl radicals. The obtained results indicate that the hydrogen atom abstraction indeed takes place at the β-position of the propionate side chain. Moreover, the simulation of the spectrum obtained with ZnCopro suggests the presence of an electron donating ligand in proximity to the central metal ion.

## 2 Methods

### 2.1 Production and purification of recombinant AhbD

Recombinant AhbD from *Methanosarcina barkeri* was produced and purified as described previously (Fix et al., 2023). Protein concentrations were determined using the Bradford assay with bovine serum albumin as the standard according to the manufacturer’s instructions (Bio-Rad Laboratories GmbH, Feldkirchen, Germany).

### 2.2 Preparation of EPR samples

Samples for EPR measurements were prepared in an anaerobic chamber containing 95% N_2_ and 5% H_2_ (Coy Laboratory Products, Grass Lake, MI, United States). The samples contained purified AhbD (100 µM), substrate or substrate analog (100 µM), SAM (1 mM), and sodium dithionite (1 mM). After mixing, the sample solutions were transferred to EPR tubes in the anaerobic chamber and frozen in liquid nitrogen within two minutes. FeCopro, ZnCopro and Copro were purchased from Frontier Scientific Services Inc. (Newark, DE, United States) and dissolved as described previously (Kühner et al., 2016).

### 2.3 EPR spectroscopy

All EPR data sets were measured on a Bruker ELEXSYS E-500 EPR spectrometer with a Bruker ER 4122SHQ cavity and an Oxford Instruments ESR900 cryostat. All measurements were done at a temperature of 60 K with the following parameters: time constant 327.68 ms, conversion time 655.36 ms, modulation amplitude 0.2 mT, 1 mW microwave power and a microwave frequency of 9.377 GHz. Three scans were averaged per sample. The magnetic field was calibrated by using a Bruker DPPH sample ( g=2.0036 ). A manual baseline correction was applied afterwards.

### 2.4 EPR simulations

The EPR simulations were performed by using the EasySpin toolbox in the version 5.2.35 using the algorithm “pepper” (Stoll and Schweiger, 2006). The hyperfine couplings and the principle g values from the DFT calculations were used to perform the simulations. For the simulation, only the hydrogen (^1^H) and nitrogen couplings (^14^N) with a principle hyperfine coupling value larger than 8 MHz were considered. The linewidth was manually added to the simulation. All principle hyperfine coupling values and their corresponding Euler angles relative to the g -tensor frame were considered. As the only exception, the hyperfine coupling values of the methyl group hydrogens at C2 and C7 were averaged to the isotropic value to take the rotation of the CH_3_ group into account. Thus, the nine methyl group hydrogen principle hyperfine coupling values were averaged to an isotropic constant (see Supplementary Material). Additionally, the simulations were performed assuming a fixed methyl group by using the anisotropic hyperfine couplings (see Supplementary Material). All other values were ignored, since they are too small and contribute to the line width only. A simple Lorentzian line shape was used for all simulations. A second order perturbation theory for the hyperfine couplings was used to speed up the simulations.

### 2.5 DFT calculations

All DFT calculations (*in vacuo*) were performed by using the ORCA software package in the version 5.0.4 (Neese, 2012; 2018; Neese et al., 2020). The coproporphyrin III systems were computed and optimized in the following manner: The initial chemical structure of the coproporphyrin III radical was drawn by using ChemDraw22. The structure was geometrically optimized by an energy minimization using ChemDraw3D and an MM2 force field (Allinger, 1977). This structure was used for all following optimizations. Different radical positions were obtained by removing hydrogen on several positions manually. Each of the resulting structures was optimized in the following manner. First, a geometry optimization was applied with a B3LYP functional (Becke, 1988; Lee et al., 1988) and the def2-SVP basis set (Weigend and Ahlrichs, 2005; Weigend, 2006). The resulting structures were once more optimized using a B3LYP functional and the def2-TZVP basis set (Weigend and Ahlrichs, 2005; Weigend, 2006). The Hessian matrix was computed to validate the minimum of the optimization.

The hyperfine coupling tensors and the g -tensor values were computed by using a B3LYP functional and an EPR-II basis set (Barone, 1995). The center of the electron charge was used for the computation of the g -tensor.

The spin density plot was generated based on the B3LYP/EPR-II results. The figures were generated by using ChimeraX (Pettersen et al., 2004). The relative energy values of the different coproporphyrin III radicals were also computed and are shown in the Supplementary Figure S2. The zinc for the Zn-coproporphyrin III was also added manually. For the Zn-coproporphyrin III, all steps were done in the same way as described above, except for the hyperfine coupling tensors and the g -tensor values. Here, an aug-cc-pVTZ-J basis set was used for the Zn (Balabanov and Peterson, 2005; Hedegård et al., 2011), while the EPR-II basis set was kept for all other atoms. In this case, the spin density plot was generated based on the B3LYP/(EPR-II + aug-cc-pVTZ-J for Zn).

## 3 Results

### 3.1 Trapping the substrate radical of AhbD

In order to detect and characterize the substrate radical that is formed during AhbD catalysis, we first characterized a sample containing purified AhbD, FeCopro, SAM and sodium dithionite as reducing agent by EPR spectroscopy. However, for this sample, no radical signal could be observed. We hypothesized that the substrate radical (FeCopro^•^) was too short-lived to be trapped within the time frame of our experiment. Although the exact radical quenching mechanism for the substrate radical of AhbD is not known yet, it might either occur by electron transfer from the FeCopro^•^ ring system to an electron acceptor such as an auxiliary iron-sulfur cluster (Fix et al., 2023) or by reduction of the central iron ion of the FeCopro^•^ [Figure 1B, (Kühner et al., 2016)]. In both cases, electron transfer and decarboxylation might be quite fast, requiring rapid freeze-quench techniques to trap the substrate radical. Alternatively, if hydrogen atom abstraction and substrate radical formation takes place with Fe(III)Copro (d^5^ configuration for Fe^3+^), as previously proposed (Kühner et al., 2016), the resulting Fe(III)Copro^•^ could be EPR silent due to the integer spin of the overall system, although a triplet signal in low-spin configuration of Fe(III) or a septet signal in high-spin configuration of Fe(III) cannot be ruled out.

**FIGURE 2:**
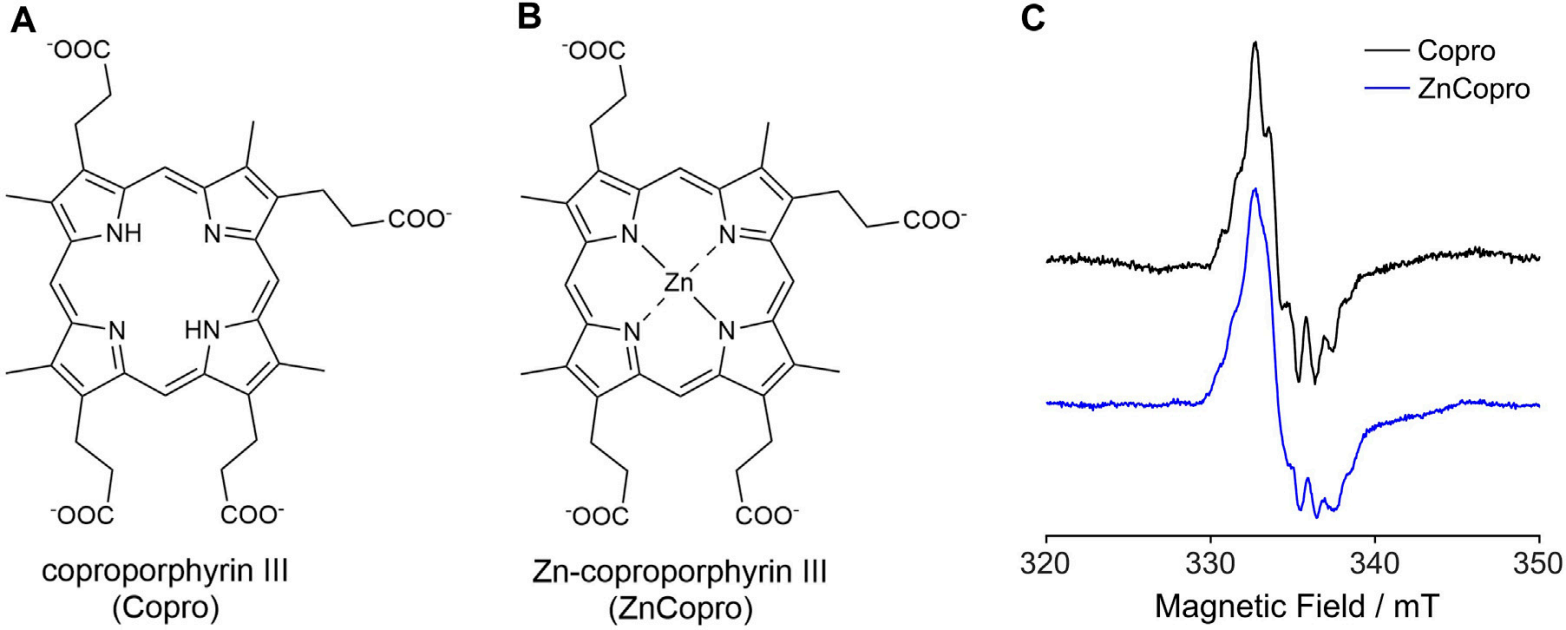
Structures of the substrate analogs coproporphyrin III **(A)** and Zn-coproporphyrin III **(B)**. **(C)** EPR spectra recorded at 60 K for samples containing purified AhbD, SAM, sodium dithionite and either Copro or ZnCopro. For detailed parameters see Methods section.

In order to trap the substrate radical, we employed two different substrate analogs either lacking the central iron ion (Copro; Figure 2A) or containing zinc as central metal ion (ZnCopro; Figure 2B). We have previously shown that these substrate analogs are hardly converted (decarboxylated) by AhbD to the reaction products. Nevertheless, SAM cleavage and formation of 5′-dA is observed in the presence of these substrate analogs (Kühner et al., 2016) suggesting that hydrogen atom abstraction and substrate radical formation might still take place. Therefore, samples containing purified AhbD, the respective substrate analog, SAM and sodium dithionite were prepared and EPR spectra were measured at 60 K with the parameters given in the Methods section. As shown in Figure 2C, EPR signals characteristic for organic radicals centered at g ≈ 2.00 were detected for both substrate analogs. The observed radical signals exhibit similar, but not identical hyperfine splitting patterns indicating coupling of the unpaired electron of the organic radical with several hydrogen or nitrogen nuclei of the tetrapyrrole. In the case of the Copro^•^ signal, the hyperfine splitting pattern is slightly more resolved compared to that of the ZnCopro^•^ signal. This might be caused by faster relaxation of the ZnCopro^•^ compared to Copro^•^, inducing line broadening. In order to explain the observed radical signals and their hyperfine splitting patterns in more detail, DFT calculations and spectral simulations were performed.

### 3.2 DFT calculations and simulation of EPR spectra for Copro^•^

#### 3.2.1 Site of hydrogen atom abstraction

**FIGURE 3:**
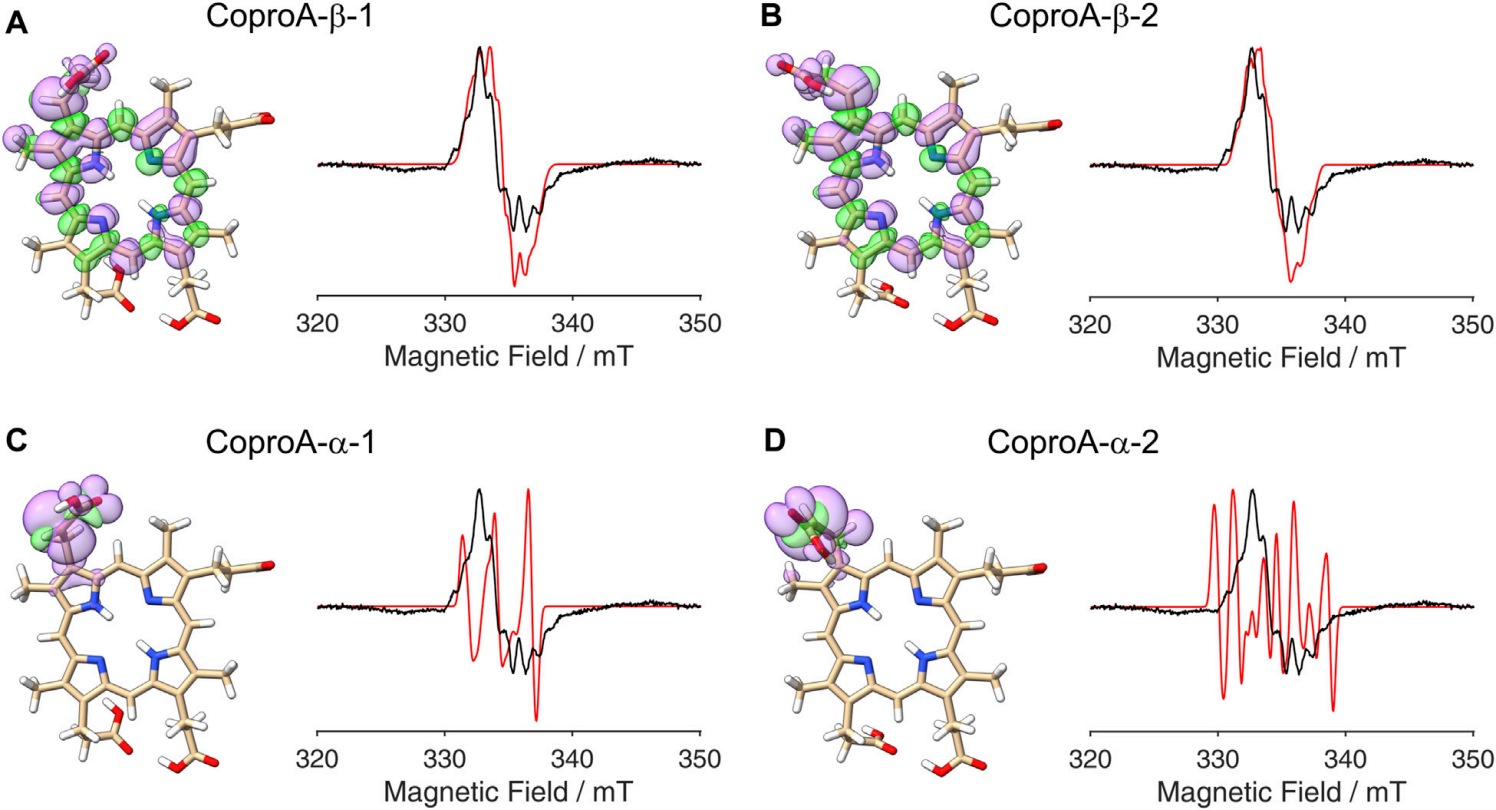
DFT calculated spin density plots and simulation of EPR spectra for hydrogen atom abstraction at either the α- or the β-position of the ring A propionate side chain of Copro. Positive spin density is shown in purple, negative spin density is depicted in green, both at a cut off level $\sigma =\pm 4.5\cdot 10^{-4}$ . The experimental EPR spectrum is shown in black, the simulated spectra are depicted in red. **(A)** Hydrogen atom abstraction at the β-position with the remaining hydrogen pointing towards the methyl group (orientation 1). **(B)** Hydrogen atom abstraction at the β-position with the remaining hydrogen pointing towards the methine bridge (orientation 2). **(C)** Hydrogen atom abstraction at the α-position with the remaining hydrogen pointing towards the methyl group. **(D)** Hydrogen atom abstraction at the α-position with the remaining hydrogen pointing away from the methyl group.

So far, it was assumed that the hydrogen atom abstraction from the substrate during the AhbD reaction takes place at the β-position of the propionate side chains. This assumption is based on chemical logic as well as on the experimentally established reaction course of coproporphyrinogen III dehydrogenase (CgdH) (Seehra et al., 1983; Layer et al., 2006), a Radical SAM enzyme that catalyzes the oxidative decarboxylation of propionate side chains of coproporphyrinogen III within the protoporphyrin-dependent heme biosynthesis pathway (Layer, 2020). In contrast to CgdH, the regiochemistry of the hydrogen atom abstraction has not been experimentally established for AhbD so far. Therefore, we sought to obtain insight into this aspect of AhbD catalysis by analyzing the EPR signal observed for Copro^•^. For this purpose, DFT calculations and spectral simulations were performed as described in the Methods section. For the calculations, it was assumed that the hydrogen atom abstraction takes place either at the β- (C3^1^) or the α-position (C3^2^) of the propionate side chain of pyrrole ring A, and the corresponding calculations were termed CoproA-β and CoproA-α, respectively. For each Copro^•^, two possible orientations of the radical were calculated. The DFT calculated spin density plots and spectral simulations are shown in Figure 3. For the CoproA-β radicals, there is spin density predicted all over the porphyrin ring system. Considerable spin density is also present on the propionate side chain as well as on the neighboring methyl group on pyrrole ring A (Figures 3A, B). Comparing the CoproA-β with the CoproA-α radicals, it is obvious that there is more spin density on the propionate carboxyl group and almost no density above the set threshold within the porphyrin ring system for both CoproA-α radicals (Figures 3C, D). For the simulation of the EPR spectra, the g -tensor and the hyperfine couplings were used as described in Section 2.4 and are listed in the Supplementary Material. Overall, the spectral simulations for the CoproA-β radicals are in reasonable agreement with the experimental spectrum, whereas the simulations for the CoproA-α radicals exhibit a clearly different hyperfine splitting pattern that does not fit the experimental data. The same is true for simulations of potential CoproB-α radicals (Supplementary Material; Supplementary Figure S1). From these results, we conclude that the site of hydrogen atom abstraction during AhbD catalysis is the β-position of the propionate side chain. In line with this proposal, the CoproA/B-β radicals are energetically preferred compared to the CoproA/B-α radicals based on the DFT calculations with a computed energy difference of roughly 40–50 kJ/mol (Supplementary Material; Supplementary Figure S2).

#### 3.2.2 Sequence of side chain decarboxylations

**FIGURE 4:**
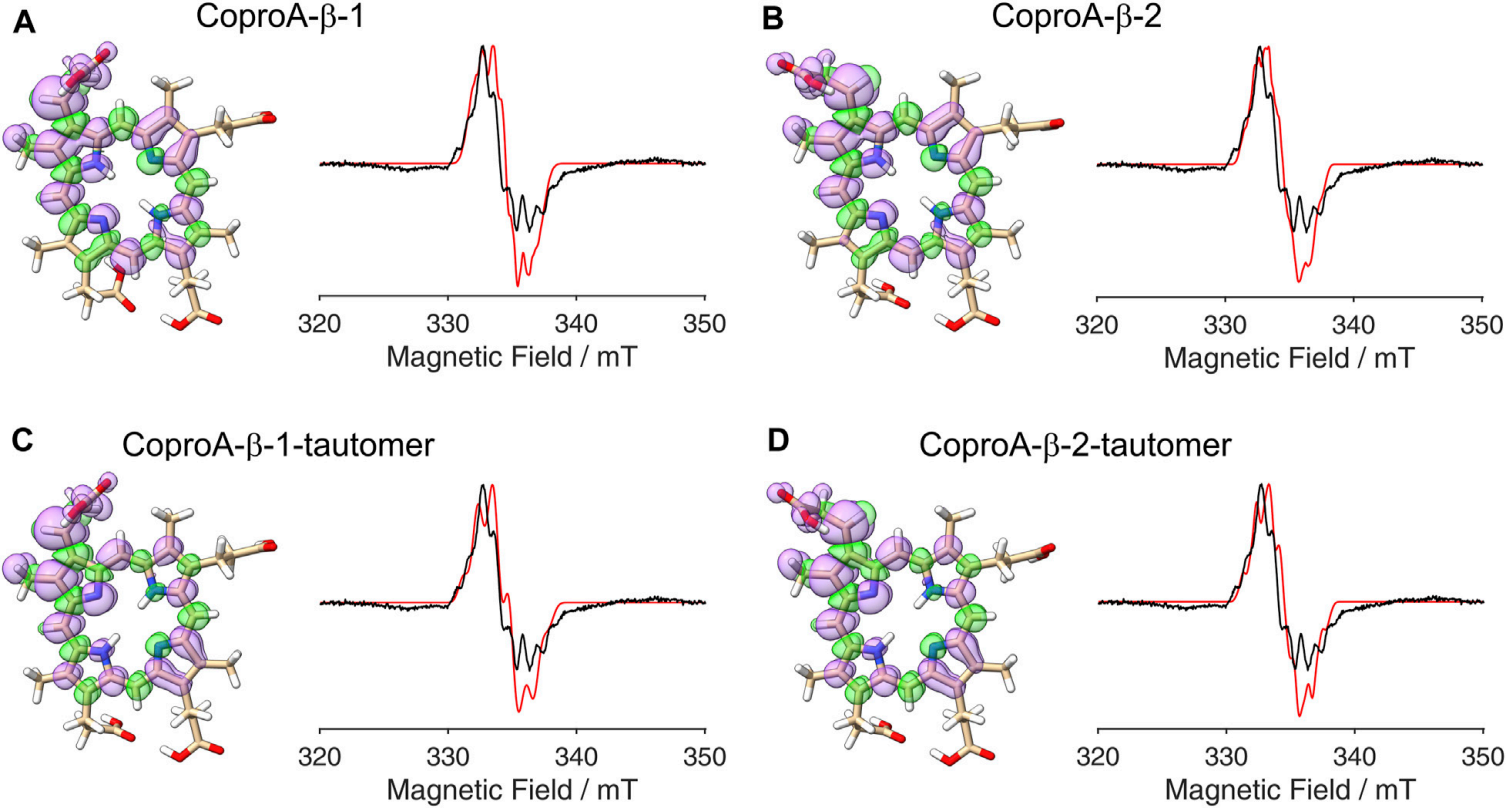
DFT calculated spin density plots and simulation of EPR spectra for hydrogen atom abstraction at the β-position of the ring A propionate side chain of Copro. Positive spin density is shown in purple, negative spin density is depicted in green, both at a cut off level $\sigma =\pm 4.5\cdot 10^{-4}$ . The experimental EPR spectrum is shown in black, the simulated spectra are depicted in red. **(A)** The remaining β-hydrogen of the radical points towards the methyl group (orientation 1). **(B)** The remaining β-hydrogen of the radical points towards the methine bridge (orientation 2). **(C, D)** Same as **(A, B)**, respectively, but with exchange of the N-H groups.

**FIGURE 5:**
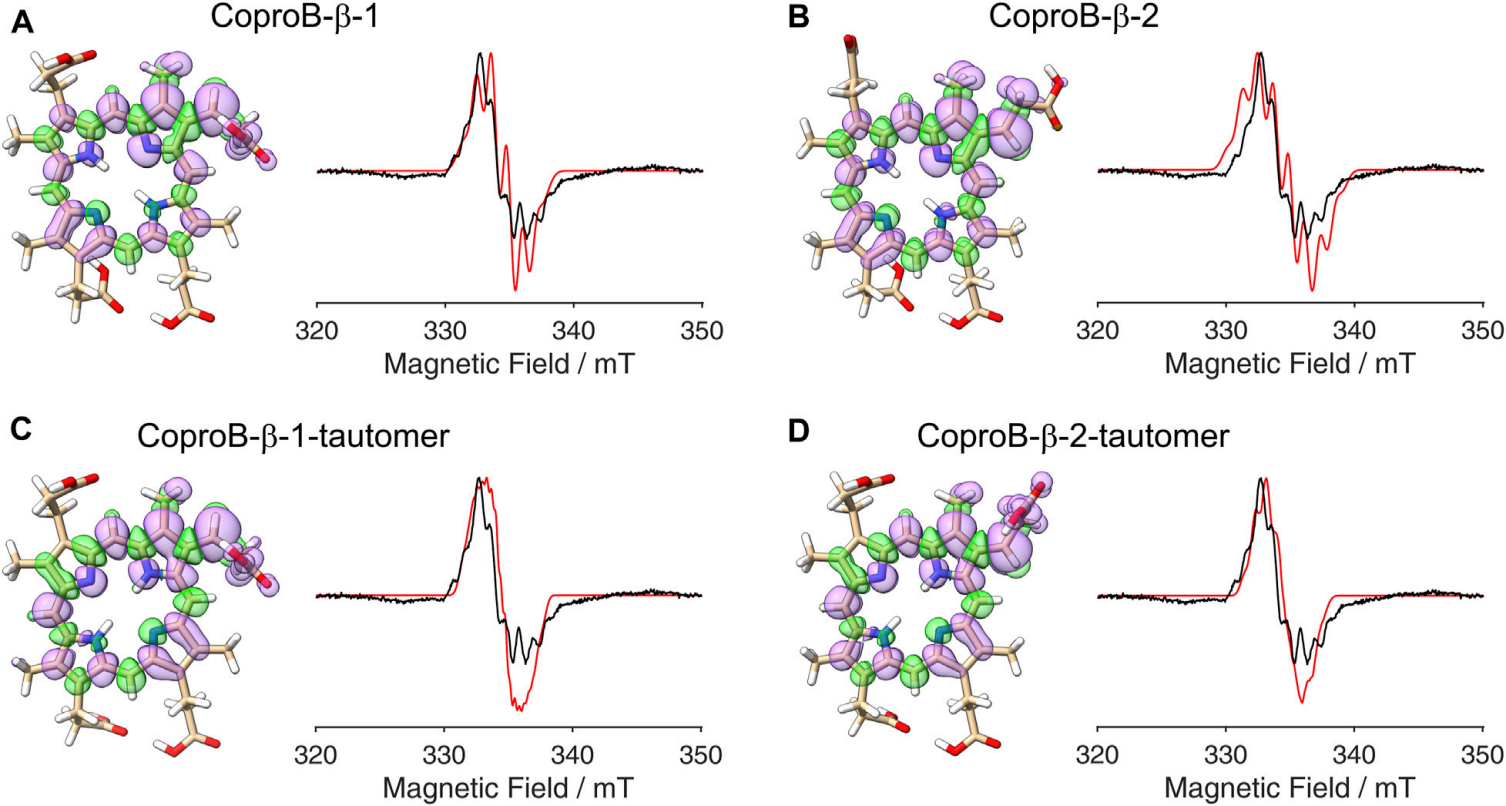
DFT calculated spin density plots and simulation of EPR spectra for hydrogen atom abstraction at the β-position of the ring B propionate side chain of Copro. Positive spin density is shown in purple, negative spin density is depicted in green, both at a cut off level $\sigma =\pm 4.5\cdot 10^{-4}$ . The experimental EPR spectrum is shown in black, the simulated spectra are depicted in red. **(A)** The remaining β-hydrogen of the radical points towards the methyl group (orientation 1). **(B)** The remaining β-hydrogen of the radical points towards the methine bridge (orientation 2). **(C, D)** Same as **(A, B)**, respectively, but with exchange of the N-H groups.

After establishing the β-position as the site of hydrogen atom abstraction, we wondered whether it was also possible to determine the propionate side chain (ring A or B), on which the radical is formed, by EPR spectroscopy and spectral simulations. This would shed light on the so far unknown sequence of side chain decarboxylations. For this purpose, DFT calculations and spectral simulations were performed as described above with hydrogen atom abstraction at the β-position of the propionate side chains on pyrrole rings A and B. Again, both orientations of the radical with the remaining β-hydrogen pointing either towards the methyl group or the methine bridge position were considered. Additionally, calculations were also performed for the corresponding tautomeric forms, in which the pyrrole N-H groups are exchanged, resulting in a total of eight simulations. The DFT calculated spin density plots and spectral simulations for hydrogen atom abstraction at the propionate side chain on ring A are shown in Figure 4 and those for the propionate side chain on ring B are depicted in Figure 5. In all cases, spin density is distributed over the porphyrin ring system as well as on the propionate side chain and the neighboring methyl group of the same pyrrole ring. The g -tensors and the hyperfine couplings used for the simulations of the EPR spectrum are listed in the Supplementary Material. For the methyl group, it was assumed that free rotation is possible, and the methyl group hydrogen hyperfine coupling values were averaged to an isotropic constant. For comparison, simulated spectra assuming a fixed methyl group are shown in the Supplementary Figures S3, S4. Since the surrounding amino acid environment of coproporphyrin III within the active site of the enzyme is not known, it cannot be stated, if the methyl group is freely rotating, hindered rotating or fixed at 60 K. Overall, the obtained results show that the simulated spectra are all different when compared to each other. Therefore, the orientation of the radical, the conformation of the propionate side chain, the tautomeric form of the porphyrin macrocycle and the rotation of the methyl group all influence the hyperfine splitting pattern of the EPR spectrum. None of the simulated spectra come close enough to the experimental spectrum. Additional DFT calculations for Copro with deprotonated carboxylate groups did also not result in more accurate simulated spectra. This can be explained by the fact that the DFT calculations were done for “free” Copro (*in vacuo*), while the experimental spectrum represents enzyme-bound Copro. Due to the lack of a structure of the AhbD-substrate complex that would reveal *inter alia* the side chain conformations of the enzyme-bound Copro, it is not possible to take the enzyme’s influence on the spin density distribution into account. Nevertheless, several of the simulations represent the experimental data reasonably well showing a good agreement of the hyperfine splitting pattern. However, as this holds true independent of the position of the side chain, it is not possible to distinguish between the propionate group of ring A or B by means of comparing EPR spectra with models obtained by DFT calculations.

### 3.3 DFT calculations and simulation of EPR spectra for ZnCopro^•^

**FIGURE 6:**
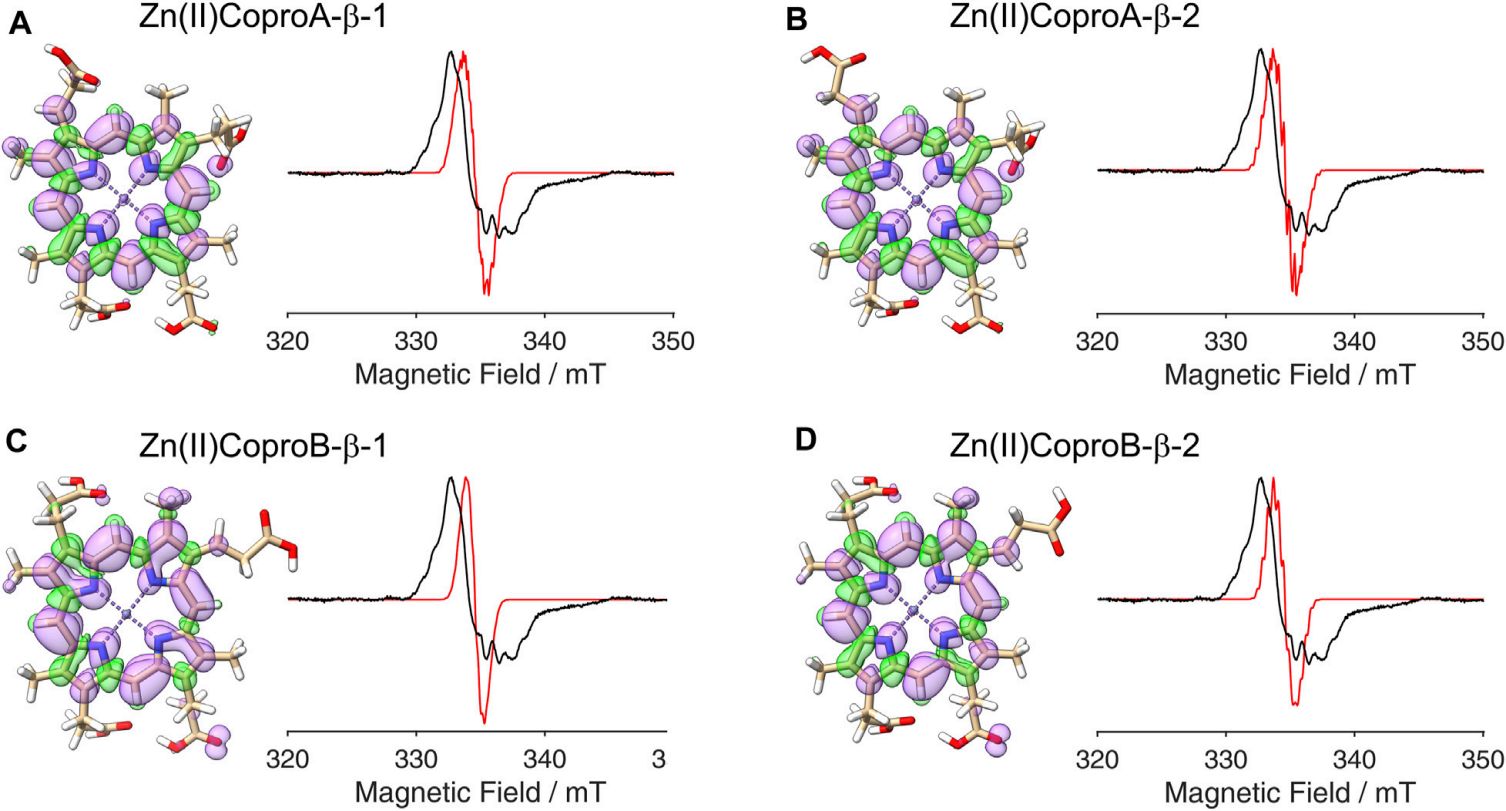
DFT calculated spin density plots and simulation of EPR spectra for hydrogen atom abstraction at the β-position of either the ring A or ring B propionate side chain of Zn(II)Copro. Positive spin density is shown in purple, negative spin density is depicted in green, both at a cut off level $\sigma =\pm 4.5\cdot 10^{-4}$ . The experimental EPR spectrum is shown in black, the simulated spectra are depicted in red. **(A)** Hydrogen atom abstraction at the ring A propionate with the remaining β-hydrogen pointing towards the methyl group (orientation 1). **(B)** Hydrogen atom abstraction at the ring A propionate with the remaining β-hydrogen pointing towards the methine bridge (orientation 2). **(C)** Hydrogen atom abstraction at the ring B propionate with the remaining β-hydrogen in orientation 1. **(D)** Hydrogen atom abstraction at the ring B propionate with the remaining β-hydrogen in orientation 2.

**FIGURE 7:**
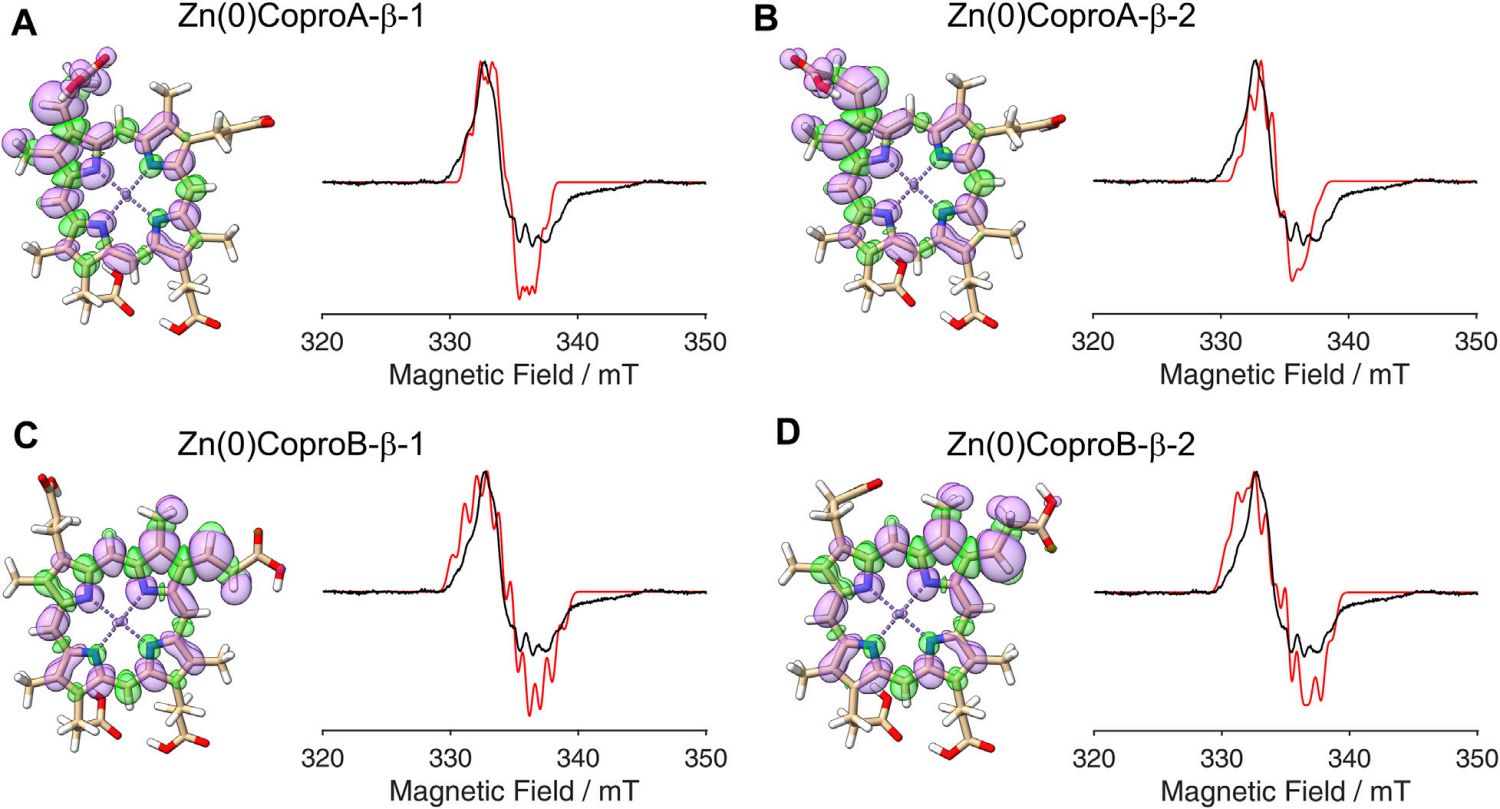
DFT calculated spin density plots and simulation of EPR spectra for hydrogen atom abstraction at the β-position of either the ring A or ring B propionate side chain of Zn(0)Copro. Positive spin density is shown in purple, negative spin density is depicted in green, both at a cut off level $\sigma =\pm 4.5\cdot 10^{-4}$ . The experimental EPR spectrum is shown in black, the simulated spectra are depicted in red. **(A)** Hydrogen atom abstraction at the ring A propionate with the remaining β-hydrogen pointing towards the methyl group (orientation 1). **(B)** Hydrogen atom abstraction at the ring A propionate with the remaining β-hydrogen pointing towards the methine bridge (orientation 2). **(C)** Hydrogen atom abstraction at the ring B propionate with the remaining β-hydrogen in orientation 1. **(D)** Hydrogen atom abstraction at the ring B propionate with the remaining β-hydrogen in orientation 2.

The radical signal detected in the sample containing ZnCopro exhibits a similar pattern of hyperfine splitting compared to the Copro^•^ signal, albeit less resolved (Figure 2C). Again, DFT calculations and spectral simulations were performed, in this case with Zn^2+^ as central metal ion and hydrogen atom abstraction from the β-position of either the ring A or ring B propionate side chain. As with Copro^•^, two possible orientations of the radical were considered for each side chain (Figure 6; Supplementary Material). The comparison of the spin density plots of the Zn(II)Copro radicals with those of the Copro radicals (Figures 4, 5) reveals that the presence of the Zn^2+^ ion draws spin density into the porphyrin ring system resulting in diminished spin density on the propionate side chain as expected. The DFT calculations of the Zn(II)Copro radicals show smaller hyperfine coupling values and, accordingly, the corresponding spectral simulations of the Zn(II)Copro radicals exhibit a narrower hyperfine splitting pattern than those of the Copro radicals and the experimental signal of the ZnCopro^•^. Again, it must be noted that the calculations were performed for “free” Zn(II)Copro and, therefore, any influence of the protein environment on the enzyme-bound Zn(II)Copro is not reflected by these calculations. For example, an electron donating ligand in proximity to the central metal ion could diminish the electron drawing effect of the Zn^2+^ that is observed in the DFT calculated spin density plots. In order to test this hypothesis, we performed the same calculations as before, but assuming Zn^0^ as the central metal. The corresponding spin density plots show that the density drawing effect of the metal is abrogated in this scenario (Figure 7; Supplementary Material). Here, the DFT calculations of the Zn(0)Copro radicals show larger hyperfine coupling values. The corresponding simulated EPR spectra of the Zn(0)Copro radicals exhibit a wider hyperfine splitting pattern compared to the Zn(II)Copro radicals and reflect the experimental spectrum better than those of the Zn(II)Copro radicals. Based on these results, we propose that an electron donating ligand is located in proximity to the central Zn^2+^ ion of the porphyrin contributing to substrate binding within the enzyme.

## 4 Discussion

Substrate radicals have been detected by EPR spectroscopy for a number of Radical SAM enzymes (Ballinger et al., 1992; Wu et al., 1995; Wu et al., 2000; Layer et al., 2006; Yokoyama et al., 2008; Grove et al., 2013; Lanz et al., 2015; Lilla and Yokoyama, 2016; Balo et al., 2021). In general, a prerequisite for the observation of an organic radical is its sufficiently long lifetime. For some Radical SAM enzymes, the respective substrate radicals are stabilized by delocalization of the unpaired electron into a system of conjugated double bonds present in the natural substrates (Layer et al., 2006; Grove et al., 2013; Balo et al., 2021). In some cases, double bond containing substrate analogs were used in order to stabilize the substrate radical (Wu et al., 2000; Lanz et al., 2015). Alternatively, interrupting the radical quenching step can also offer a strategy for radical trapping. For this purpose, suitable enzyme variants can be used, and such experiments provide insights into the fate of the substrate radical (Lilla and Yokoyama, 2016). In the case of AhbD, no substrate radical was detected with the natural substrate FeCopro, either due to a rapid radical quenching step or due to the transient formation of an EPR silent system. In contrast, radical species were observed, when the substrate analogs Copro and ZnCopro were used as shown here. In both cases, the formed radicals are EPR detectable and the rate of the radical quenching step might be slower than in the presence of the natural substrate. The proposed abstraction of a hydrogen atom at the β-position of the propionate side chain, either at C3^1^ or at C8^1^, leads to the formation of a substrate radical that, in principle, can delocalize into the porphyrin ring system due to its conjugated double bonds. The DFT calculations described in this study indeed reveal a spin density distribution all over the porphyrin ring system. However, our DFT calculations show that the unpaired electron is partly localized on the propionate side chain, at which the hydrogen atom abstraction takes place, as well as on the neighboring methyl group of the same pyrrole ring. Radical quenching by electron transfer from these side chains or from the porphyrin ring system to an electron acceptor seems to be unfavorable considering that Copro and ZnCopro are very poor substrates for AhbD (Kühner et al., 2016). Thus, we propose that the actual radical quenching mechanism is interrupted in AhbD when using the substrate analogs, which might be the reason for relatively stable Copro and ZnCopro radicals. Therefore, the results of this study strongly support the hypothesis that the central iron ion of FeCopro is required for efficient radical quenching.

The second important observation of this study is the clear difference in the simulated spectra depending on the site of hydrogen atom abstraction (α- or β-position). Based on this difference, we are able to assign the β-position as the site of hydrogen atom abstraction during the AhbD reaction, which was previously unknown. In contrast, we were not able to determine which of the two propionate side chains (ring A or B) is decarboxylated first. In order to further investigate this question, ^2^H- or ^13^C-labeled (Zn)Copro could be used. However, the selective labeling of either the C3 (ring A) or the C8 (ring B) propionate side chain, if possible at all, is not a trivial task.

Finally, the DFT calculations and spectral simulations of the ZnCopro^•^ suggest the presence of an electron donating ligand in proximity to the central metal ion. Although we created an AlphaFold2 model of AhbD from *M. barkeri* previously (Fix et al., 2023), this model did not include the bound substrate. Substrate docking trials using the Webina web application (Durrant lab, University of Pittsburg) roughly revealed the substrate binding site, however, no direct ligand to the central metal ion could be determined. Therefore, future studies including structure determination of an AhbD-substrate complex will provide further insight into substrate binding and the enzyme’s influence on the electronic properties of the substrate.

## Data Availability

The original contributions presented in the study are included in the article/Supplementary Material, further inquiries can be directed to the corresponding authors.
