# Supplementary material for: Trapping the substrate radical of heme synthase AhbD
Source: Front Chem. 2024 Jul 25;12:1430796. doi: 10.3389/fchem.2024.1430796 (PMC11306076; doi:10.3389/fchem.2024.1430796)
Supplement: Supplementary file 1 [file DataSheet1.PDF]

Supplementary Material

1 Supplementary Data

DFT Computational results and values for EPR simulation

In this section (pages 2-61), all hyperfine couplings and g-values from the DFT are given. All values are copied from the ORCA output files. The numbers of the atoms are illustrated in the respective pictures of the molecules.

The values which were used for the EPR simulation are highlighted:

Principle g-values in cyan.

Principle hyperfine tensor values and the corresponding Euler angles with respect to the g-tensor principle axis in yellow.

Hyperfine coupling of the CH_3 group in green.

CoproA-β-1

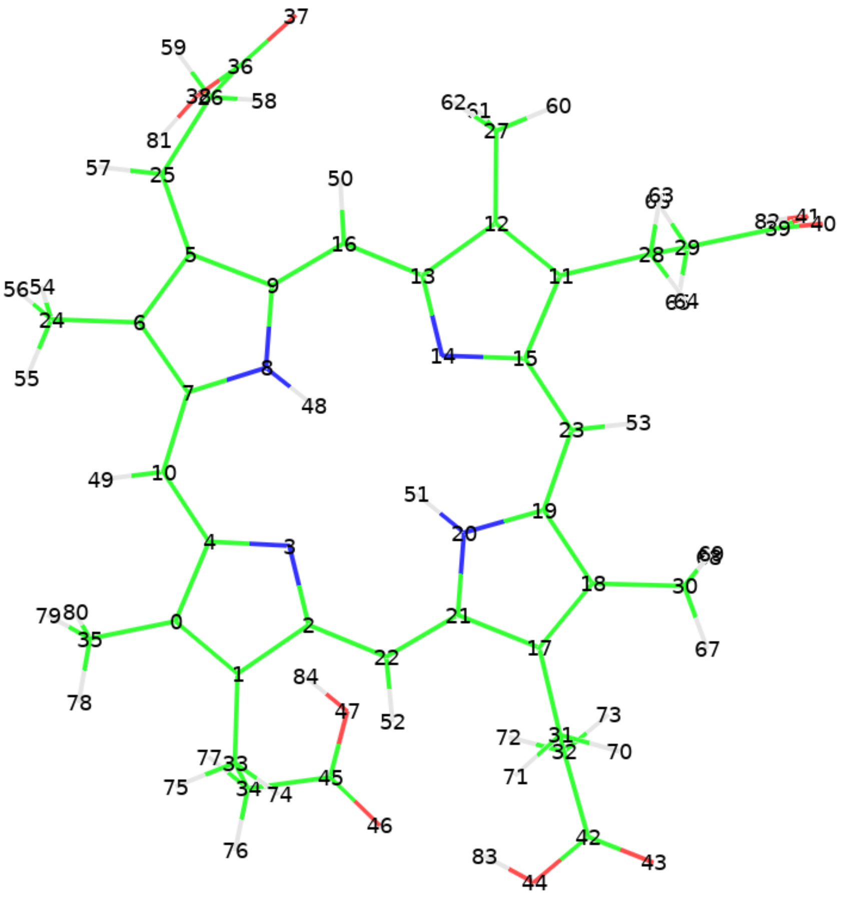


```
--------------------
ELECTRONIC G-MATRIX
--------------------

The g-matrix:
                 2.0031826   -0.0003925   -0.0000680
                -0.0000233    2.0029774    0.0001142
                 0.0001157   -0.0000121    2.0025267

 gel             2.0023193    2.0023193    2.0023193
 gRMC           -0.0001567   -0.0001567   -0.0001567
 gDSO(tot)       0.0003193    0.0003597    0.0001190
 gPSO(tot)       0.0000353    0.0003354    0.0010303
                ----------   ----------   ----------
 g(tot)          2.0025172    2.0028576    2.0033119 iso=  2.0028956
 Delta-g         0.0001979    0.0005383    0.0009927 iso=  0.0005763

Orientation:
  X             -0.0809998    0.5225703   -0.8487398
  Y             -0.1459442    0.8361414    0.5287417
  Z              0.9859712    0.1666967    0.0085388
```

Euler rotation of hyperfine tensor to g-tensor

Atom	Alpha	Beta	Gamma	Ax	Ay	Az
		[degrees]			[MHz]	
0C	122.4	31.2	-136.8	1.79	2.02	1.71
1C	61.5	11.8	-63.9	-3.98	0.41	0.45
2C	-76.7	27.5	76.7	-10.92	-4.88	-3.79
4C	-31.5	5.5	30.4	-20.18	-6.96	-5.89
5C	85.6	42.4	-88.0	-53.68	-25.49	-29.65
6C	81.1	20.4	-82.5	82.23	6.42	5.37
7C	-85.0	42.3	84.9	-34.25	-16.21	-14.46
9C	-16.9	4.1	16.0	22.02	2.61	3.45
10C	84.1	44.6	-87.2	33.02	3.23	3.53
11C	83.9	40.8	-86.4	-0.32	-2.62	-2.34
12C	-36.1	5.8	34.4	3.31	-1.80	-1.93
13C	-82.5	38.3	82.6	18.92	3.60	2.71
15C	70.7	15.0	-73.5	20.93	3.39	2.86
16C	83.8	33.7	-85.3	-18.84	-4.89	-3.50
17C	-84.7	18.0	84.0	11.48	1.12	1.42
18C	-83.6	25.2	83.4	-9.16	-3.17	-3.15
19C	-66.3	9.3	64.8	19.13	2.08	2.44
21C	-87.4	42.8	86.1	-6.88	-2.56	-2.84
22C	-81.0	37.9	80.4	15.82	0.69	1.16
23C	82.1	39.9	-85.7	-18.36	-3.79	-4.71
24C	-80.8	40.9	79.8	-11.75	-10.68	-11.79
25C	-85.2	44.6	83.9	131.25	7.29	6.20
26C	-106.0	5.2	99.8	-20.25	-18.66	-21.05
27C	-162.7	40.1	166.6	-1.00	-0.85	-0.96
28C	-101.2	44.6	108.9	-0.50	-0.33	-0.56
29C	46.0	45.2	-9.7	1.36	1.00	1.06
30C	36.9	11.7	-58.8	0.81	0.78	0.90
31C	-100.0	24.2	86.9	-1.52	-1.63	-1.42
32C	-47.2	29.8	19.5	2.89	2.18	2.23
33C	-67.3	31.4	66.5	0.62	0.76	0.70
34C	-38.9	33.4	22.3	-1.40	-1.09	-1.13
35C	70.7	19.6	-72.2	-0.12	0.23	-0.05
36C	-114.5	34.5	69.7	50.90	43.12	43.43
39C	61.6	36.9	-97.7	0.10	0.10	0.21
42C	-54.2	39.8	68.3	0.25	0.23	0.38
45C	156.1	47.9	-137.6	0.06	0.17	0.04
48H	-64.9	8.2	65.1	-6.61	-8.22	0.21
49H	-83.5	45.0	83.8	-10.04	-4.39	-12.79
50H	91.3	36.1	-86.6	2.21	1.25	8.92
51H	78.2	26.2	-80.5	-0.63	0.14	1.06
52H	82.6	44.2	-87.0	-4.70	-1.70	-7.25
53H	82.2	37.7	-85.6	3.48	6.69	2.35
54H	61.2	45.0	-63.5	26.27	28.05	32.03
55H	-81.5	30.3	80.9	-0.71	3.90	-0.32
56H	111.3	42.3	-112.9	26.97	28.74	32.68

$A_{iso} = 19.7$ MHz

57H	83.2	25.7	-87.3	-38.13	-57.88	-16.50
58H	-145.2	32.4	134.2	38.94	46.34	39.42
59H	50.0	30.3	-55.2	3.53	11.08	2.79
60H	-92.8	19.2	93.0	-0.40	0.48	-0.16
61H	-72.2	26.1	54.7	2.91	3.84	3.53
62H	93.4	41.3	-81.0	2.01	2.76	3.08
63H	-78.1	36.7	84.4	-0.05	0.73	0.05
64H	74.4	29.9	-70.3	0.24	0.18	1.13
65H	-176.8	33.4	-138.7	-0.32	-0.18	0.44
66H	-72.1	38.2	37.0	-0.34	0.32	-0.22
67H	78.2	21.6	-79.6	-0.46	-0.36	-0.11
68H	85.3	18.1	-112.9	-2.51	-2.46	-2.10
69H	99.8	22.9	-63.3	-2.00	-1.96	-1.56
70H	-26.8	13.8	55.3	0.98	0.95	1.72
71H	-72.8	40.5	71.2	1.38	1.59	2.24
72H	0.6	44.5	-43.9	0.10	-0.57	-0.28
73H	137.7	38.1	177.7	-0.46	-0.30	0.20
74H	-13.0	18.7	6.3	-0.76	-0.16	-0.81
75H	88.2	35.2	-91.0	-0.75	-0.53	-0.63
76H	43.7	14.8	-33.6	-0.17	0.02	-0.09
77H	117.4	27.7	-115.6	-0.09	0.28	0.08
78H	140.5	29.7	-148.5	-0.08	0.46	-0.09
79H	163.8	26.1	-177.5	-0.97	0.27	-1.06
80H	15.4	44.1	-2.5	-1.14	-0.02	-1.23
81H	-105.8	14.2	75.0	7.17	-7.43	-9.70
82H	121.0	43.9	-125.6	-0.08	-0.10	0.16
83H	-89.4	44.8	110.9	-0.13	-0.08	0.18
84H	35.6	24.0	-1.3	-0.34	0.25	0.06
3N	-69.5	15.7	68.3	9.08	-0.11	0.07
8N	8.9	3.0	-9.6	7.17	-0.46	-0.24
14N	81.2	32.5	-84.3	-5.93	-0.46	-0.41
20N	56.3	6.1	-59.2	-0.99	-0.47	-0.43

CoproA-β-2

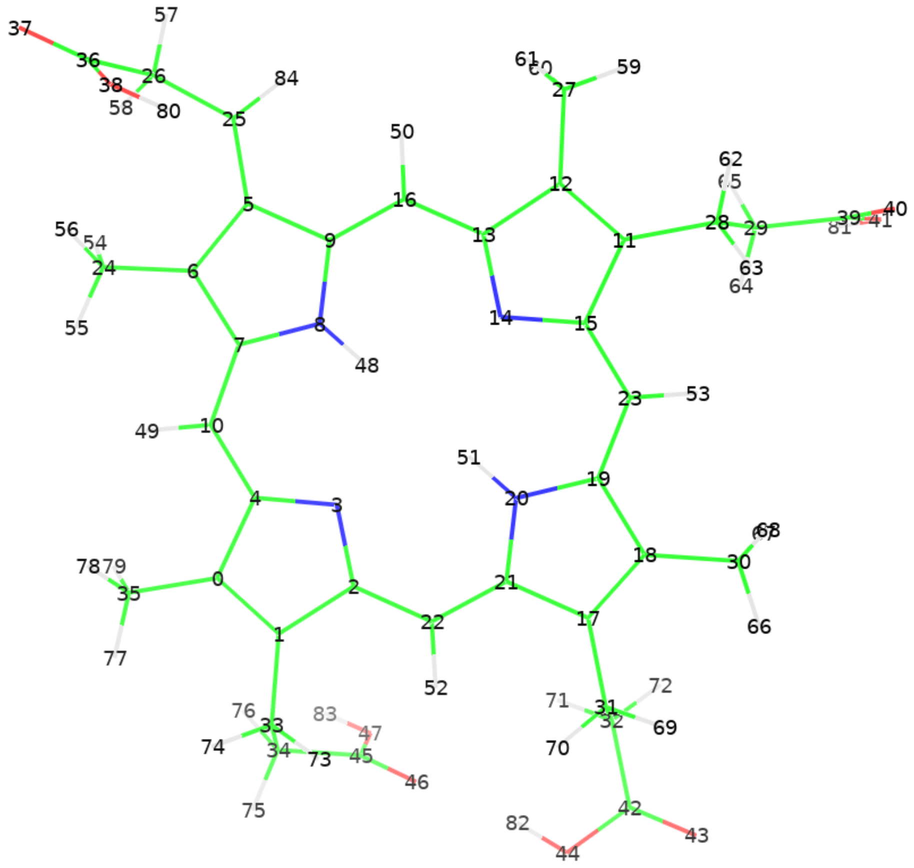

ELECTRONIC G-MATRIX

The g-matrix:

```
              2.0029884    0.0003496    0.0001311
              0.0002403    2.0032317    0.0002608
              0.0003552    0.0000742    2.0024728

 gel          2.0023193    2.0023193    2.0023193
 gRMC        -0.0001594   -0.0001594   -0.0001594
 gDSO(tot)    0.0002577    0.0001432    0.0002512
 gPSO(tot)   -0.0000445    0.0005133    0.0010923
             ----------   ----------   ----------
 g(tot)       2.0023731    2.0028164    2.0035034 iso=  2.0028976
 Delta-g      0.0000538    0.0004971    0.0011841 iso=  0.0005784
```

Orientation:

```
  X          -0.3389043    0.7488241    0.5695668
  Y          -0.0666798   -0.6229889    0.7793835
  Z           0.9384549    0.2261578    0.2610651
```

Euler rotation of hyperfine tensor to g-tensor

Atom	Alpha	Beta [degrees]	Gamma	Ax	Ay [MHz]	Az
0C	66.6	28.1	-82.4	2.27	1.57	1.86
1C	-29.6	11.2	18.6	-3.95	0.25	0.21
2C	-68.5	26.4	59.1	-8.95	-3.28	-4.25
4C	-10.2	9.3	-1.3	-18.32	-5.39	-6.33
5C	77.3	33.9	-92.1	-55.38	-30.97	-26.07
6C	68.1	21.7	-81.3	81.26	5.66	6.56
7C	-77.9	41.9	69.0	-33.20	-14.28	-15.91
9C	-24.8	8.5	11.7	22.40	3.20	2.33
10C	-76.2	39.6	67.4	30.50	2.97	3.23
11C	78.9	40.2	-94.9	-0.40	-2.15	-2.42
12C	-55.1	15.9	43.3	3.39	-1.63	-1.51
13C	-77.2	40.1	67.9	15.44	2.12	2.96
15C	60.9	18.7	-73.7	18.79	2.52	2.98
16C	75.8	36.2	-90.4	-14.83	-3.55	-5.01
17C	-67.1	18.0	52.8	10.15	1.19	0.92
18C	-39.7	8.8	25.7	-7.88	-2.81	-2.84
19C	-39.6	10.3	26.9	17.28	2.33	1.95
21C	78.2	44.0	-96.8	-5.45	-2.13	-2.37
22C	-73.6	34.0	64.3	13.06	0.84	0.44
23C	78.4	43.1	-92.9	-17.06	-4.25	-3.42
24C	-112.6	43.9	107.3	-10.68	-11.55	-10.47
25C	-72.3	21.9	62.4	136.07	6.17	7.81
26C	67.4	11.0	-71.6	-20.26	-19.08	-21.26
27C	-65.3	15.8	51.6	-0.94	-0.95	-0.80
28C	-99.4	43.2	72.1	-0.43	-0.50	-0.28
29C	160.4	34.8	-132.4	1.32	1.04	0.97
30C	27.8	27.7	-37.6	0.68	0.76	0.65
31C	-52.4	29.0	35.3	-1.37	-1.26	-1.46
32C	-15.6	31.7	30.0	2.67	2.08	2.03
33C	-60.5	23.9	59.6	0.57	0.65	0.70
34C	-0.8	27.7	9.5	-1.42	-1.20	-1.18
35C	66.7	21.6	-78.0	-0.22	-0.15	0.11
36C	120.4	12.8	-94.2	58.63	49.76	50.02
39C	128.1	44.6	-162.2	0.12	0.22	0.12
42C	-93.1	31.1	98.2	0.23	0.37	0.22
45C	148.3	29.5	-140.5	0.01	0.04	0.12
48H	-47.3	9.3	34.7	-6.63	0.28	-8.25
49H	-74.3	42.3	66.9	-9.40	-11.85	-4.08
50H	75.4	41.9	-89.8	0.90	8.05	0.29
51H	73.3	28.6	-86.2	-0.48	1.07	0.29
52H	-76.3	43.8	67.8	-4.02	-1.40	-6.13
53H	78.3	41.5	-92.6	3.27	2.19	6.27

54H	78.1	38.4	-64.9	36.17	41.24	37.54
55H	-77.4	26.1	57.7	0.41	0.91	4.87
56H	69.9	34.6	-102.9	23.22	28.53	25.31
57H	-151.9	36.3	155.0	9.76	17.78	10.11
58H	83.7	23.8	-83.8	27.51	34.92	29.11
59H	-66.6	19.7	53.7	-0.41	-0.22	0.45
60H	-39.4	31.9	39.9	2.51	3.08	3.53
61H	-102.5	29.0	81.8	2.02	2.64	3.09
62H	-80.6	35.1	74.5	-0.27	-0.19	0.46
63H	108.9	33.4	-119.9	0.49	1.33	0.43
64H	-115.8	39.2	159.5	0.48	-0.29	-0.12
65H	-157.4	42.2	-174.1	0.32	-0.21	-0.30
66H	71.6	21.9	-85.1	-0.42	-0.09	-0.33
67H	27.0	42.7	-46.9	-2.06	-1.68	-2.01
68H	139.2	38.8	-154.8	-1.62	-1.21	-1.58
69H	-158.6	27.3	153.4	1.01	1.70	0.99
70H	-73.9	37.3	71.5	1.03	1.81	1.21
71H	41.1	45.6	-13.9	0.11	-0.24	-0.49
72H	-85.8	42.8	125.2	0.15	-0.31	-0.44
73H	25.4	13.5	-16.8	-0.62	-0.63	-0.06
74H	71.6	38.4	-90.1	-0.79	-0.68	-0.55
75H	98.3	22.9	-102.8	-0.17	-0.10	0.01
76H	74.4	28.0	-105.2	-0.12	0.06	0.28
77H	93.6	21.8	-74.4	-0.09	-0.09	0.47
78H	77.2	21.5	-39.3	-0.73	-0.67	0.54
79H	147.3	11.7	-110.5	-0.77	-0.84	0.30
80H	111.7	11.0	-100.2	7.34	-8.03	-9.34
81H	41.8	45.9	-78.4	-0.08	0.15	-0.09
82H	-102.1	42.4	78.7	-0.13	0.19	-0.08
83H	146.5	32.9	-143.9	-0.12	-0.05	0.18
84H	-71.7	19.1	62.2	-39.31	-61.12	-15.84
3N	-56.5	17.0	46.1	8.07	0.05	-0.11
8N	-11.6	7.3	-1.3	7.22	-0.30	-0.49
14N	72.5	29.2	-86.7	-5.22	-0.37	-0.42
20N	21.1	9.7	-35.1	-1.13	-0.40	-0.45

A_{iso} = 22.0 MHz

CoproA-α-1

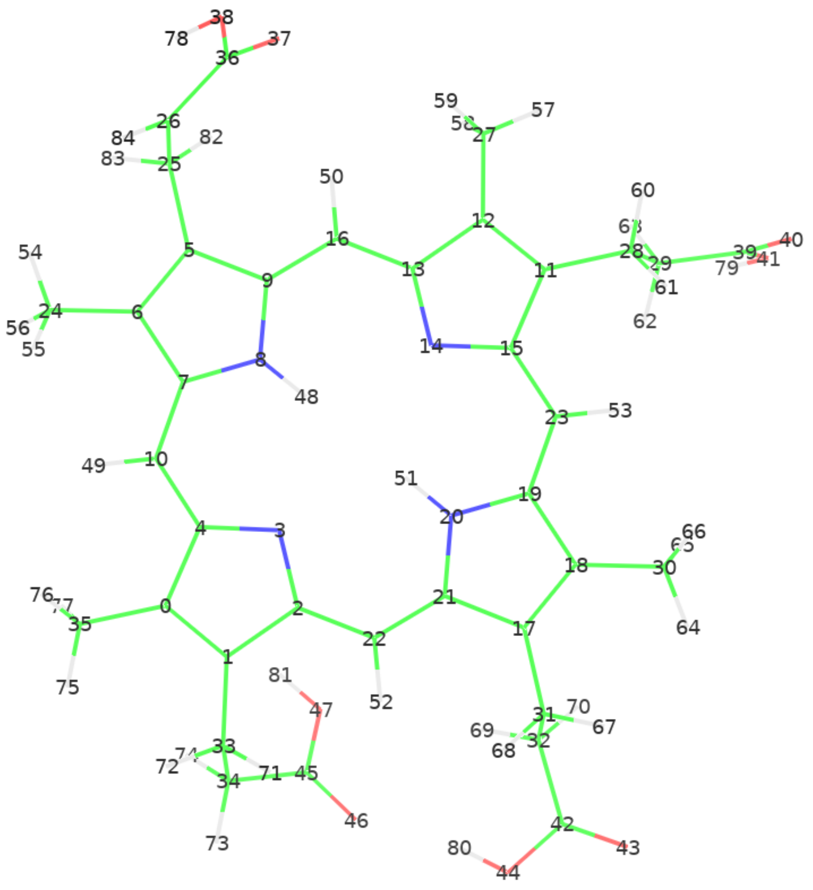


```
--------------------
ELECTRONIC G-MATRIX
--------------------

The g-matrix:
               2.0037905    0.0011507   -0.0003208
               0.0010940    2.0031599    0.0001488
              -0.0002205    0.0001788    2.0040114

 gel           2.0023193    2.0023193    2.0023193
 gRMC         -0.0001789   -0.0001789   -0.0001789
 gDSO(tot)     0.0001337    0.0004054    0.0004428
 gPSO(tot)    -0.0000142    0.0014929    0.0020800
              ----------   ----------   ----------
 g(tot)        2.0022599    2.0040387    2.0046632 iso=  2.0036539
 Delta-g      -0.0000594    0.0017194    0.0023439 iso=  0.0013347

Orientation:
  X            0.6020385    0.0502384   -0.7968850
  Y           -0.7810025    0.2446454   -0.5746162
  Z            0.1660865    0.9683103    0.1865222
```

Euler rotation of hyperfine tensor to g-tensor

Atom	Alpha [degrees]	Beta [degrees]	Gamma [degrees]	Ax [MHz]	Ay [MHz]	Az [MHz]
0C	149.4	51.4	-149.2	-0.09	0.16	0.01
1C	8.5	37.2	-16.6	-0.04	-0.05	-0.12
2C	36.1	33.0	-30.6	0.10	0.66	0.02
4C	15.4	46.1	-24.7	0.15	0.51	-0.01
5C	-153.7	35.5	110.2	44.26	56.79	44.02
6C	-140.2	39.2	129.8	-1.28	-0.31	0.49
7C	-22.1	34.0	13.9	2.26	1.74	1.33
9C	139.8	5.2	-126.5	-0.13	1.16	-1.31
10C	-140.5	46.9	112.9	-0.14	0.30	-0.18
11C	-132.4	49.0	126.0	0.05	-0.14	-0.10
12C	169.9	35.9	-149.8	0.20	-0.02	-0.13
13C	-124.8	50.9	136.7	0.27	-0.23	-0.19
15C	129.8	23.5	-134.5	0.08	0.35	-0.09
16C	-179.9	26.3	-143.0	0.30	-0.65	-0.82
17C	91.9	23.6	-72.6	-0.06	-0.09	-0.12
18C	110.8	20.8	-105.5	-0.01	0.17	-0.07
19C	97.7	19.8	-93.3	0.08	0.38	-0.02
21C	83.0	19.7	-75.7	0.12	0.69	0.04
22C	82.4	20.1	-75.7	-0.13	-0.63	-0.17
23C	114.5	20.1	-106.7	-0.04	-0.27	-0.15
24C	87.1	38.9	-106.5	-0.30	-0.58	0.18
25C	-151.2	4.0	169.1	-23.51	-24.59	-27.59
26C	-95.1	16.4	91.7	201.62	9.31	10.59
27C	9.3	43.0	13.4	-0.03	-0.06	0.43
28C	153.1	43.7	-140.3	0.07	-0.04	-0.04
29C	-16.9	45.6	17.2	0.02	0.01	0.10
30C	142.3	19.2	-131.7	-0.01	-0.05	-0.06
31C	83.6	37.1	-69.6	0.02	-0.02	-0.02
32C	90.2	41.6	-61.6	0.05	0.01	0.01
33C	-1.8	31.8	27.4	0.02	-0.04	-0.04
34C	17.1	27.5	21.9	0.08	0.04	0.03
35C	142.8	46.1	-138.5	-0.07	-0.07	0.01
36C	93.3	17.0	-98.7	-47.66	-40.14	-35.07
39C	173.3	43.8	-159.5	0.04	-0.02	-0.02
42C	5.8	5.7	15.3	0.02	-0.00	-0.00
45C	46.5	26.1	-13.8	0.03	-0.01	-0.01
48H	78.9	6.5	-56.1	1.33	-0.49	-0.40
49H	134.4	46.1	-131.7	-0.34	-0.39	0.70
50H	166.5	34.6	-129.0	4.34	-2.63	-1.01
51H	81.9	20.2	-64.2	0.46	-0.19	-0.13
52H	101.4	29.1	-87.8	0.24	0.03	0.11
53H	167.5	17.2	-157.9	0.29	-0.10	-0.09
54H	86.2	39.3	-86.9	-0.63	-0.90	3.19
55H	116.8	46.3	-102.4	-0.38	-0.50	1.32
56H	75.1	26.1	-113.5	0.53	0.68	2.92

57H	27.5	36.4	2.8	-0.22	-0.27	0.82
58H	6.6	37.1	33.6	-0.43	-0.49	1.20
59H	20.7	41.5	0.8	-0.95	-1.13	2.09
60H	155.2	48.8	-146.3	0.32	-0.18	-0.16
61H	143.7	43.7	-135.1	0.27	-0.11	-0.09
62H	166.0	37.3	-140.9	0.22	-0.11	-0.09
63H	-5.0	44.5	23.3	-0.12	-0.13	0.26
64H	105.3	43.4	-92.3	0.10	-0.05	-0.05
65H	-171.1	20.2	-175.4	0.27	0.09	0.10
66H	114.2	44.3	-105.3	0.24	0.04	0.06
67H	85.9	17.0	-74.8	0.09	-0.05	-0.05
68H	1.0	11.6	12.7	0.12	-0.04	-0.04
69H	-77.1	30.6	107.3	0.10	-0.04	-0.05
70H	98.1	20.9	-72.2	0.09	-0.05	-0.05
71H	-48.1	43.8	77.0	0.16	-0.04	-0.04
72H	17.3	39.1	4.4	0.15	-0.06	-0.05
73H	54.9	57.1	-35.5	0.09	-0.04	-0.04
74H	-127.6	36.5	85.8	-0.06	0.12	-0.06
75H	155.9	45.5	-140.9	-0.08	-0.09	0.18
76H	160.5	40.6	-142.2	-0.03	-0.06	0.36
77H	123.8	48.7	-128.7	0.04	0.02	0.38
78H	105.8	5.0	-109.6	-6.40	4.04	-4.24
79H	179.9	44.5	-153.6	0.13	-0.06	-0.06
80H	70.5	34.0	-46.2	0.06	-0.03	-0.03
81H	49.0	22.3	-7.4	0.15	-0.07	-0.08
82H	82.1	26.8	-83.0	-2.02	9.53	-1.49
83H	-154.8	41.2	175.8	75.81	87.38	74.39
84H	-93.6	23.4	89.9	-57.00	-87.47	-19.72
3N	55.8	40.5	-33.4	0.05	-0.07	-0.00
8N	3.2	28.9	-41.9	0.49	0.28	0.12
14N	-156.4	15.9	-160.6	0.05	0.01	-0.05
20N	89.0	19.3	-77.7	-0.01	-0.14	-0.05

CoproA-α-2

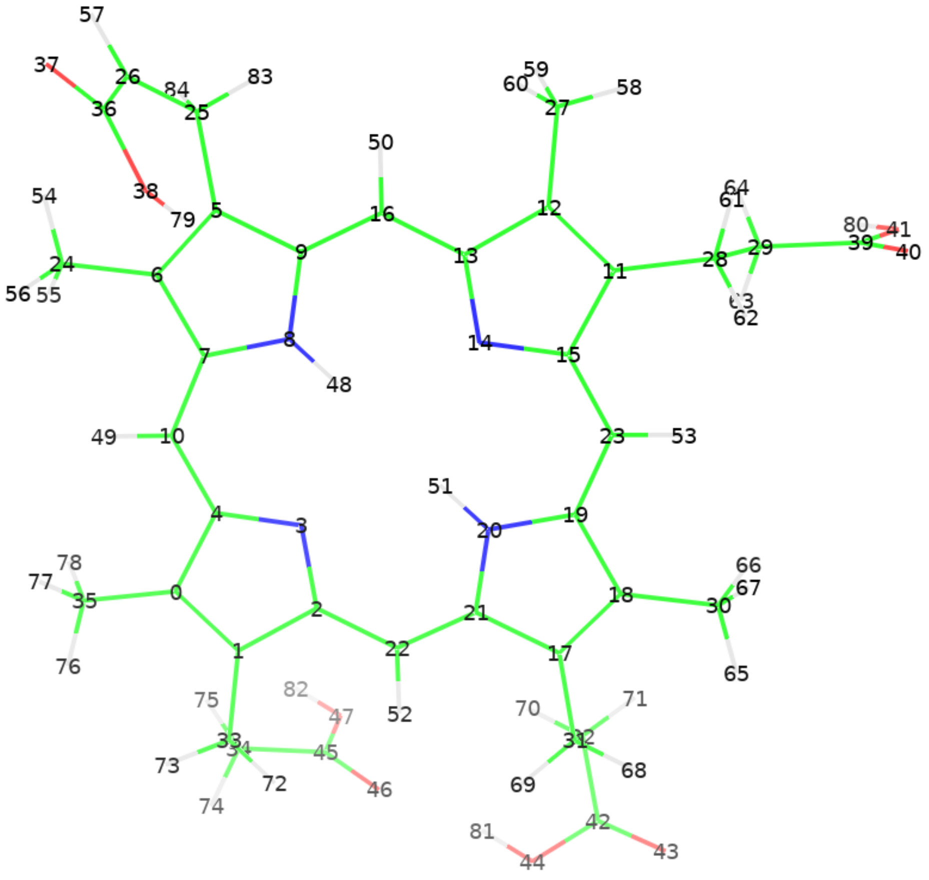

```
-------------------
ELECTRONIC G-MATRIX
-------------------

The g-matrix:
              2.0034642   -0.0007548   -0.0009351
             -0.0005819    2.0034311   -0.0001953
             -0.0010227    0.0004912    2.0055426

 gel          2.0023193    2.0023193    2.0023193
 gRMC        -0.0001770   -0.0001770   -0.0001770
 gDSO(tot)    0.0005369    0.0002157    0.0004856
 gPSO(tot)   -0.0000357    0.0014452    0.0033634
             ----------   ----------   ----------
 g(tot)       2.0026435    2.0038032    2.0059913 iso=  2.0041460
 Delta-g      0.0003242    0.0014839    0.0036720 iso=  0.0018267

Orientation:
  X          -0.7632049    0.5135225   -0.3921900
  Y          -0.6050205   -0.7810346    0.1547100
  Z          -0.2268669    0.3553584    0.9067810
```

Euler rotation of hyperfine tensor to g-tensor

Atom	Alpha	Beta	Gamma	Ax	Ay	Az
		[degrees]			[MHz]	
0C	-42.6	24.5	75.6	-0.02	0.08	-0.07
1C	-37.0	37.6	61.8	-0.00	0.07	-0.01
2C	-57.9	22.2	75.1	-0.08	0.01	-0.19
4C	-53.7	21.3	79.2	-0.09	0.08	-0.14
5C	-107.5	35.9	135.5	11.26	16.24	11.65
6C	-153.6	18.9	-165.7	-0.63	0.75	0.19
7C	-54.7	37.2	72.3	0.35	1.02	0.25
9C	-74.1	33.1	70.7	-1.18	-0.37	-1.39
10C	136.5	48.4	-167.3	0.24	-0.01	-0.11
11C	-89.5	31.0	54.9	-0.04	0.09	-0.01
12C	-95.6	27.6	57.6	-0.08	0.14	-0.11
13C	-92.0	39.8	66.4	0.09	0.48	0.13
15C	-139.8	29.9	121.0	-0.03	0.09	-0.05
16C	-35.5	35.9	26.5	0.87	1.49	0.54
17C	-78.3	32.0	78.0	0.01	0.05	0.04
18C	-81.0	23.7	75.3	-0.02	0.02	-0.07
19C	-75.4	25.3	65.7	-0.04	0.02	-0.05
21C	-67.5	23.2	73.3	-0.06	-0.01	-0.18
22C	-49.9	17.7	63.8	0.00	0.07	0.24
23C	-95.6	18.3	78.3	-0.02	0.06	0.08
24C	145.4	23.7	172.5	3.63	1.82	1.56
25C	59.6	33.7	-45.7	-27.05	-31.13	-27.68
26C	-147.2	3.8	147.1	207.42	12.76	11.83
27C	33.2	30.1	-0.8	0.16	-0.08	-0.07
28C	-148.3	54.5	120.7	-0.03	0.05	-0.03
29C	-88.6	33.8	46.9	-0.02	0.04	-0.02
30C	-86.1	25.3	74.8	-0.00	0.03	-0.00
31C	-124.8	30.2	133.1	-0.02	0.02	-0.02
32C	-137.5	49.7	157.4	-0.00	0.02	-0.01
33C	-31.3	38.4	57.7	-0.01	0.03	-0.01
34C	-36.8	43.6	63.5	-0.02	0.02	-0.02
35C	145.4	19.8	172.6	0.07	-0.02	-0.02
36C	-111.8	11.0	114.0	-51.74	-39.66	-34.92
39C	-136.4	47.4	102.7	-0.01	0.03	-0.01
42C	-119.5	34.0	133.7	-0.01	0.01	-0.01
45C	-107.2	30.6	140.0	-0.01	0.02	-0.01
48H	-138.2	43.8	152.0	-0.51	0.75	-0.49
49H	-32.8	38.1	73.1	-0.39	0.69	-0.44
50H	-80.7	28.0	46.5	-1.00	3.29	-1.20
51H	-145.2	49.3	156.5	-0.19	0.31	-0.19
52H	-65.3	21.8	87.2	-0.17	0.08	-0.13
53H	-44.7	37.3	11.1	-0.11	0.14	-0.11

54H	172.1	26.9	177.6	9.28	-0.26	-0.92
55H	154.6	31.9	-169.0	2.65	-0.01	-0.10
56H	-130.6	6.1	102.1	2.40	-1.97	-1.62
57H	-121.5	4.6	123.6	-59.34	-21.73	-88.48
58H	34.5	28.5	-4.9	0.39	-0.20	-0.20
59H	35.6	41.9	-14.7	0.70	-0.37	-0.36
60H	36.5	20.6	-4.4	0.82	-0.44	-0.40
61H	55.3	39.9	-17.9	0.22	-0.10	-0.10
62H	-147.5	42.5	119.4	-0.08	0.17	-0.08
63H	-67.4	39.7	26.3	-0.08	0.15	-0.08
64H	146.8	53.4	-137.2	-0.09	-0.09	0.18
65H	-72.3	25.7	61.5	-0.04	0.07	-0.04
66H	-141.7	53.2	140.9	-0.07	0.06	-0.08
67H	-133.3	30.1	122.5	-0.07	0.07	-0.07
68H	-125.4	27.9	129.9	-0.03	0.08	-0.03
69H	-136.1	31.1	148.7	-0.04	0.09	-0.04
70H	-132.9	48.7	155.2	-0.04	0.08	-0.04
71H	-128.6	45.2	141.0	-0.03	0.07	-0.03
72H	-30.7	34.3	54.4	-0.05	0.10	-0.06
73H	-19.6	48.5	49.5	-0.06	0.12	-0.06
74H	-38.6	38.0	69.8	-0.04	0.08	-0.04
75H	-46.4	39.4	79.0	-0.05	0.11	-0.06
76H	154.8	16.2	162.6	0.17	-0.09	-0.09
77H	151.4	11.1	167.4	0.28	-0.17	-0.16
78H	142.3	27.0	-176.8	0.23	-0.16	-0.15
79H	23.7	19.4	-19.6	-5.10	5.04	-3.98
80H	14.9	41.5	24.6	0.09	-0.05	-0.05
81H	-37.0	52.9	42.6	-0.03	0.06	-0.03
82H	-92.9	36.2	130.2	-0.04	0.09	-0.04
83H	134.0	35.9	-151.8	132.49	129.14	142.90
84H	-13.6	7.3	49.7	34.02	34.58	46.85
3N	1.5	12.4	12.2	-0.01	0.03	0.07
8N	-134.6	3.4	144.2	0.07	0.22	0.17
14N	-78.8	40.8	62.8	0.03	0.10	0.04
20N	-71.7	15.2	70.7	-0.00	0.02	0.04

$A_{iso} = 1.1$ MHz

CoproB-β-1

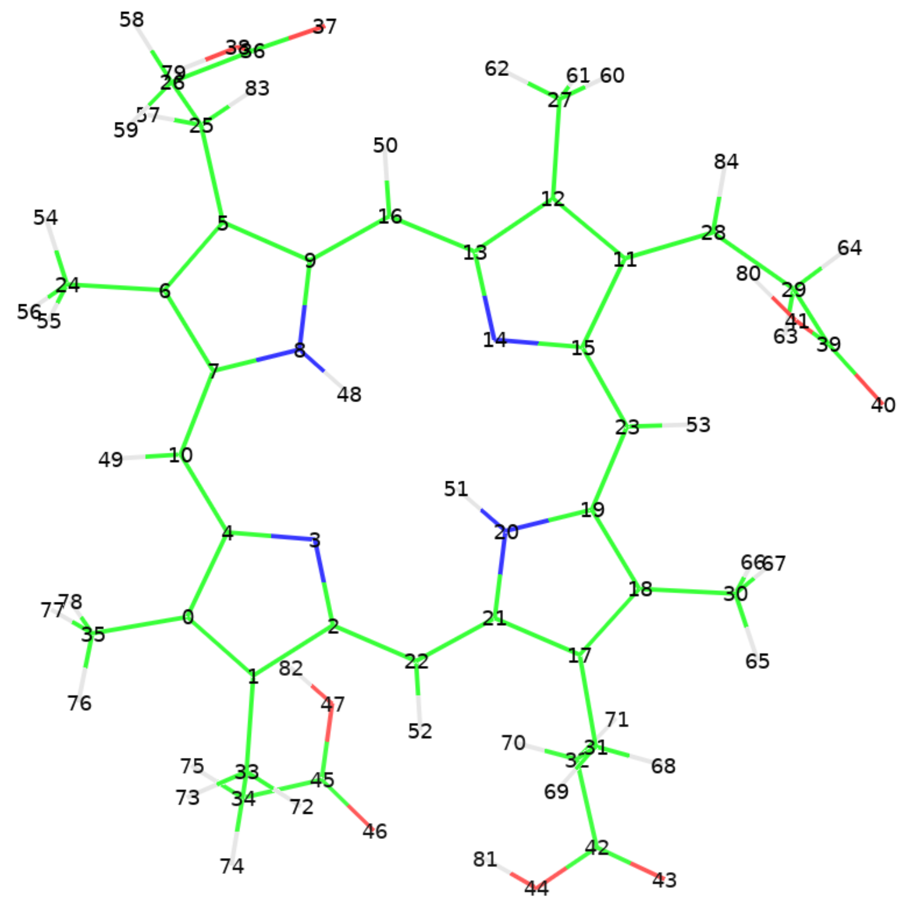

ELECTRONIC G-MATRIX

The g-matrix:

	2.0030935	-0.0000190	0.0000734
	0.0002394	2.0031445	0.0001425
	0.0000667	-0.0000421	2.0025253

gel	2.0023193	2.0023193	2.0023193		
gRMC	-0.0001589	-0.0001589	-0.0001589		
gDSO(tot)	0.0002910	0.0003322	0.0001268		
gPSO(tot)	0.0000632	0.0005143	0.0009545		
	----------	----------	----------		
g(tot)	2.0025146	2.0030069	2.0032417	iso=	2.0029211
Delta-g	0.0001953	0.0006877	0.0009224	iso=	0.0006018

Orientation:

X	-0.1086504	-0.7712968	-0.6271335
Y	-0.0600427	0.6348082	-0.7703333
Z	0.9922651	-0.0460423	-0.1152829

Euler rotation of hyperfine tensor to g-tensor

Atom	Alpha	Beta [degrees]	Gamma	Ax	Ay [MHz]	Az
0C	-119.1	15.5	117.2	4.01	-1.09	-0.86
1C	107.8	30.0	-105.7	-0.40	-1.89	-1.74
2C	-114.4	15.3	112.6	13.27	1.75	2.27
4C	108.5	19.7	-108.1	8.95	0.77	1.27
5C	100.5	32.8	-98.2	8.63	2.39	2.11
6C	-115.1	11.2	115.5	-8.62	-1.67	-1.53
7C	-103.4	24.5	103.6	5.91	-0.16	0.24
9C	-104.8	19.8	104.9	-16.87	-5.67	-4.81
10C	100.9	28.4	-100.0	-5.07	-1.99	-1.63
11C	96.0	41.5	-93.5	-52.94	-23.71	-27.92
12C	96.5	39.7	-94.2	108.95	7.00	5.96
13C	-105.0	16.3	104.9	-38.55	-20.14	-18.68
15C	102.1	19.5	-99.7	-10.15	-3.12	-3.49
16C	96.5	39.9	-94.3	26.40	2.88	2.72
17C	107.3	23.1	-99.6	-7.11	-2.35	-2.19
18C	-105.2	25.1	108.6	8.95	1.24	1.04
19C	100.7	36.5	-94.5	-6.37	-2.16	-1.58
21C	-111.2	20.5	114.3	13.31	1.88	1.71
22C	-103.2	38.3	100.8	-13.19	-3.12	-2.52
23C	-101.8	39.8	103.2	11.88	0.96	0.86
24C	-91.6	43.0	94.8	1.01	1.08	1.13
25C	31.1	43.1	-23.5	-1.03	-0.76	-0.98
26C	-25.4	44.0	65.0	1.65	2.02	1.77
27C	-83.1	40.8	77.6	-15.22	-14.32	-15.85
28C	-99.0	27.5	100.4	117.22	7.24	6.20
29C	72.8	3.3	-68.8	-18.43	-16.94	-18.99
30C	-66.6	23.7	66.7	-1.30	-1.08	-1.26
31C	122.6	46.1	-97.7	0.56	0.61	0.72
32C	-150.6	43.2	152.4	-1.09	-0.77	-0.83
33C	135.1	27.4	-103.5	-0.38	-0.41	-0.24
34C	45.3	37.8	-25.0	0.73	0.70	0.92
35C	-108.2	24.6	110.0	-0.87	-0.95	-0.75
36C	28.6	15.4	-1.8	-0.15	0.12	-0.11
39C	-68.0	17.5	110.9	45.82	38.92	39.21
42C	81.5	41.4	-61.8	-0.08	-0.07	-0.03
45C	13.5	42.7	-55.2	-0.06	-0.07	0.03
48H	95.7	37.3	-92.6	-3.12	-0.11	-1.99
49H	151.6	18.2	-109.7	0.50	0.51	1.70
50H	-104.7	32.5	105.8	-9.01	-4.00	-9.63
51H	101.1	22.1	-106.4	-0.63	-0.62	2.38
52H	-102.9	41.0	99.4	2.57	1.74	4.89
53H	107.4	32.0	-97.4	-4.92	-2.26	-3.65
54H	-110.4	36.6	96.8	-0.39	-0.38	0.01
55H	132.0	37.0	-138.8	-2.61	-2.28	-2.55
56H	81.2	30.1	-73.3	-3.57	-3.27	-3.45

57H	151.3	39.3	-162.3	0.92	1.73	0.94
58H	17.3	24.3	-4.1	-0.10	0.50	-0.08
59H	54.9	27.1	-18.7	-0.20	0.47	-0.29
60H	77.6	37.7	-71.4	26.21	28.48	32.98
61H	127.6	48.8	-121.5	50.04	51.09	56.46
62H	-51.0	30.4	50.7	2.96	9.06	3.53
63H	-63.0	10.0	76.7	33.46	40.09	34.02
64H	121.6	36.8	-117.6	3.01	9.96	2.35
65H	-78.3	19.0	78.9	-0.08	0.77	0.01
66H	-116.2	25.7	134.1	3.37	4.47	3.75
67H	-84.9	26.4	76.7	2.33	3.61	2.82
68H	83.1	42.6	-96.0	-0.67	-0.65	-0.19
69H	84.9	43.2	-100.9	-1.03	-1.04	-0.61
70H	89.8	34.2	-74.4	0.01	-0.01	0.48
71H	64.3	44.3	-69.0	-0.20	-0.02	0.37
72H	-55.6	53.2	26.0	0.02	0.61	0.05
73H	115.6	23.6	-129.5	0.09	0.13	0.62
74H	90.7	26.3	-82.9	-0.08	-0.09	0.20
75H	29.2	21.9	-36.3	-0.21	-0.18	0.19
76H	-154.5	12.3	126.6	-0.20	-0.22	0.37
77H	-87.7	42.2	116.9	2.06	2.15	2.74
78H	-115.7	35.8	75.2	2.41	2.48	3.08
79H	20.8	34.4	20.6	-0.19	0.36	-0.17
80H	34.0	1.7	-4.0	6.54	-6.48	-8.53
81H	109.7	37.6	-74.0	-0.07	-0.05	0.13
82H	176.2	39.8	161.1	0.34	-0.26	-0.14
83H	125.8	15.8	-131.8	-0.00	1.43	0.17
84H	94.6	33.2	-90.3	-34.18	-50.35	-14.76
3N	-176.4	6.8	175.5	-3.40	-0.27	-0.19
8N	-108.2	19.0	108.4	3.52	0.04	0.21
14N	99.5	31.5	-97.5	17.34	-0.50	-0.23
20N	103.0	31.3	-97.1	-1.13	-0.43	-0.32

$A_{iso} = 29.0$ MHz

CoproB-β-2

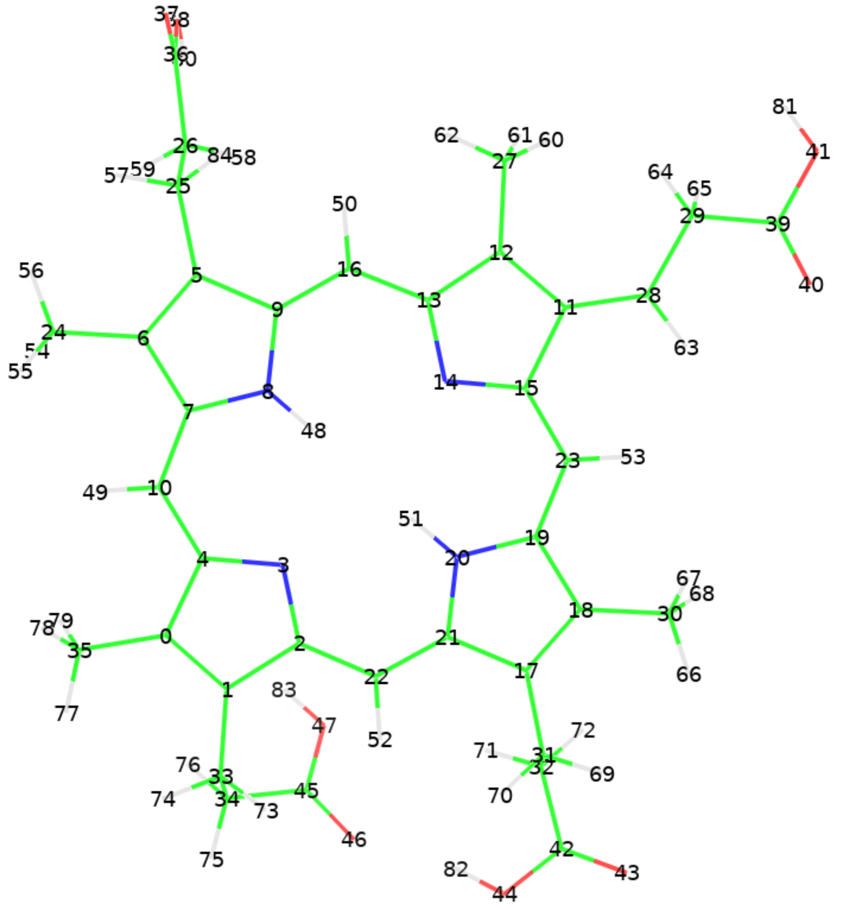

```
--------------------
ELECTRONIC G-MATRIX
--------------------

The g-matrix:
               2.0029655   -0.0000600   -0.0000310
              -0.0000202    2.0028995    0.0000745
               0.0000165    0.0000658    2.0023847

 gel           2.0023193    2.0023193    2.0023193
 gRMC         -0.0001533   -0.0001533   -0.0001533
 gDSO(tot)     0.0002147    0.0001430    0.0002382
 gPSO(tot)    -0.0000054    0.0005787    0.0005826
              ----------   ----------   ----------
 g(tot)        2.0023753    2.0028876    2.0029868 iso=  2.0027499
 Delta-g       0.0000560    0.0005683    0.0006675 iso=  0.0004306

Orientation:
  X            0.0031807    0.4631462   -0.8862762
  Y           -0.1324272    0.8786700    0.4586961
  Z            0.9911876    0.1159081    0.0641280
```

Euler rotation of hyperfine tensor to g-tensor

Atom	Alpha	Beta [degrees]	Gamma	Ax	Ay [MHz]	Az
0C	-83.4	30.2	84.0	4.03	-1.39	-1.64
1C	78.6	14.0	-80.2	0.53	-2.01	-2.19
2C	-81.9	32.0	83.7	15.66	2.52	1.91
4C	64.3	5.5	-63.5	13.13	1.87	1.25
5C	84.1	12.9	-81.8	11.20	2.38	2.64
6C	-87.8	19.3	90.4	-10.31	-2.24	-2.39
7C	-88.6	42.6	91.1	10.09	0.72	0.42
9C	-87.7	31.3	90.6	-18.68	-5.31	-6.24
10C	84.8	20.8	-83.6	-9.24	-2.44	-3.00
11C	93.5	18.5	-92.9	-57.83	-29.31	-23.97
12C	92.0	27.0	-90.4	108.42	7.08	8.16
13C	-89.4	33.4	90.8	-39.93	-18.63	-20.16
15C	89.3	7.0	-90.1	-11.44	-3.06	-2.70
16C	89.5	38.5	-86.0	30.25	3.38	3.40
17C	-138.3	2.1	138.1	-9.11	-2.64	-2.82
18C	-90.5	39.5	90.4	11.34	1.54	1.75
19C	89.3	12.1	-89.6	-10.87	-2.92	-3.70
21C	-90.5	42.8	89.8	14.63	1.61	1.73
22C	85.3	38.0	-88.1	-13.82	-3.67	-2.96
23C	-85.8	5.6	85.2	20.03	1.39	1.20
24C	90.0	26.7	-90.1	1.14	1.21	1.26
25C	103.9	4.3	-144.6	-1.41	-1.34	-1.13
26C	136.9	15.9	-103.1	2.07	1.59	1.76
27C	85.8	33.5	-91.3	-14.79	-14.41	-15.83
28C	-92.3	29.9	91.5	121.81	5.41	7.18
29C	-130.5	9.3	130.1	-18.44	-17.39	-19.23
30C	-92.9	31.6	90.9	-1.62	-1.59	-1.38
31C	107.8	32.7	-115.1	0.75	0.94	0.85
32C	-84.3	35.0	62.5	-1.67	-1.32	-1.29
33C	139.6	19.5	-141.1	-0.60	-0.44	-0.64
34C	16.5	13.8	-61.5	1.40	1.13	1.10
35C	-91.1	38.8	90.7	-0.98	-0.85	-1.08
36C	-39.4	18.6	66.3	0.17	0.19	0.35
39C	108.1	41.7	-77.7	3.88	3.50	5.50
42C	138.2	36.3	-109.7	-0.19	-0.12	-0.15
45C	139.5	50.8	-123.0	-0.10	0.01	-0.08
48H	87.7	18.8	-85.4	-3.02	-1.86	-0.14
49H	101.3	17.0	-101.4	1.36	3.15	1.13
50H	92.9	42.6	-87.3	-9.59	-4.23	-10.97
51H	103.5	11.7	-103.6	-1.20	1.33	-0.67
52H	84.5	37.5	-87.5	2.51	1.77	4.98
53H	91.5	28.7	-91.4	-7.07	-5.72	-3.43
54H	57.3	16.5	-69.1	-3.71	-3.61	-3.37
55H	116.3	14.8	-100.9	-3.22	-3.16	-2.87

56H	83.7	38.8	-87.4	-0.39	-0.36	0.05
57H	-153.4	4.7	180.0	0.86	0.89	1.74
58H	-45.8	24.6	1.6	-0.70	-0.44	0.66
59H	-9.6	31.8	-13.6	-0.32	-0.37	0.41
60H	105.0	17.2	-87.4	27.36	33.66	29.49
61H	116.9	26.1	-141.8	53.32	59.36	54.39
62H	-77.2	36.2	99.2	3.02	3.58	8.72
63H	-91.4	25.5	90.4	-36.27	-55.15	-14.81
64H	99.7	10.5	-114.3	28.49	35.52	30.53
65H	-179.9	28.5	-160.7	59.76	66.76	60.61
66H	-88.4	29.0	89.6	-0.14	-0.08	0.77
67H	-120.5	34.0	111.0	3.71	4.16	4.98
68H	-61.2	34.0	69.8	3.49	3.95	4.78
69H	55.5	33.6	-54.6	-1.10	-0.60	-1.08
70H	128.7	44.1	-143.9	-1.02	-0.57	-1.00
71H	139.9	47.0	-134.7	-0.00	0.52	0.10
72H	63.6	33.9	-40.5	-0.16	0.48	0.04
73H	39.6	53.7	-37.6	0.26	0.98	0.28
74H	7.3	17.8	-14.2	0.10	0.76	0.15
75H	35.8	22.5	-23.8	-0.02	0.32	-0.04
76H	3.2	41.9	26.9	-0.27	0.23	-0.23
77H	-145.5	49.3	156.3	-0.26	0.42	-0.24
78H	133.1	35.5	-108.1	2.25	2.35	3.06
79H	26.1	41.5	-63.6	2.92	3.00	3.72
80H	-17.0	31.5	15.8	-0.16	-0.16	0.28
81H	-144.0	29.3	140.4	-0.86	1.39	-0.66
82H	127.3	34.8	-120.3	-0.07	0.14	-0.06
83H	-54.2	32.3	20.6	0.33	-0.11	-0.27
84H	-153.4	8.8	160.4	0.76	1.05	2.22
3N	-78.1	15.4	78.6	-4.28	-0.27	-0.35
8N	-88.6	31.4	90.4	3.26	0.14	-0.01
14N	89.2	20.5	-87.6	14.27	-0.08	-0.32
20N	119.7	10.6	-115.4	-0.08	-0.23	-0.34

$A_{\mathrm{iso}} = 30.3$ MHz

CoproB-α-1

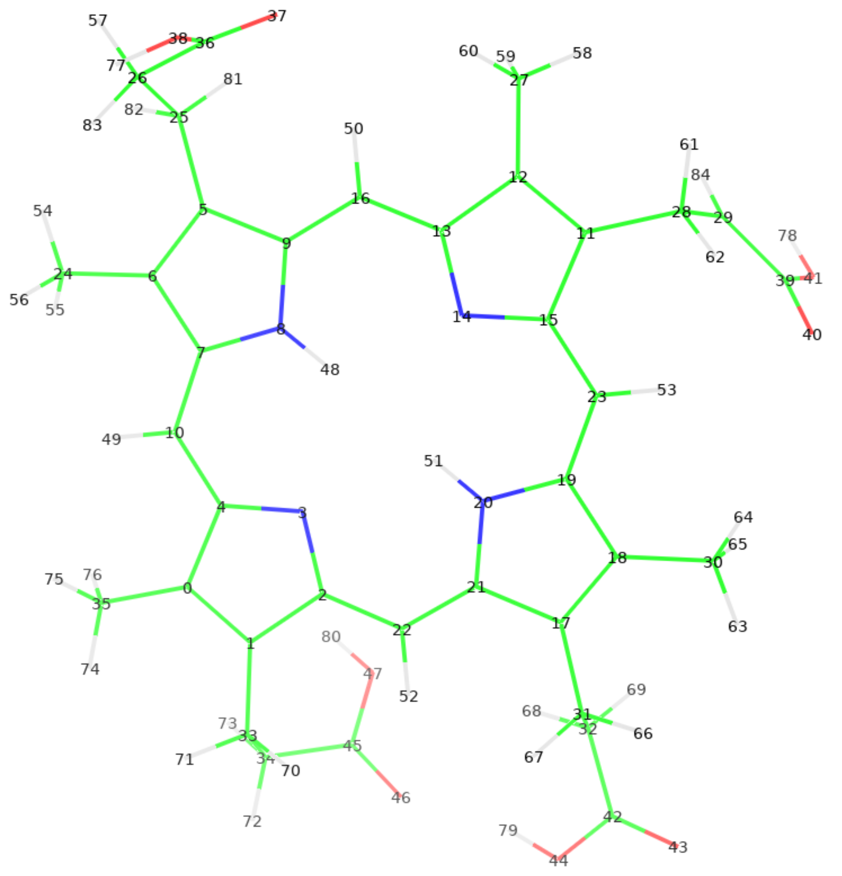

```
-------------------
ELECTRONIC G-MATRIX
-------------------

The g-matrix:
               2.0032540   -0.0010556   -0.0002426
              -0.0010781    2.0037721   -0.0005830
              -0.0002661   -0.0004703    2.0039552

 gel           2.0023193    2.0023193    2.0023193
 gRMC         -0.0001793   -0.0001793   -0.0001793
 gDSO(tot)     0.0001213    0.0003808    0.0004037
 gPSO(tot)    -0.0000107    0.0014960    0.0021705
              ----------   ----------   ----------
 g(tot)        2.0022506    2.0040167    2.0047141 iso=  2.0036605
 Delta-g      -0.0000687    0.0016974    0.0023948 iso=  0.0013412

Orientation:
  X            0.7296553   -0.4614592    0.5046370
  Y            0.6150389    0.1203170   -0.7792631
  Z            0.2988817    0.8789649    0.3716053
```

Euler rotation of hyperfine tensor to g-tensor

Atom	Alpha	Beta	Gamma	Ax	Ay	Az
		[degrees]			[MHz]	
0C	71.1	17.5	-70.8	-0.06	-0.00	-0.12
1C	114.6	19.2	-110.2	-0.02	0.12	-0.08
2C	95.5	17.6	-91.2	0.13	0.51	0.03
4C	92.4	18.7	-85.9	0.15	0.75	0.07
5C	32.6	46.5	-12.0	0.10	0.40	-0.00
6C	36.4	39.5	-24.3	-0.07	-0.28	-0.15
7C	33.0	33.7	-23.9	0.14	0.75	0.05
9C	37.4	25.1	-0.6	0.16	-0.18	-0.08
10C	88.3	19.2	-79.7	-0.14	-0.78	-0.18
11C	61.3	34.3	-23.5	55.28	41.31	42.10
12C	133.2	45.6	-125.9	-0.99	2.82	0.15
13C	49.3	51.5	-53.3	2.68	1.66	1.88
15C	-134.8	10.9	121.9	-0.08	-0.39	-1.42
16C	-171.7	43.0	160.5	-0.06	0.59	0.15
17C	144.6	34.6	-136.1	0.02	-0.46	-0.16
18C	116.6	37.7	-141.5	0.25	0.42	-0.02
19C	144.5	28.9	-129.2	0.08	-0.88	-0.40
21C	129.1	26.4	-127.1	0.12	0.43	-0.04
22C	118.0	19.7	-107.2	-0.05	-0.36	-0.16
23C	93.2	27.9	-117.9	-0.10	0.83	-0.67
24C	37.4	41.0	-19.6	0.06	0.00	0.00
25C	120.4	46.3	-138.7	-0.09	-0.09	-0.01
26C	-72.9	39.2	42.0	0.11	0.17	0.09
27C	76.1	37.9	-100.7	-0.61	-0.91	-0.15
28C	-152.9	2.7	167.3	-22.78	-24.32	-27.26
29C	-93.6	15.6	91.1	198.52	8.60	9.88
30C	10.8	43.0	12.2	-0.08	-0.10	0.38
31C	151.0	43.1	-136.2	0.12	-0.00	0.01
32C	150.3	42.1	-144.4	-0.00	-0.11	-0.09
33C	-126.0	27.3	132.8	-0.01	-0.05	-0.05
34C	-164.9	14.0	156.2	0.09	0.05	0.04
35C	76.5	18.8	-59.9	0.00	-0.03	-0.04
36C	-107.2	40.0	84.5	-0.03	0.06	-0.04
39C	91.7	16.9	-96.0	-46.76	-40.10	-35.01
42C	137.2	55.9	-130.9	0.04	-0.03	-0.03
45C	-113.1	43.1	104.6	0.03	-0.02	-0.02
48H	47.2	16.8	-25.6	0.61	-0.33	-0.28
49H	123.4	31.7	-110.0	0.27	0.08	0.17
50H	136.9	43.8	-128.7	-0.47	-0.55	0.57
51H	121.8	17.4	-100.2	0.67	-0.47	-0.35
52H	176.8	16.1	-164.7	0.30	-0.07	-0.06
53H	169.2	32.7	-131.2	4.11	-2.95	-1.28
54H	37.2	45.3	-17.9	0.12	-0.08	-0.07
55H	-32.8	34.2	58.7	0.11	-0.08	-0.09
56H	13.8	25.5	17.3	0.09	-0.10	-0.10

57H	124.8	45.9	-125.5	-0.06	-0.06	0.16
58H	91.8	38.5	-85.8	-0.38	-0.75	3.31
59H	75.0	23.8	-113.5	1.96	2.18	4.43
60H	117.6	44.5	-101.9	0.37	0.20	2.11
61H	-154.0	42.7	176.3	75.42	86.92	74.07
62H	87.8	26.4	-88.7	-2.30	9.10	-1.69
63H	29.6	36.6	2.7	-0.23	-0.28	0.80
64H	21.4	42.7	-2.4	-0.84	-1.01	2.05
65H	5.9	36.5	32.4	-0.31	-0.38	1.44
66H	164.8	46.5	-151.2	0.28	-0.20	-0.19
67H	156.1	40.3	-138.7	0.17	-0.17	-0.16
68H	-127.7	49.5	109.9	0.26	-0.10	-0.12
69H	152.4	40.2	-159.5	0.35	-0.17	-0.15
70H	145.1	26.6	-129.3	0.15	-0.04	-0.03
71H	135.3	18.9	-119.8	0.13	-0.02	-0.02
72H	-113.8	43.5	110.4	0.09	-0.04	-0.04
73H	-174.3	6.7	173.9	0.09	-0.05	-0.05
74H	90.4	36.6	-76.0	0.09	-0.05	-0.05
75H	-77.2	42.7	103.2	0.17	0.02	0.02
76H	-68.2	30.1	82.0	0.20	0.04	0.05
77H	-86.8	29.0	60.7	-0.08	0.16	-0.08
78H	102.4	5.3	-105.2	-6.45	3.95	-4.21
79H	129.1	51.3	-124.9	0.13	-0.07	-0.06
80H	-124.7	10.3	119.9	0.16	-0.07	-0.09
81H	153.6	41.1	-139.2	-0.10	-0.12	0.33
82H	169.4	44.0	-145.0	-0.00	-0.01	0.27
83H	141.8	57.8	-127.6	-0.09	-0.09	0.16
84H	-92.0	23.4	88.9	-56.33	-86.36	-19.43
3N	88.3	17.5	-79.5	0.01	-0.18	-0.02
8N	61.8	44.3	-34.6	0.04	-0.05	-0.02
14N	-20.2	20.2	-21.4	0.59	-0.06	-0.19
20N	99.1	19.9	-131.6	0.03	0.07	-0.03

CoproB-α-2

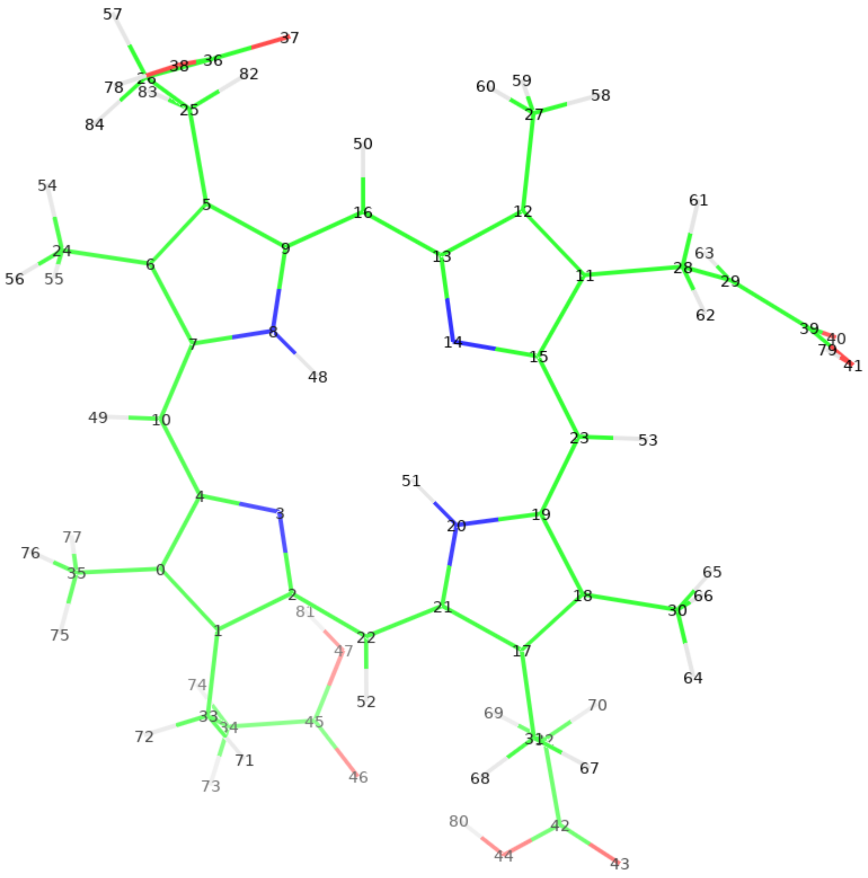


```
-------------------
ELECTRONIC G-MATRIX
-------------------

The g-matrix:
              2.0040333   -0.0010432   -0.0006241
             -0.0011144    2.0028743    0.0005572
             -0.0002150    0.0001319    2.0044930

 gel          2.0023193    2.0023193    2.0023193
 gRMC        -0.0001797   -0.0001797   -0.0001797
 gDSO(tot)    0.0001278    0.0004132    0.0003046
 gPSO(tot)   -0.0000411    0.0014902    0.0026873
             ----------   ----------   ----------
 g(tot)       2.0022262    2.0040430    2.0051315 iso=  2.0038002
 Delta-g     -0.0000931    0.0017237    0.0028122 iso=  0.0014809

Orientation:
  X           0.5058158   -0.5709435   -0.6466637
  Y           0.8618303    0.3019608    0.4075147
  Z          -0.0374008   -0.7634417    0.6447929
```

Euler rotation of hyperfine tensor to g-tensor

Atom	Alpha	Beta	Gamma	Ax	Ay	Az
		[degrees]			[MHz]	
0C	-111.4	48.7	127.7	-0.03	-0.17	-0.08
1C	95.8	42.1	-73.2	0.06	-0.00	0.17
2C	108.4	43.8	-86.7	-0.04	-0.11	-0.18
4C	-110.6	50.6	135.6	0.04	0.30	-0.03
5C	-57.2	44.5	52.8	-0.11	-0.00	-0.02
6C	51.3	44.9	-78.8	-0.08	-0.00	0.16
7C	39.9	40.5	-72.0	0.00	0.07	0.40
9C	44.0	37.7	-63.5	0.04	0.20	0.56
10C	-118.0	52.3	139.4	-0.07	-0.30	-0.11
11C	-65.8	41.1	27.8	21.94	22.42	29.62
12C	115.3	50.2	-140.5	-1.08	-0.13	-1.19
13C	-47.0	26.0	23.8	1.10	1.89	1.66
15C	31.3	40.8	4.1	0.18	-0.89	0.36
16C	-59.7	44.4	57.2	-0.27	-0.63	0.04
17C	-86.5	43.8	82.7	0.12	-0.16	-0.07
18C	-94.4	40.1	75.3	0.28	-0.06	-0.10
19C	-92.5	40.9	92.5	0.56	-0.33	-0.02
21C	-90.4	35.6	98.2	0.02	-0.29	-0.17
22C	84.7	42.1	-73.1	0.08	-0.03	0.57
23C	29.1	47.8	-18.6	1.66	1.31	3.86
24C	-50.7	51.6	50.2	-0.06	-0.05	-0.00
25C	-75.8	41.0	66.3	-0.05	-0.05	0.03
26C	-101.4	28.5	63.8	0.02	0.02	0.09
27C	-43.2	40.0	77.6	0.20	-0.14	0.65
28C	46.3	19.5	-44.2	-26.35	-30.16	-26.81
29C	-55.8	11.8	57.7	201.96	10.66	9.84
30C	-60.3	19.4	34.7	0.26	-0.15	-0.14
31C	-81.4	41.1	72.6	0.09	-0.03	-0.02
32C	68.3	35.2	-76.6	0.03	-0.07	-0.08
33C	-167.0	9.1	176.4	0.00	-0.04	-0.04
34C	76.6	14.0	-61.7	0.06	0.02	0.03
35C	123.6	39.3	-96.0	0.04	-0.00	0.00
36C	-40.8	25.2	66.2	-0.03	-0.03	0.05
39C	-76.6	18.0	76.7	-52.27	-41.28	-36.40
42C	-41.7	23.8	36.6	0.04	-0.03	-0.03
45C	-62.6	8.3	76.2	0.03	-0.02	-0.02
48H	-137.4	42.8	155.3	0.57	-0.23	-0.20
49H	-127.9	55.8	146.6	0.16	-0.03	0.03
50H	-95.7	38.9	65.2	-0.23	-0.21	0.81
51H	125.2	42.2	-104.4	0.56	-0.39	-0.53
52H	79.7	25.9	-87.8	0.11	-0.34	-0.28
53H	122.8	25.9	-142.5	7.03	-1.17	-2.00
54H	-62.2	48.7	56.1	-0.05	-0.06	0.13
55H	-153.9	35.6	-163.7	0.23	0.03	0.04

56H	171.8	43.0	-128.8	0.27	0.08	0.08
57H	-63.6	33.1	71.6	-0.06	-0.06	0.14
58H	-78.7	31.4	116.8	-0.68	-0.96	3.22
59H	66.6	51.7	-44.8	0.19	2.33	0.31
60H	-80.1	22.6	92.7	-0.82	-0.88	0.83
61H	143.6	20.0	-155.3	120.80	117.97	131.40
62H	-105.5	48.5	128.8	14.05	25.11	13.35
63H	-54.2	12.4	57.8	-58.83	-21.34	-87.02
64H	-54.2	22.5	27.7	0.64	-0.34	-0.30
65H	-37.7	23.6	15.5	1.55	-0.85	-0.69
66H	-79.2	18.3	41.7	1.22	-0.58	-0.52
67H	-72.0	27.0	54.6	0.30	-0.17	-0.16
68H	-86.9	41.7	75.6	0.19	-0.14	-0.12
69H	-46.9	17.9	50.5	0.25	-0.13	-0.10
70H	-38.7	25.4	37.1	0.36	-0.18	-0.16
71H	-102.4	40.0	102.8	0.15	-0.05	-0.04
72H	-110.4	37.2	115.8	0.13	-0.02	-0.02
73H	-100.2	5.0	111.1	0.09	-0.04	-0.04
74H	-94.4	40.3	113.5	0.09	-0.05	-0.05
75H	-118.2	40.0	131.9	0.08	-0.05	-0.04
76H	-146.9	31.1	164.9	0.07	-0.07	-0.07
77H	145.6	21.8	-115.0	0.08	-0.07	-0.07
78H	-17.5	21.1	47.8	-0.07	-0.07	0.14
79H	-8.5	16.6	7.1	-5.46	4.79	-3.93
80H	-42.6	19.3	45.0	0.13	-0.07	-0.06
81H	87.0	21.0	-64.0	0.16	-0.09	-0.08
82H	-78.0	42.6	73.0	-0.11	-0.13	0.26
83H	-81.3	46.0	68.1	-0.04	-0.06	0.19
84H	-32.8	31.7	53.0	-0.07	-0.08	0.14
3N	112.4	43.1	-82.5	0.01	-0.02	0.05
8N	-51.9	56.5	42.5	-0.03	-0.14	0.02
14N	23.9	41.3	-65.2	-0.11	0.19	-0.25
20N	-106.0	43.4	109.5	0.15	0.28	0.06

CoproA-β-1-tautomer

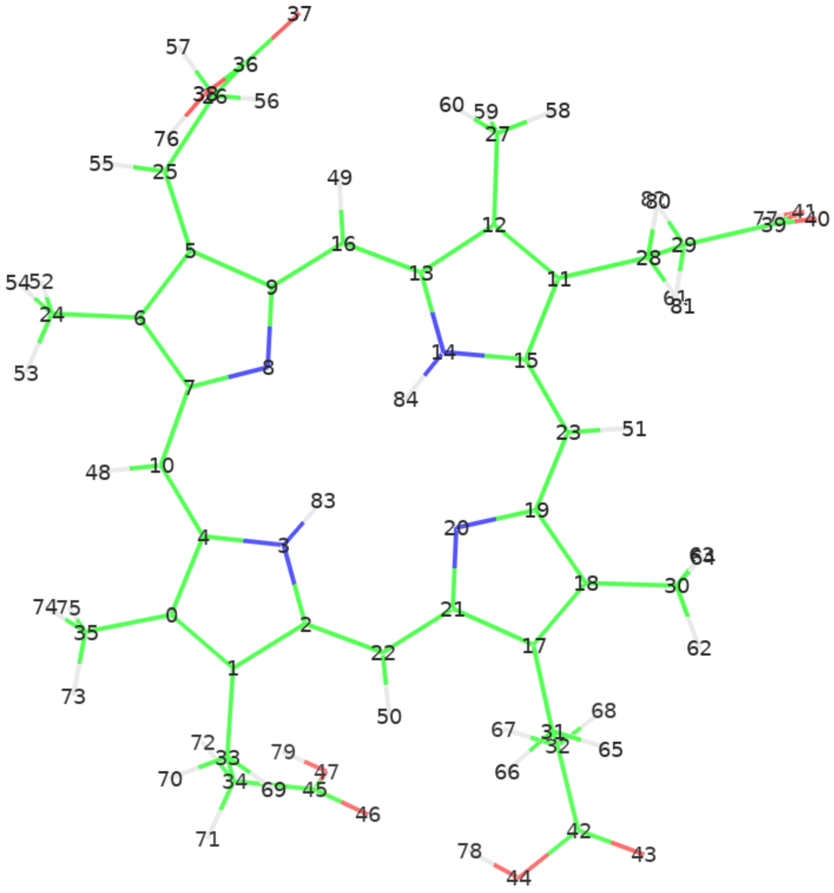


```
--------------------
ELECTRONIC G-MATRIX
--------------------

The g-matrix:
              2.0031519   -0.0002637   -0.0000486
              0.0000880    2.0030584    0.0001132
              0.0001500    0.0000851    2.0025607

 gel          2.0023193    2.0023193    2.0023193
 gRMC        -0.0001572   -0.0001572   -0.0001572
 gDSO(tot)    0.0003175    0.0003603    0.0001187
 gPSO(tot)    0.0000548    0.0005094    0.0009240
             ----------   ----------   ----------
 g(tot)       2.0025345    2.0030318    2.0032048 iso=  2.0029237
 Delta-g      0.0002152    0.0007125    0.0008855 iso=  0.0006044

Orientation:
  X          -0.1087143    0.5098087   -0.8533911
  Y          -0.2024121    0.8291417    0.5211078
  Z           0.9732474    0.2293886    0.0130519
```

Euler rotation of hyperfine tensor to g-tensor

Atom	Alpha	Beta	Gamma	Ax	Ay	Az
		[degrees]			[MHz]	
0C	80.5	21.7	-87.9	10.08	2.29	2.02
1C	-78.0	21.5	71.8	-8.90	-2.18	-2.03
2C	-83.3	41.0	79.1	8.85	0.51	0.81
4C	-82.8	30.5	77.6	-16.39	-5.52	-4.67
5C	83.3	33.6	-89.9	-52.22	-23.62	-27.77
6C	82.1	28.5	-88.2	105.78	6.87	5.84
7C	-81.2	25.8	76.6	-37.68	-19.63	-18.27
9C	72.7	12.8	-77.8	-8.77	-3.03	-3.37
10C	76.6	15.9	-83.1	26.41	2.91	2.76
11C	73.6	18.2	-79.4	-6.72	-2.46	-2.29
12C	-80.3	32.1	77.6	9.07	1.05	0.85
13C	80.4	30.6	-86.9	-4.93	-1.98	-1.39
15C	-77.0	22.2	73.2	13.92	2.05	1.86
16C	79.9	37.8	-87.8	10.49	0.54	0.71
17C	-82.9	27.2	80.7	2.24	-1.21	-0.97
18C	70.0	12.5	-74.1	-0.19	-1.75	-1.58
19C	-78.8	23.0	75.8	14.19	1.82	2.35
21C	64.0	8.0	-69.6	10.78	1.42	1.92
22C	78.8	25.2	-86.7	-8.61	-2.37	-1.94
23C	82.2	42.3	-87.8	-13.97	-2.64	-3.27
24C	84.2	41.9	-90.6	-15.26	-15.56	-14.06
25C	-83.0	36.7	77.2	118.57	7.06	6.03
26C	-115.1	6.1	106.4	-18.66	-17.11	-19.23
27C	-99.3	28.0	100.2	-1.37	-1.16	-1.33
28C	61.4	37.5	-84.5	0.46	0.52	0.64
29C	-56.5	46.9	64.0	-1.04	-0.80	-0.85
30C	64.1	17.0	-68.6	-0.30	-0.41	-0.24
31C	-85.4	32.4	82.1	-0.59	-0.65	-0.48
32C	-131.1	56.8	145.2	0.96	0.91	1.25
33C	72.0	45.1	-83.0	0.88	1.02	0.96
34C	52.4	34.9	-88.6	-2.27	-1.92	-1.95
35C	43.1	12.0	-49.8	-1.40	-1.10	-1.33
36C	-24.8	31.3	66.4	39.04	45.98	39.35
39C	159.3	26.7	-120.5	-0.13	-0.14	-0.08
42C	-82.1	36.8	71.5	0.11	0.10	0.19
45C	64.7	49.0	-43.1	0.06	0.14	0.07
48H	-84.3	38.9	79.8	-8.79	-3.78	-9.63
49H	65.2	18.4	-75.3	-4.62	-2.11	-3.12
50H	68.1	23.0	-85.5	1.44	1.05	3.04
51H	79.8	39.1	-84.7	2.73	5.18	1.83
52H	52.9	37.8	-60.4	37.78	39.61	44.42
53H	-81.6	32.3	77.2	-0.75	4.97	-0.57
54H	116.2	33.7	-121.5	37.82	39.66	44.42
55H	81.0	24.7	-89.8	-34.51	-51.26	-15.06
56H	-110.9	18.9	92.8	34.19	40.90	34.78

A_{iso} = 27.5 MHz

57H	48.5	29.9	-58.5	3.15	10.19	2.47
58H	-99.4	26.4	100.2	0.02	0.90	0.13
59H	-74.5	30.0	55.1	3.74	4.82	4.14
60H	-90.5	35.2	99.0	2.11	3.43	2.66
61H	76.0	32.0	-114.9	-0.08	-0.04	0.40
62H	59.0	6.2	-44.1	-0.25	-0.22	0.22
63H	148.4	30.1	-115.3	1.32	1.23	1.79
64H	104.8	36.0	-67.7	0.81	0.89	1.37
65H	-96.7	22.3	64.3	0.15	0.17	0.68
66H	84.8	37.2	-99.1	0.44	1.02	0.48
67H	56.7	40.8	-21.3	-0.29	0.19	-0.11
68H	171.9	31.4	145.2	-0.27	-0.22	0.15
69H	40.5	42.9	-43.0	-0.93	-0.58	-0.91
70H	107.3	45.4	-128.1	-1.23	-0.81	-1.24
71H	-63.4	43.4	86.1	-0.15	-0.12	0.05
72H	124.6	46.6	-125.7	-0.21	0.41	0.04
73H	10.2	32.7	-16.8	-0.01	0.79	-0.03
74H	152.8	4.9	-169.9	2.15	3.47	2.39
75H	16.2	12.7	-10.4	2.85	4.04	3.07
76H	-112.9	11.3	77.7	6.79	-6.73	-8.83
77H	112.0	39.0	-121.6	-0.08	-0.05	0.15
78H	-85.9	44.6	97.1	-0.09	-0.05	0.12
79H	98.4	34.6	-77.4	-0.11	0.19	-0.07
80H	68.4	36.3	-80.4	-0.96	-0.94	-0.45
81H	93.4	31.7	-78.4	-0.61	-0.65	-0.14
82H	131.8	39.2	-114.9	-0.19	-0.07	0.39
83H	85.6	38.5	-92.5	-2.58	0.20	-1.46
84H	67.7	11.9	-72.5	-0.43	-0.52	2.62
3N	-81.7	29.3	76.3	2.53	-0.03	0.12
8N	80.5	23.9	-86.6	16.96	-0.53	-0.27
14N	76.6	24.5	-82.9	-1.53	-0.47	-0.37
20N	-61.6	8.1	57.5	-3.61	-0.29	-0.21

CoproA-β-2-tautomer

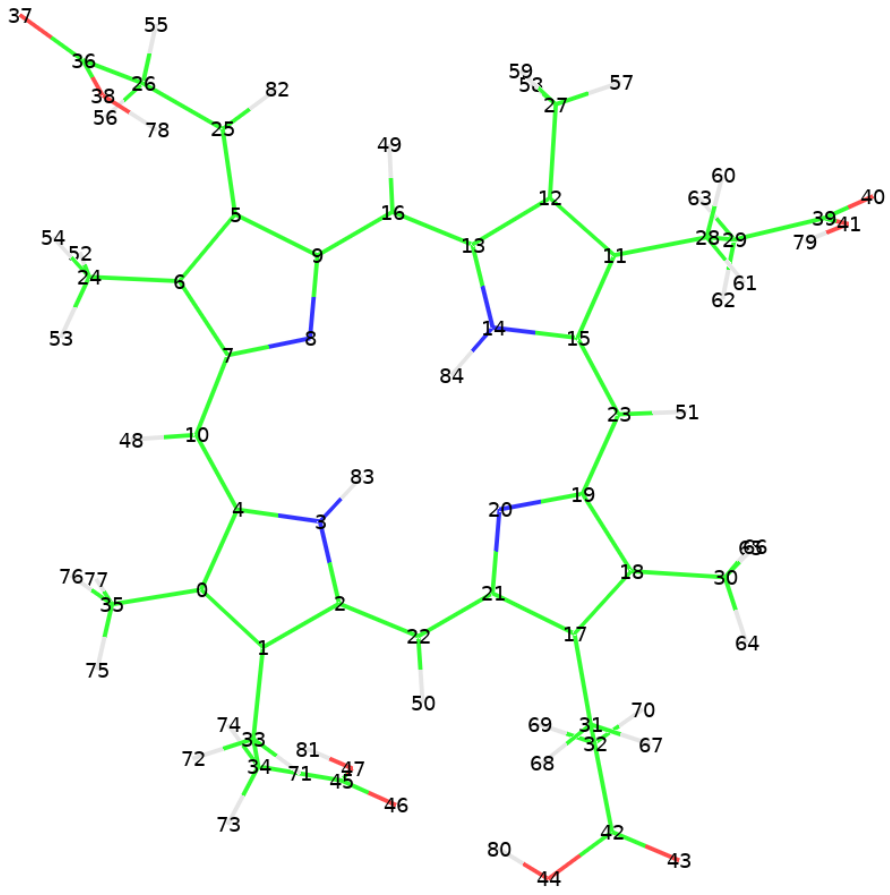


```
-------------------
ELECTRONIC G-MATRIX
-------------------

The g-matrix:
              2.0030162    0.0003378    0.0001439
              0.0003405    2.0031504    0.0002658
              0.0003529    0.0000875    2.0024807

 gel          2.0023193    2.0023193    2.0023193
 gRMC        -0.0001588   -0.0001588   -0.0001588
 gDSO(tot)    0.0002570    0.0001368    0.0002618
 gPSO(tot)   -0.0000371    0.0004563    0.0010912
             ----------   ----------   ----------
 g(tot)       2.0023804    2.0027535    2.0035135 iso=  2.0028825
 Delta-g      0.0000611    0.0004342    0.0011942 iso=  0.0005632

Orientation:
  X          -0.3300677    0.7013393    0.6318057
  Y          -0.0705574   -0.6857740    0.7243864
  Z           0.9413166    0.1945180    0.2758365
```

Euler rotation of hyperfine tensor to g-tensor

Atom	Alpha	Beta	Gamma	Ax	Ay	Az
		[degrees]			[MHz]	
0C	68.9	28.2	-81.2	11.39	2.09	2.35
1C	-51.1	15.4	42.9	-9.59	-2.39	-2.54
2C	-76.1	37.9	69.6	10.91	1.08	0.82
4C	-68.4	24.2	59.7	-16.70	-4.88	-5.76
5C	78.7	32.0	-92.2	-53.84	-28.97	-23.83
6C	78.8	36.2	-91.6	105.07	6.41	7.36
7C	-71.8	22.0	62.1	-37.76	-18.14	-19.50
9C	71.0	24.0	-82.8	-9.20	-3.19	-2.89
10C	66.5	19.4	-78.9	27.75	2.81	2.92
11C	56.4	22.7	-70.6	-7.76	-2.51	-2.67
12C	-65.2	28.8	54.7	10.22	1.18	1.38
13C	69.9	32.5	-84.5	-7.88	-2.17	-2.87
15C	-57.1	20.6	47.1	14.50	1.82	2.00
16C	74.2	30.3	-88.3	15.30	0.89	0.64
17C	-69.8	22.4	59.5	1.99	-1.24	-1.48
18C	66.7	21.0	-78.7	0.39	-1.66	-1.86
19C	-62.0	18.5	52.7	15.23	2.52	1.94
21C	59.5	17.6	-71.5	12.80	2.24	1.69
22C	73.8	36.5	-86.0	-10.68	-2.31	-2.84
23C	-76.1	41.2	68.7	-14.51	-2.83	-3.50
24C	-103.1	43.2	96.3	-14.18	-15.30	-13.84
25C	-69.8	15.9	59.6	119.72	5.73	7.45
26C	77.0	11.8	-83.7	-17.64	-16.59	-18.39
27C	-60.0	20.0	55.1	-1.52	-1.49	-1.29
28C	-62.7	46.6	72.6	0.57	0.64	0.75
29C	-68.9	24.6	82.3	-1.26	-0.99	-0.95
30C	71.4	24.9	-80.8	-0.41	-0.35	-0.53
31C	-76.7	26.3	66.8	-0.61	-0.49	-0.68
32C	-36.2	33.1	70.1	1.33	1.02	0.97
33C	-80.0	40.6	81.6	0.91	1.06	1.00
34C	-49.2	48.0	73.2	-2.35	-2.00	-2.03
35C	62.9	15.9	-67.4	-1.60	-1.53	-1.30
36C	-160.9	4.3	-173.4	52.71	44.84	45.12
39C	122.2	29.7	-158.2	-0.13	-0.08	-0.14
42C	-60.0	32.9	55.4	0.11	0.20	0.11
45C	128.8	50.4	-113.6	0.05	0.06	0.14
48H	-70.1	33.3	64.8	-9.08	-10.23	-3.91
49H	76.3	32.6	-90.4	-5.94	-3.94	-2.88
50H	64.2	37.6	-74.2	1.88	3.80	1.32
51H	-77.5	43.7	70.4	2.80	5.35	1.88
52H	74.8	37.3	-59.0	49.44	55.53	50.66
53H	-84.4	23.6	58.4	0.94	1.32	6.46
54H	68.5	31.3	-98.9	31.30	37.57	33.33
55H	-129.6	16.5	129.3	12.59	19.77	12.93

$A_{iso} = 29.6$ MHz

56H	76.8	24.6	-77.0	18.03	24.71	19.97
57H	-64.3	19.1	50.2	-0.12	-0.06	0.77
58H	-29.7	28.7	30.6	3.73	4.16	4.94
59H	-102.3	20.8	82.7	2.91	3.38	4.18
60H	96.0	41.9	-114.2	-0.91	-0.40	-0.90
61H	105.3	42.6	-124.5	-0.87	-0.42	-0.90
62H	122.8	39.9	-165.5	-0.00	0.46	-0.04
63H	82.8	37.6	-124.1	-0.21	0.41	-0.04
64H	142.9	19.0	-156.3	-0.27	0.24	-0.25
65H	120.2	39.2	-160.9	1.60	2.13	1.51
66H	125.9	54.7	-143.2	1.13	1.76	1.23
67H	-22.9	36.2	15.0	0.16	0.73	0.19
68H	59.1	46.2	-83.3	0.42	0.44	1.04
69H	152.0	44.4	-135.8	-0.30	-0.11	0.21
70H	68.9	41.1	-23.9	0.17	-0.29	-0.24
71H	109.2	37.9	-84.0	-0.94	-0.93	-0.56
72H	-129.0	53.2	145.0	-1.34	-0.88	-1.33
73H	-116.0	41.7	113.2	-0.14	0.06	-0.11
74H	-71.3	49.3	38.6	-0.22	0.42	0.03
75H	85.2	15.3	-60.2	-0.01	-0.03	0.84
76H	16.3	21.1	-35.6	2.61	2.92	3.97
77H	125.6	9.1	-127.9	3.43	3.70	4.64
78H	-103.8	34.8	109.8	6.23	-6.35	-7.04
79H	-54.3	56.4	44.3	-0.09	-0.05	0.15
80H	-99.7	39.6	83.7	-0.09	0.13	-0.05
81H	109.5	44.7	-128.6	-0.11	-0.07	0.19
82H	-71.8	14.9	62.1	-34.86	-53.72	-14.02
83H	-76.1	45.0	66.5	-2.45	0.23	-1.31
84H	63.9	22.1	-75.4	-0.73	2.03	-0.56
3N	-65.3	23.1	57.5	2.26	0.07	-0.07
8N	77.9	32.8	-91.9	15.09	-0.22	-0.45
14N	68.3	28.5	-84.4	-0.93	-0.30	-0.40
20N	4.0	8.3	-15.0	-4.06	-0.24	-0.32

CoproB-β-1-tautomer

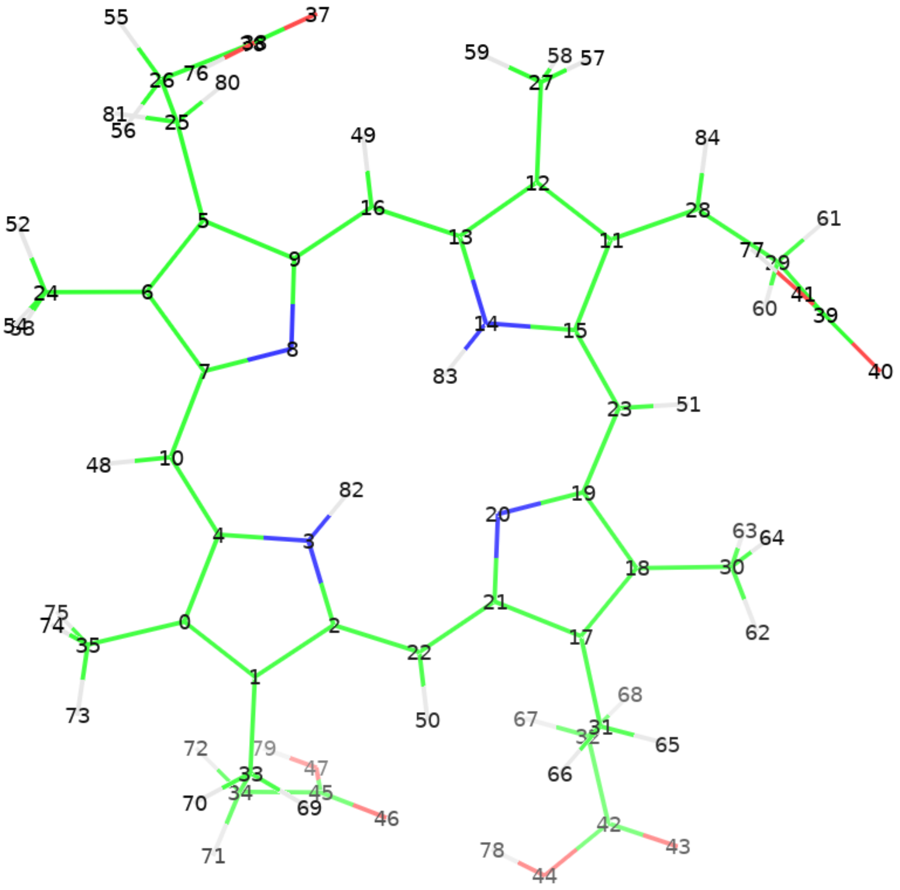

```
--------------------
ELECTRONIC G-MATRIX
--------------------

The g-matrix:
              2.0029947    0.0000823    0.0000746
              0.0003615    2.0031894    0.0001513
              0.0000002    0.0000083    2.0025189

 gel          2.0023193    2.0023193    2.0023193
 gRMC        -0.0001575   -0.0001575   -0.0001575
 gDSO(tot)    0.0002949    0.0003335    0.0001261
 gPSO(tot)    0.0000524    0.0003548    0.0010556
             ----------   ----------   ----------
 g(tot)       2.0025092    2.0028502    2.0033436 iso=  2.0029010
 Delta-g      0.0001899    0.0005309    0.0010243 iso=  0.0005817

Orientation:
  X          -0.0274000   -0.8401167   -0.5417132
  Y          -0.1076582    0.5412448   -0.8339447
  Z           0.9938103    0.0354698   -0.1052755
```

Euler rotation of hyperfine tensor to g-tensor

Atom	Alpha	Beta	Gamma	Ax	Ay	Az
		[degrees]			[MHz]	
0C	-115.2	14.7	111.6	13.57	1.39	1.66
1C	140.9	8.0	-144.6	-9.75	-3.47	-3.50
2C	-137.9	7.6	134.1	18.79	2.25	2.64
4C	-99.5	36.4	94.5	-9.69	-3.43	-3.84
5C	97.2	28.4	-100.5	1.21	2.17	1.85
6C	104.6	21.1	-107.1	-4.11	0.81	0.85
7C	-103.2	24.7	98.4	-14.52	-5.76	-4.47
9C	174.7	5.5	-177.8	-21.61	-7.43	-6.31
10C	-99.0	40.0	93.9	21.35	1.18	1.80
11C	95.6	43.5	-97.8	-54.06	-25.51	-29.68
12C	99.8	23.0	-103.1	84.71	6.55	5.51
13C	-96.5	39.4	91.3	-35.68	-16.63	-14.85
15C	178.8	4.7	177.5	20.63	2.47	3.27
16C	97.3	42.2	-98.9	33.22	3.29	3.57
17C	95.5	41.3	-97.0	-0.71	-2.57	-2.30
18C	-158.1	5.1	155.8	3.44	-1.62	-1.75
19C	-98.0	36.5	93.6	17.91	3.59	2.70
21C	112.0	15.2	-113.3	20.86	3.52	2.98
22C	99.9	42.7	-100.8	-19.23	-3.65	-4.57
23C	96.2	35.8	-99.4	-18.04	-4.59	-3.28
24C	-91.5	28.8	88.3	0.72	0.87	0.78
25C	43.1	33.4	-41.7	0.08	0.37	0.10
26C	143.8	35.6	-150.3	-0.83	-0.59	-0.75
27C	-74.0	41.3	62.7	-11.80	-10.89	-12.02
28C	-96.1	42.3	92.2	130.20	7.36	6.27
29C	-82.6	3.2	82.8	-20.09	-18.54	-20.88
30C	-19.1	37.3	11.8	-0.97	-0.83	-0.94
31C	-81.3	42.9	71.0	-0.44	-0.28	-0.51
32C	131.0	45.2	-171.4	1.16	0.80	0.87
33C	95.2	18.8	-118.7	0.72	0.80	0.88
34C	174.4	23.7	155.5	-1.91	-1.65	-1.62
35C	-99.9	23.4	103.5	-1.98	-2.03	-1.80
36C	-165.3	2.6	-173.8	-0.08	0.18	-0.05
39C	-65.2	23.4	103.2	51.77	43.89	44.14
42C	130.0	43.7	-96.3	0.09	0.08	0.19
45C	100.1	34.0	-70.9	0.02	0.03	0.10
48H	98.7	43.2	-99.1	-6.11	-2.39	-9.58
49H	-98.2	44.8	92.9	-10.30	-4.61	-12.90
50H	103.5	41.3	-106.0	3.83	7.16	2.44
51H	87.6	38.4	-98.5	2.10	1.16	8.64
52H	-101.3	44.8	93.0	-0.16	-0.02	0.04
53H	-16.2	31.3	0.9	-2.21	-1.64	-2.31
54H	-25.4	34.3	26.3	-2.93	-2.37	-2.81
55H	159.8	30.7	-155.1	-0.25	0.22	-0.24
56H	146.1	38.8	-139.4	-0.14	0.44	-0.08

57H	73.6	42.0	-75.2	22.83	24.86	28.73
58H	123.3	47.2	-123.9	32.73	34.20	38.34
59H	-71.7	29.8	66.1	0.22	4.98	0.71
60H	-38.1	27.4	44.3	36.15	43.50	36.60
61H	130.9	32.8	-130.7	4.32	11.83	3.63
62H	-89.2	18.2	85.1	-0.39	0.46	-0.17
63H	-108.6	28.4	121.1	2.84	3.74	3.43
64H	86.9	43.8	-103.8	2.01	2.72	3.06
65H	-102.3	36.1	93.5	-0.18	0.56	-0.09
66H	104.4	31.4	-113.0	0.23	0.16	1.08
67H	89.8	31.5	-45.6	-0.12	-0.29	0.46
68H	-106.9	38.9	138.0	-0.33	0.30	-0.22
69H	149.3	30.3	-112.7	-0.76	-0.80	-0.35
70H	106.4	31.4	-114.7	-1.22	-1.14	-0.84
71H	69.4	22.1	-83.8	-0.06	-0.01	0.10
72H	91.3	26.2	-63.1	-0.23	0.02	0.31
73H	-132.7	11.8	123.9	0.01	-0.07	0.79
74H	-111.6	38.0	118.2	4.25	4.46	5.19
75H	-88.4	32.7	68.2	5.21	5.36	6.13
76H	150.2	10.9	-120.3	-0.18	0.35	-0.15
77H	-57.6	6.8	82.1	7.16	-7.40	-9.66
78H	71.2	24.3	-76.0	-0.10	-0.07	0.16
79H	58.1	28.3	-35.0	-0.10	-0.05	0.16
80H	145.2	14.3	-155.0	-0.62	0.77	-0.75
81H	126.3	30.9	-136.8	-0.26	0.28	-0.32
82H	101.3	26.3	-103.1	-0.71	0.09	1.02
83H	-125.9	7.7	120.9	-7.01	-8.84	0.16
84H	96.5	27.5	-97.2	-37.89	-57.23	-16.34
3N	140.1	8.3	-143.2	-0.82	-0.40	-0.36
8N	-112.7	14.5	109.0	10.36	-0.10	0.09
14N	150.4	4.9	-154.5	7.87	-0.41	-0.17
20N	98.3	36.3	-99.1	-5.97	-0.44	-0.40

$A_{iso} = 20.8$ MHz (57H–59H)

CoproB-β-2-tautomer

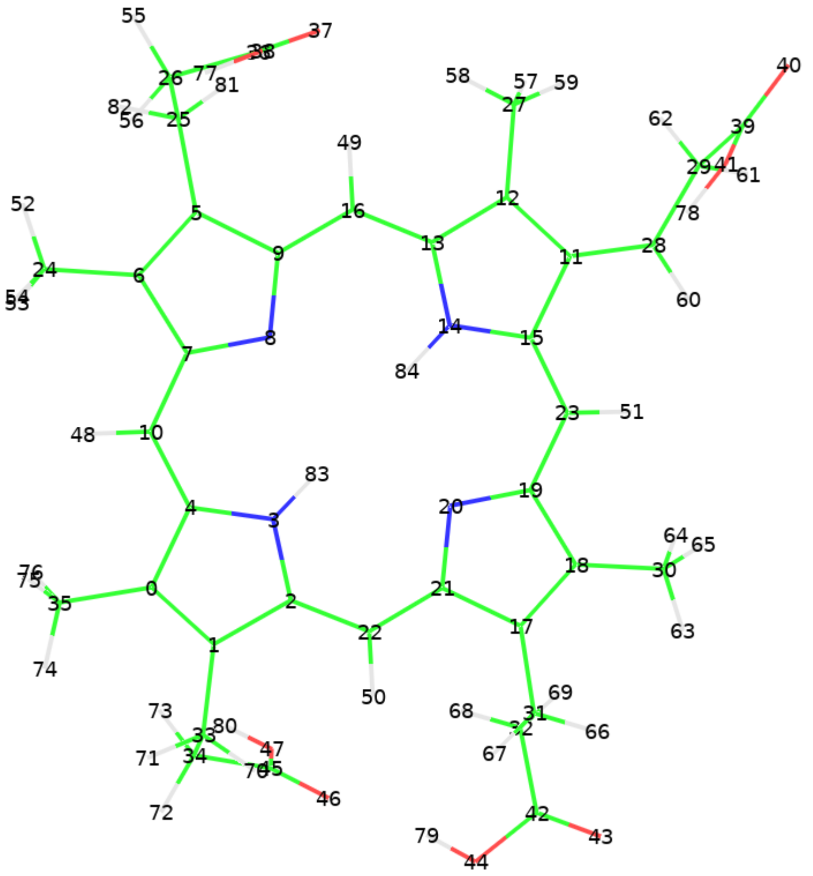

```
--------------------
ELECTRONIC G-MATRIX
--------------------

The g-matrix:
               2.0031940   -0.0003194    0.0001587
              -0.0003514    2.0030660   -0.0000290
              -0.0000503   -0.0002411    2.0023672

 gel           2.0023193    2.0023193    2.0023193
 gRMC         -0.0001601   -0.0001601   -0.0001601
 gDSO(tot)     0.0002354    0.0001292    0.0002448
 gPSO(tot)    -0.0000527    0.0005107    0.0010821
              ----------   ----------   ----------
 g(tot)        2.0023419    2.0027992    2.0034861 iso=  2.0028757
 Delta-g       0.0000226    0.0004799    0.0011668 iso=  0.0005565

Orientation:
  X            0.0118267    0.6519627   -0.7581588
  Y            0.1886346    0.7431433    0.6419930
  Z            0.9819761   -0.1506076   -0.1141938
```

Euler rotation of hyperfine tensor to g-tensor

Atom	Alpha	Beta [degrees]	Gamma	Ax	Ay [MHz]	Az
0C	-109.8	11.5	119.3	12.01	1.32	1.07
1C	-99.5	15.5	109.4	-8.20	-3.16	-3.15
2C	-133.8	4.4	144.0	17.31	2.51	2.10
4C	-96.1	38.6	105.2	-7.33	-3.13	-2.80
5C	121.7	30.9	-77.9	1.71	1.77	2.07
6C	116.1	10.6	-105.0	-4.20	0.61	0.57
7C	-101.7	21.4	111.2	-12.06	-3.83	-4.98
9C	148.1	6.1	-138.3	-20.06	-5.83	-6.82
10C	-97.9	34.4	106.6	17.40	1.37	0.86
11C	97.7	35.9	-86.1	-56.05	-31.08	-26.14
12C	102.3	24.8	-90.8	84.43	5.91	6.80
13C	-97.3	36.9	106.0	-34.97	-14.78	-16.46
15C	-171.5	4.5	-177.1	20.79	3.06	2.24
16C	-97.2	40.6	105.7	31.27	3.11	3.37
17C	94.6	42.0	-82.3	-1.01	-2.16	-2.42
18C	-118.4	10.0	127.8	3.70	-1.45	-1.32
19C	-96.6	36.5	104.6	14.31	2.01	2.82
21C	103.5	19.0	-93.9	18.82	2.59	3.04
22C	-94.0	43.4	101.4	-17.73	-3.35	-4.19
23C	97.4	38.7	-86.3	-13.52	-3.28	-4.68
24C	-104.0	23.4	117.9	0.70	0.75	0.82
25C	40.9	24.9	-85.2	0.01	-0.01	0.27
26C	90.7	23.6	-97.0	-0.58	-0.53	-0.38
27C	-66.1	42.0	74.7	-11.01	-11.93	-10.85
28C	-103.2	18.7	111.2	134.74	6.29	7.96
29C	95.0	11.9	-92.0	-19.91	-18.82	-20.95
30C	-91.9	12.6	112.9	-0.95	-0.95	-0.81
31C	-74.8	39.3	103.3	-0.36	-0.43	-0.21
32C	16.0	43.7	-40.3	1.03	0.77	0.71
33C	43.1	29.8	-29.6	0.56	0.70	0.61
34C	-9.3	27.8	6.3	-1.44	-1.21	-1.25
35C	-117.2	22.2	126.7	-1.78	-1.62	-1.83
36C	168.0	29.7	-160.0	-0.09	-0.06	0.16
39C	-57.9	6.2	29.9	59.52	50.51	50.79
42C	-132.2	42.4	105.7	0.08	0.07	0.17
45C	-139.8	55.8	120.8	-0.01	-0.00	0.07
48H	-97.0	42.8	105.1	-5.07	-1.90	-7.88
49H	-99.3	39.7	106.7	-9.77	-12.16	-4.34
50H	-90.7	44.6	97.2	3.52	6.63	2.26
51H	98.1	44.7	-87.5	0.66	7.57	0.19
52H	-95.0	41.7	101.5	-0.16	0.05	-0.05
53H	-91.5	5.1	54.9	-2.15	-2.06	-1.57
54H	-122.4	15.5	162.3	-2.71	-2.61	-2.22
55H	121.8	20.4	-125.6	-0.20	-0.19	0.26

56H	96.3	32.4	-116.9	-0.14	-0.08	0.42
57H	97.9	39.9	-115.0	40.19	45.31	41.32
58H	-96.0	23.1	120.9	1.71	2.31	6.37
59H	107.8	36.8	-78.2	21.62	27.05	23.77
60H	-101.9	15.6	109.5	-38.87	-60.20	-15.50
61H	-47.3	17.9	45.0	15.66	23.57	15.99
62H	99.9	27.1	-101.8	19.63	26.91	21.31
63H	-103.2	15.8	114.5	-0.39	-0.21	0.45
64H	-137.3	27.6	134.6	2.58	3.13	3.58
65H	-69.3	27.9	88.8	2.03	2.62	3.10
66H	-89.3	33.0	96.3	-0.21	-0.13	0.50
67H	77.1	34.8	-69.8	0.17	0.98	0.11
68H	-52.6	40.5	9.8	0.45	-0.26	-0.10
69H	-151.9	45.4	140.4	-0.31	-0.20	0.31
70H	135.9	51.5	-143.4	-0.73	-0.32	-0.76
71H	86.5	29.4	-80.7	-0.90	-0.51	-0.82
72H	72.0	24.5	-49.4	0.04	0.21	0.07
73H	146.9	40.4	-122.0	-0.21	0.29	0.00
74H	-68.3	7.0	76.6	-0.01	0.71	-0.08
75H	-122.2	34.8	119.8	3.87	4.74	4.05
76H	-62.4	34.0	82.9	4.76	5.61	4.89
77H	158.0	40.0	-150.4	-0.17	-0.14	0.34
78H	-48.8	11.1	37.1	6.93	-7.74	-8.96
79H	100.2	27.1	-78.3	-0.09	0.16	-0.07
80H	153.5	38.4	-117.7	-0.10	0.16	-0.05
81H	79.0	11.1	-75.1	-0.55	-0.65	0.82
82H	76.4	26.3	-79.1	-0.18	-0.23	0.37
83H	97.6	33.6	-86.5	-0.47	1.09	0.34
84H	-124.1	5.1	134.3	-6.93	0.23	-8.69
3N	135.5	6.5	-125.5	-1.16	-0.36	-0.42
8N	-114.2	12.0	123.7	9.26	0.09	-0.09
14N	142.0	4.6	-131.6	7.77	-0.22	-0.42
20N	98.2	31.2	-87.4	-5.09	-0.36	-0.41

$A_{\text{iso}} = 23.3$ MHz

Zn(II)CoproA-β-1

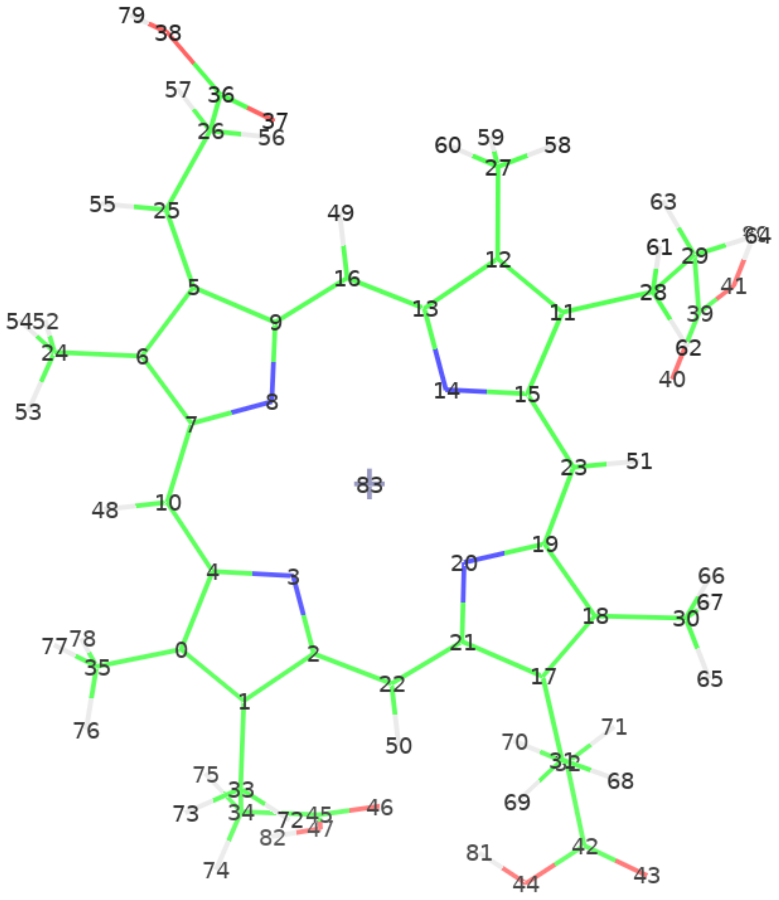


```
--------------------
ELECTRONIC G-MATRIX
--------------------

The g-matrix:
               2.0026446   -0.0000676    0.0000536
               0.0000510    2.0026670    0.0000587
              -0.0000512   -0.0001502    2.0020035

 gel           2.0023193    2.0023193    2.0023193
 gRMC         -0.0001672   -0.0001672   -0.0001672
 gDSO(tot)     0.0002411    0.0002019    0.0002217
 gPSO(tot)    -0.0003928    0.0002881    0.0002988
              ----------   ----------   ----------
 g(tot)        2.0020004    2.0026421    2.0026726 iso=  2.0024384
 Delta-g      -0.0003189    0.0003228    0.0003533 iso=  0.0001191

Orientation:
  X           -0.0009892   -0.9583415    0.2856233
  Y            0.0684961   -0.2850175   -0.9560718
  Z            0.9976509    0.0186183    0.0659246
```

Euler rotation of hyperfine tensor to g-tensor

Atom	Alpha	Beta [degrees]	Gamma	Ax	Ay [MHz]	Az
0C	17.5	6.6	-26.4	-8.14	-0.37	0.21
1C	31.5	4.8	-38.9	9.80	3.28	2.99
2C	77.7	32.5	-89.2	-39.62	-14.40	-12.35
4C	77.4	34.9	-88.7	-23.13	-12.76	-10.44
5C	-84.4	36.1	76.3	-10.85	-6.14	-5.57
6C	-40.7	8.5	32.4	37.09	1.78	2.09
7C	-71.7	24.3	64.1	-26.57	-14.56	-13.31
9C	101.2	30.3	-91.9	-5.48	-6.52	-7.51
10C	-68.8	21.4	60.9	60.64	1.82	0.98
11C	-16.8	7.9	5.6	-6.76	-1.06	-0.57
12C	71.9	31.2	-86.2	8.21	1.67	1.44
13C	77.1	36.7	-92.4	-20.32	-7.35	-6.30
15C	75.6	35.6	-80.2	-11.56	-10.26	-8.61
16C	-71.2	32.5	60.6	33.64	0.02	0.66
17C	12.2	9.6	-24.7	-5.54	0.53	-0.14
18C	1.6	13.2	-12.8	9.92	2.32	2.54
19C	74.6	37.2	-88.4	-35.27	-9.92	-11.53
21C	73.8	32.7	-86.2	-27.46	-11.15	-13.30
22C	-63.7	17.0	55.0	73.27	2.27	3.81
23C	-72.1	25.2	61.1	56.79	2.40	1.17
24C	-94.9	31.9	77.9	-4.92	-4.84	-5.44
25C	84.3	6.3	-88.7	10.31	0.87	0.93
26C	38.6	6.0	-35.0	-1.49	-1.38	-1.22
27C	69.7	39.3	-85.3	-1.04	-0.86	-1.07
28C	-74.6	45.4	68.5	0.62	0.83	1.13
29C	79.4	36.7	-117.0	-1.37	-0.75	-1.00
30C	70.0	35.7	-82.3	-1.17	-1.10	-0.88
31C	-69.7	40.3	59.3	0.40	1.15	0.82
32C	-136.6	44.0	119.4	-2.09	-0.82	-1.28
33C	32.8	43.6	-25.0	-1.01	-0.62	-0.80
34C	-37.9	48.4	32.7	1.44	1.50	1.94
35C	-79.2	38.9	72.1	0.86	1.25	1.59
36C	177.1	16.3	-162.2	4.41	3.64	3.81
39C	-130.6	43.4	96.6	0.38	0.18	0.80
42C	-106.1	24.2	150.2	-0.36	-0.27	-0.46
45C	-55.5	47.2	32.5	-0.25	-0.12	0.06
48H	-57.7	14.3	49.6	-18.87	-7.00	-27.61
49H	-63.2	21.2	51.7	-11.07	-15.84	-3.75
50H	-66.9	15.0	58.2	-21.98	-33.28	-8.06
51H	-69.0	16.9	55.3	-17.04	-5.44	-25.51
52H	-112.1	44.3	90.5	16.30	19.09	16.81
53H	-67.1	22.8	61.0	-0.71	2.09	-0.00
54H	-59.1	46.7	68.0	13.47	16.31	14.01
55H	-120.7	40.3	127.1	-3.87	-1.43	-4.12
56H	-141.1	8.8	144.6	1.38	2.07	3.25

$A_{iso} = 10.8$ MHz

57H	-123.9	9.5	122.8	1.02	1.20	2.30
58H	67.2	38.6	-84.2	-0.01	0.92	0.13
59H	103.2	43.6	-108.2	3.22	4.34	3.50
60H	59.5	43.8	-83.6	1.88	3.22	2.27
61H	71.4	32.5	-93.6	-2.18	-1.42	-1.99
62H	61.2	28.6	-78.6	-1.05	0.99	-1.29
63H	58.0	36.6	-52.7	-0.31	0.40	-0.08
64H	65.4	32.4	-66.6	0.04	0.60	0.14
65H	81.7	39.4	-88.8	-0.08	0.06	0.88
66H	72.5	42.5	-70.7	2.57	2.87	4.01
67H	85.1	37.5	-110.0	2.54	2.81	3.92
68H	78.5	29.9	-93.2	-0.35	-0.11	0.41
69H	92.2	31.0	-97.4	-2.51	-2.76	-0.33
70H	-70.5	42.4	107.1	0.25	2.62	0.62
71H	40.2	42.7	-58.9	0.44	0.53	1.10
72H	101.4	39.8	-109.7	-0.60	1.46	-0.49
73H	86.9	34.4	-85.8	1.53	2.61	1.69
74H	-57.1	48.3	63.7	0.11	0.15	0.80
75H	94.7	21.1	-137.4	-0.34	0.55	-0.16
76H	82.3	31.4	-92.1	-0.51	0.24	-0.19
77H	50.4	51.0	-37.9	-4.86	-2.91	-5.12
78H	-41.3	52.1	60.3	-6.77	-6.49	-4.90
79H	-15.5	16.8	-17.9	-0.18	-0.11	0.52
80H	41.6	42.5	-17.9	0.22	0.70	0.26
81H	-21.1	32.8	-10.7	-0.16	-0.23	0.68
82H	-57.2	55.3	34.8	-0.10	-0.07	0.38
3N	79.8	34.2	-90.2	15.36	-0.15	0.24
8N	74.1	15.1	-84.5	7.57	-0.43	-0.65
14N	76.4	34.5	-92.7	8.15	-0.50	-0.23
20N	69.2	32.4	-82.0	12.35	0.15	-0.22
83Zn	-52.4	8.1	39.3	-4.21	-7.29	-7.19

Zn(II)CoproA-β-2

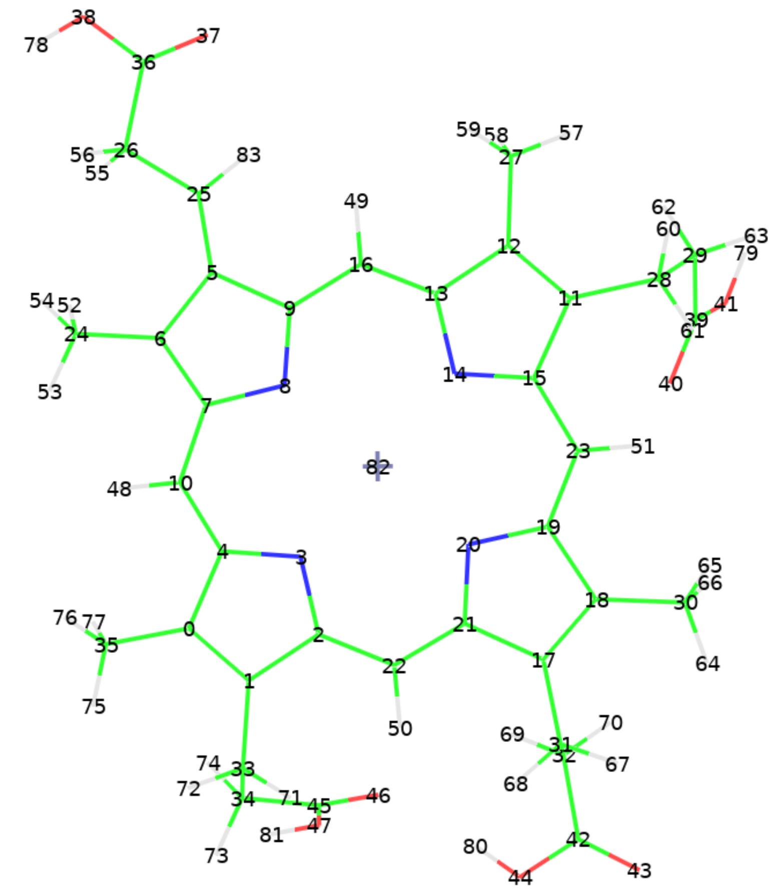

ELECTRONIC G-MATRIX

The g-matrix:

	2.0025929	0.0003104	-0.0000536
	-0.0003700	2.0025157	-0.0000601
	-0.0002181	0.0002630	2.0020438

gel	2.0023193	2.0023193	2.0023193		
gRMC	-0.0001661	-0.0001661	-0.0001661		
gDSO(tot)	0.0002270	0.0002398	0.0001919		
gPSO(tot)	-0.0003846	0.0001162	0.0003024		
	----------	----------	----------		
g(tot)	2.0019957	2.0025093	2.0026476	iso=	2.0023842
Delta-g	-0.0003236	0.0001900	0.0003283	iso=	0.0000649

Orientation:

X	0.2099731	-0.4332134	0.8764916
Y	-0.1757111	-0.8986008	-0.4020475
Z	0.9617884	-0.0695902	-0.2648024

Euler rotation of hyperfine tensor to g-tensor

Atom	Alpha	Beta	Gamma	Ax	Ay	Az
		[degrees]			[MHz]	
0C	116.7	46.8	-113.6	-7.98	0.44	-0.10
1C	-117.8	46.3	107.7	8.89	2.93	2.64
2C	-141.9	24.4	133.8	-39.53	-13.48	-11.41
4C	-148.6	22.1	140.8	-23.83	-12.39	-10.04
5C	176.7	18.6	176.4	-10.65	-5.38	-6.23
6C	120.7	39.9	-121.2	38.73	1.43	1.15
7C	133.7	28.4	-136.7	-26.28	-12.31	-13.62
9C	-137.4	28.2	128.4	-4.09	-5.36	-6.38
10C	135.1	27.1	-138.1	63.26	-0.14	0.53
11C	118.2	39.8	-116.9	-6.47	-0.62	-1.06
12C	-151.5	19.7	143.8	7.16	1.22	0.94
13C	-145.5	22.5	137.7	-17.16	-6.44	-5.45
15C	-98.6	13.5	84.9	-9.10	-9.82	-8.13
16C	150.7	21.6	-155.0	31.53	-0.35	-0.73
17C	114.7	48.6	-116.2	-5.41	-0.15	0.48
18C	-112.8	41.7	96.6	9.60	1.88	2.07
19C	-150.5	20.0	141.7	-34.15	-8.92	-10.52
21C	-140.3	22.8	131.0	-26.65	-10.49	-12.64
22C	125.8	32.4	-127.2	75.50	2.04	0.79
23C	142.8	25.5	-146.9	57.01	-0.16	0.82
24C	153.4	20.9	-158.1	-4.56	-5.39	-4.83
25C	-124.1	35.6	111.2	10.19	0.47	0.52
26C	-118.1	33.4	110.9	-1.35	-1.46	-1.17
27C	-20.6	25.5	11.8	-0.92	-0.73	-0.95
28C	-166.1	16.4	159.5	0.48	0.99	0.67
29C	178.3	35.2	157.0	-1.37	-0.79	-1.05
30C	-136.7	18.8	126.8	-1.10	-1.03	-0.82
31C	158.6	19.3	-172.1	0.31	0.74	1.07
32C	-172.4	38.7	-161.0	-2.19	-1.43	-0.94
33C	-59.8	37.8	52.9	-0.87	-0.46	-0.66
34C	109.4	14.5	-138.3	1.23	1.69	1.29
35C	169.4	19.1	-174.3	0.77	1.52	1.18
36C	80.3	44.4	-70.4	0.11	0.35	0.08
39C	-7.8	39.4	19.2	0.20	0.84	0.40
42C	42.4	20.5	-5.5	-0.37	-0.46	-0.27
45C	140.5	23.8	-173.3	-0.25	0.06	-0.11
48H	123.4	35.7	-124.4	-18.30	-27.95	-7.68
49H	128.1	30.9	-130.3	-9.75	-3.83	-14.44
50H	126.6	34.1	-125.2	-21.30	-8.66	-33.42
51H	133.2	36.1	-134.6	-16.05	-24.79	-5.44
52H	-166.7	42.0	172.1	17.78	18.28	20.78
53H	129.1	28.4	-133.0	-0.71	0.04	2.01
54H	-77.9	3.0	64.5	14.98	15.57	17.99
55H	-153.1	38.4	136.8	2.40	2.88	3.92

A_{iso} = 11.86 MHz

56H	-81.0	21.2	85.8	4.49	4.83	5.90
57H	-153.0	17.8	143.3	-0.13	0.79	0.00
58H	-163.3	29.5	172.3	2.75	3.90	3.04
59H	-176.6	12.2	158.3	1.72	3.02	2.09
60H	-149.5	22.3	135.2	-1.99	-1.19	-1.82
61H	-134.1	27.6	120.3	-1.03	1.02	-1.26
62H	-76.3	19.0	79.8	-0.33	0.40	-0.10
63H	-98.4	22.4	97.7	-0.05	0.56	0.05
64H	-149.0	18.7	147.2	-0.10	0.06	0.89
65H	-105.8	10.3	109.4	2.52	2.85	3.99
66H	-174.9	31.9	158.5	2.57	2.87	3.97
67H	-146.5	27.8	134.0	-0.35	-0.12	0.44
68H	-135.6	28.2	137.3	-2.54	-2.81	-0.35
69H	-19.5	18.3	35.1	0.27	0.59	2.66
70H	-76.1	12.9	67.3	0.46	0.54	1.14
71H	-160.4	35.3	162.6	-0.67	1.38	-0.59
72H	-125.6	21.8	130.2	1.46	2.51	1.60
73H	-155.3	3.0	141.5	0.12	0.78	0.16
74H	-168.7	46.0	135.5	-0.31	0.55	-0.14
75H	-145.1	26.1	139.7	-0.52	0.22	-0.20
76H	-37.4	23.4	35.7	-4.84	-2.86	-5.16
77H	-9.8	13.3	-8.2	-6.91	-4.99	-6.55
78H	-123.5	34.9	118.0	-0.21	-0.16	0.34
79H	-37.3	45.2	45.9	0.21	0.72	0.26
80H	-26.0	43.8	65.7	-0.24	-0.19	0.67
81H	154.4	22.2	169.5	-0.09	0.39	-0.06
83H	-134.2	21.5	122.7	-4.10	-2.25	-4.12
3N	-144.3	23.8	137.3	16.59	-0.19	0.18
8N	-122.7	38.7	109.9	5.86	-0.40	-0.63
14N	-142.5	24.0	133.2	8.60	-0.55	-0.30
20N	-134.5	22.8	124.1	12.65	0.06	-0.30
82Zn	123.5	44.5	-123.1	-3.88	-6.76	-6.86

Zn(II)CoproB-β-1

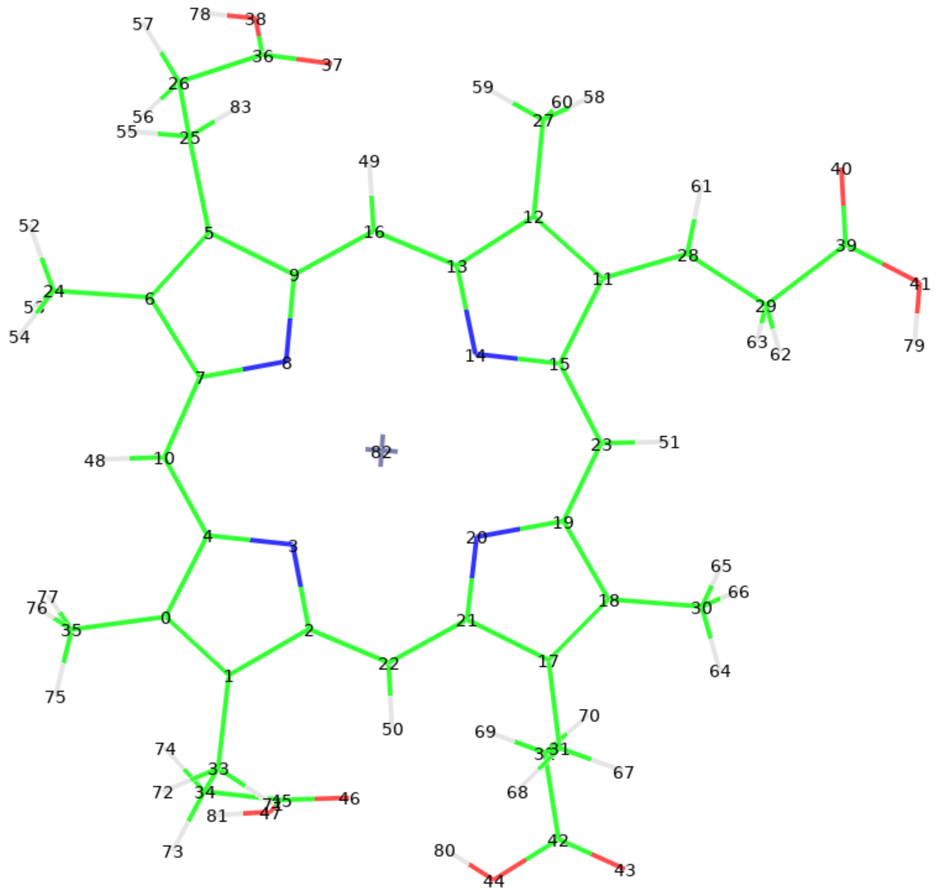

```
--------------------
ELECTRONIC G-MATRIX
--------------------

The g-matrix:
                 2.0027080   -0.0000629   -0.0000177
                 0.0001077    2.0025100    0.0000501
                -0.0000960    0.0001727    2.0022272

 gel             2.0023193    2.0023193    2.0023193
 gRMC           -0.0001583   -0.0001583   -0.0001583
 gDSO(tot)       0.0002419    0.0002076    0.0002153
 gPSO(tot)      -0.0002214    0.0001801    0.0003388
                ----------   ----------   ----------
 g(tot)          2.0021815    2.0025486    2.0027151 iso=   2.0024817
 Delta-g        -0.0001378    0.0002293    0.0003958 iso=   0.0001625

Orientation:
  X              0.1152069   -0.0147208   -0.9932324
  Y             -0.3261340    0.9439023   -0.0518186
  Z              0.9382771    0.3298967    0.1039431
```

Euler rotation of hyperfine tensor to g-tensor

Atom	Alpha	Beta [degrees]	Gamma	Ax	Ay [MHz]	Az
0C	51.6	12.5	-63.7	-9.59	-1.40	-1.84
1C	-76.3	35.0	65.4	9.63	1.87	1.46
2C	-77.5	40.4	69.7	-18.88	-7.87	-6.81
4C	-76.2	41.1	66.9	-4.54	-9.49	-7.83
5C	66.7	24.0	-74.6	-14.16	-3.73	-3.16
6C	-74.5	34.8	69.2	17.74	1.79	2.23
7C	-73.7	36.7	68.3	-22.57	-8.55	-9.83
9C	-73.1	45.8	70.9	2.79	-7.53	-9.49
10C	-57.2	17.2	49.4	45.25	-0.66	0.05
11C	-76.8	28.9	69.3	-8.60	-5.83	-5.30
12C	78.0	30.6	-89.0	32.21	0.80	1.07
13C	-22.1	7.4	13.6	-15.56	-10.92	-9.52
15C	77.1	34.3	-88.9	8.31	-3.14	-3.99
16C	-79.1	43.4	74.2	43.76	-2.15	-2.80
17C	11.3	7.1	-18.0	-7.31	-2.32	-2.04
18C	-75.0	29.0	69.0	7.72	0.02	0.44
19C	-73.2	36.8	60.1	-4.61	-3.56	-4.09
21C	-82.2	44.0	76.8	5.86	-5.49	-6.67
22C	-68.2	21.1	58.4	33.75	-1.08	-1.61
23C	76.5	33.4	-87.6	14.39	-1.98	-2.28
24C	-65.2	29.0	56.6	-2.37	-2.59	-2.23
25C	-62.8	25.6	61.2	1.19	1.55	1.87
26C	-73.8	24.0	99.1	-3.57	-2.98	-2.86
27C	-68.0	18.6	65.2	-4.11	-4.38	-4.97
28C	66.5	16.1	-79.3	2.74	0.36	0.56
29C	55.7	20.0	-80.1	-0.60	-0.67	-0.43
30C	-102.3	27.2	97.8	-1.18	-1.36	-1.09
31C	-96.0	38.8	85.9	0.19	0.28	0.69
32C	107.0	45.6	-123.5	-2.57	-0.65	-2.31
33C	-52.8	44.3	30.0	-1.14	-1.03	-1.31
34C	-164.8	25.5	-164.7	1.87	2.51	1.90
35C	-79.0	31.8	72.2	0.91	1.42	1.05
36C	29.2	45.7	-61.9	-0.23	0.17	-0.03
39C	79.0	18.2	-80.7	-0.05	-0.04	0.12
42C	-56.8	40.2	66.3	-1.39	-1.05	-0.49
45C	-48.8	13.1	66.3	-0.20	0.04	-0.12
48H	-6.2	9.2	-3.2	-14.75	-21.85	-5.09
49H	3.9	8.5	-10.9	-15.38	-4.67	-21.30
50H	-13.6	7.5	1.0	-11.56	-3.34	-16.74
51H	-41.5	10.0	32.7	-6.03	-8.04	-1.43
52H	-82.0	35.1	71.4	-0.17	-0.09	1.26
53H	-19.0	31.6	26.2	7.93	8.37	9.65
54H	-112.5	19.4	97.5	6.17	6.75	8.03
55H	83.8	42.4	-85.8	-2.66	-1.70	-2.35
56H	80.6	35.3	-115.4	-0.06	0.99	0.38

57H	-65.5	48.6	54.6	-0.49	-0.32	0.10
58H	-82.9	35.7	63.3	7.29	9.95	7.76
59H	-53.8	7.1	37.8	2.22	4.92	3.24
60H	-150.0	29.6	173.2	21.16	23.61	21.06
61H	76.2	39.9	-93.9	-1.85	-1.37	-0.26
62H	20.3	21.2	-37.8	0.80	1.11	2.09
63H	124.1	11.5	-125.1	0.84	1.15	2.18
64H	-71.9	36.7	71.8	-0.35	-0.21	0.66
65H	-125.0	19.0	105.2	3.99	4.37	5.21
66H	-24.7	27.5	29.0	3.72	4.10	4.96
67H	52.4	54.1	-50.6	-0.89	0.00	-0.69
68H	60.1	42.8	-73.1	-1.69	0.19	-1.79
69H	61.1	44.1	-31.6	2.21	3.96	2.80
70H	125.7	41.5	-132.1	-0.32	0.67	0.27
71H	-80.1	27.4	62.1	-0.00	1.49	0.33
72H	-94.7	38.1	76.6	1.90	3.01	2.07
73H	-132.6	35.6	139.4	-0.03	0.66	0.01
74H	-129.6	41.2	167.8	-0.30	0.39	-0.50
75H	-78.9	40.9	70.3	-0.58	0.24	-0.32
76H	139.4	45.3	-108.4	-4.20	-4.28	-2.34
77H	-96.6	38.1	109.9	-4.99	-3.26	-4.85
78H	40.8	41.9	-82.0	-0.16	0.41	-0.08
79H	50.6	9.1	-59.0	-0.18	-0.13	0.31
80H	53.4	27.4	-45.7	-0.46	0.42	-0.16
81H	-41.4	24.6	64.7	-0.14	0.31	-0.09
83H	114.4	49.6	-130.5	-2.06	0.26	-2.11
3N	-78.2	42.9	70.2	9.68	-0.63	-0.35
8N	-74.6	39.6	70.3	8.48	-0.37	-0.67
14N	76.4	37.8	-87.6	2.15	-0.53	-0.69
20N	-79.3	37.6	74.9	4.25	-0.66	-0.84
82Zn	70.8	37.8	-82.2	-2.23	-4.48	-4.45

A_{iso} = 11.2 MHz

Zn(II)CoproB-β-2

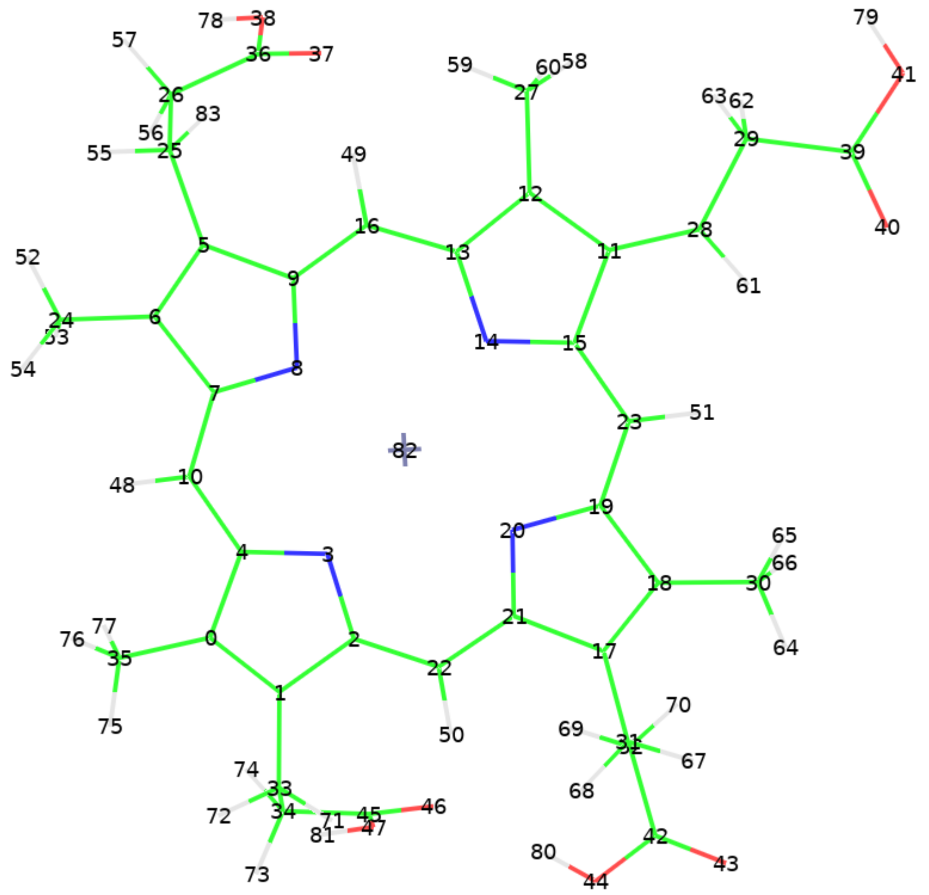

```
--------------------
ELECTRONIC G-MATRIX
--------------------

The g-matrix:
                2.0027332   -0.0000649   -0.0000141
                0.0000929    2.0025352    0.0000479
               -0.0001273    0.0002010    2.0021435

 gel            2.0023193    2.0023193    2.0023193
 gRMC          -0.0001621   -0.0001621   -0.0001621
 gDSO(tot)      0.0002420    0.0002081    0.0002158
 gPSO(tot)     -0.0002999    0.0002059    0.0003686
               ----------   ----------   ----------
 g(tot)         2.0020992    2.0025712    2.0027416 iso=  2.0024707
 Delta-g       -0.0002200    0.0002519    0.0004223 iso=  0.0001514

Orientation:
  X             0.1125211    0.0363599   -0.9929839
  Y            -0.2760450    0.9611367    0.0039134
  Z             0.9545356    0.2736679    0.1181851
```

Euler rotation of hyperfine tensor to g-tensor

Atom	Alpha	Beta	Gamma	Ax	Ay	Az
		[degrees]			[MHz]	
0C	56.9	14.9	-68.3	-8.09	-0.12	-0.63
1C	-78.6	45.7	69.7	8.24	2.51	2.22
2C	-77.0	40.1	70.3	-28.46	-10.24	-8.79
4C	-76.1	37.5	70.9	-17.07	-11.69	-9.73
5C	67.0	24.5	-73.2	-12.13	-2.53	-1.90
6C	-76.7	43.0	73.7	15.50	2.51	2.81
7C	-74.3	38.6	70.6	-31.63	-10.27	-11.91
9C	-54.5	43.5	65.0	-8.02	-9.10	-11.21
10C	-21.6	10.1	14.2	58.85	1.14	2.29
11C	-73.3	26.6	67.6	-10.21	-6.40	-5.57
12C	64.6	17.0	-73.7	36.69	1.46	1.74
13C	-11.3	7.4	4.5	-22.34	-12.97	-11.50
15C	76.3	41.1	-86.1	0.41	-4.42	-5.37
16C	-71.3	25.8	67.1	52.56	-0.59	-1.21
17C	54.4	13.6	-62.1	-6.10	-1.42	-1.05
18C	-77.2	34.6	72.8	7.66	0.67	0.93
19C	-76.9	34.9	71.7	-12.65	-4.94	-5.72
21C	-84.9	40.3	82.9	-5.28	-7.26	-8.70
22C	-37.5	9.9	28.0	47.42	0.93	0.00
23C	-71.5	23.7	65.5	23.76	-0.86	-0.53
24C	-85.4	32.5	64.3	-2.01	-2.03	-1.72
25C	-63.0	20.1	59.9	1.14	1.49	1.82
26C	-70.6	18.9	97.0	-3.49	-2.89	-2.76
27C	-60.3	15.4	56.1	-4.59	-4.91	-5.50
28C	53.7	13.1	-60.8	6.87	0.59	0.66
29C	43.6	31.9	-86.0	-1.06	-1.08	-0.82
30C	-101.2	30.0	96.7	-1.07	-1.19	-0.96
31C	-81.6	34.8	81.4	0.15	0.33	0.72
32C	-99.7	43.5	69.9	-2.10	-1.78	-0.34
33C	136.8	46.3	-107.3	-0.92	-0.78	-0.64
34C	-140.2	12.8	166.0	1.23	1.69	1.27
35C	-72.3	26.6	65.3	0.86	1.48	1.11
36C	29.4	47.5	-58.1	-0.21	0.20	-0.02
39C	122.5	20.1	-82.8	0.09	0.11	0.33
42C	-53.7	36.2	64.8	-1.42	-1.12	-0.61
45C	-41.1	18.4	59.2	-0.18	0.06	-0.10
48H	12.1	9.5	-20.4	-18.02	-27.37	-6.70
49H	24.7	9.5	-29.9	-17.46	-5.78	-24.61
50H	13.4	7.8	-25.1	-14.99	-4.97	-22.30
51H	-13.2	7.3	6.0	-8.31	-11.57	-2.67
52H	-76.0	33.9	71.8	-0.12	-0.02	1.16
53H	-35.3	34.9	38.7	5.41	5.81	7.06
54H	-102.9	25.8	93.3	4.41	4.86	6.23
55H	-85.0	43.4	86.4	-2.26	-1.96	-1.40

56H	78.2	41.2	-111.8	-0.05	0.93	0.38
57H	-62.3	44.5	53.2	-0.46	-0.30	0.10
58H	-105.7	32.3	84.2	13.90	16.81	14.48
59H	-34.2	5.8	23.6	-0.40	2.29	0.48
60H	-28.6	43.6	40.9	19.75	22.64	20.17
61H	-79.7	35.1	73.5	-3.28	-2.88	-1.64
62H	84.5	37.3	-110.8	2.81	3.05	4.07
63H	70.3	41.6	-70.8	1.14	1.50	2.58
64H	-74.6	36.2	72.9	-0.28	-0.11	0.67
65H	-115.5	24.8	99.3	3.42	3.82	4.60
66H	-35.5	31.4	40.3	3.32	3.71	4.51
67H	55.9	52.0	-54.1	-0.55	0.28	-0.35
68H	76.5	42.5	-93.6	-1.90	0.01	-2.04
69H	68.7	46.9	-37.4	2.35	4.16	2.77
70H	123.3	44.7	-131.3	-0.23	0.66	0.30
71H	-80.9	33.6	64.8	-0.30	1.25	-0.11
72H	-94.5	39.1	78.0	1.45	2.40	1.60
73H	-105.1	27.6	109.7	-0.04	0.57	-0.00
74H	-132.5	45.7	171.7	-0.22	0.41	-0.37
75H	-76.7	38.4	68.5	-0.44	0.29	-0.19
76H	-122.3	49.9	117.7	-4.59	-2.71	-4.79
77H	-117.8	37.8	134.2	-5.72	-3.93	-5.51
78H	40.3	45.8	-79.4	-0.17	0.40	-0.09
79H	69.2	37.4	-80.8	-0.18	-0.14	0.32
80H	52.7	28.0	-42.3	-0.37	0.50	-0.13
81H	-39.1	27.3	61.7	-0.12	0.31	-0.08
83H	-87.7	41.4	85.3	-2.21	-2.30	0.09
3N	-77.8	43.0	71.2	12.81	-0.41	-0.08
8N	-73.8	38.6	71.0	11.28	-0.10	-0.45
14N	75.2	35.1	-84.5	3.82	-0.39	-0.57
20N	-79.3	39.3	76.2	6.15	-0.41	-0.63
82Zn	64.3	28.5	-73.3	-3.25	-5.87	-5.81

$A_{iso} = 12.2$ MHz

Zn(0)CoproA-β-1

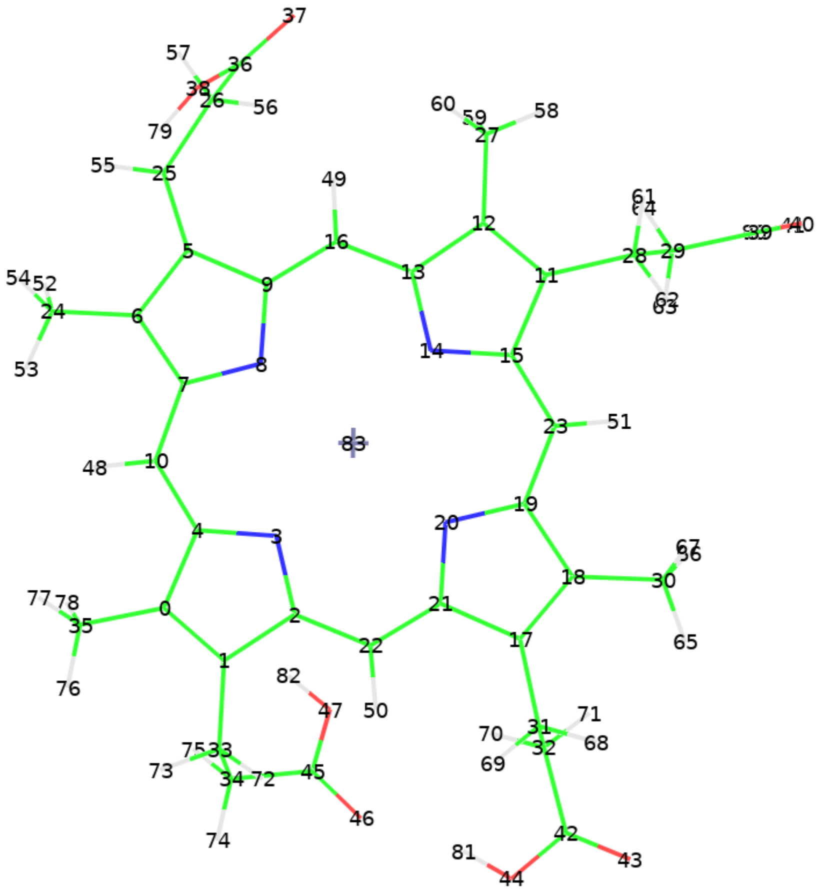


```
--------------------
ELECTRONIC G-MATRIX
--------------------

The g-matrix:
              2.0032913   -0.0003361   -0.0000182
             -0.0000413    2.0030079    0.0001122
              0.0001708    0.0000363    2.0024431

 gel          2.0023193    2.0023193    2.0023193
 gRMC        -0.0001585   -0.0001585   -0.0001585
 gDSO(tot)    0.0003795    0.0004043    0.0001378
 gPSO(tot)   -0.0001190    0.0003690    0.0010883
             ----------   ----------   ----------
 g(tot)       2.0024213    2.0029341    2.0033870 iso=  2.0029141
 Delta-g      0.0001020    0.0006148    0.0010677 iso=  0.0005948

Orientation:
  X          -0.1212003    0.4239902   -0.8975204
  Y          -0.1629043    0.8834303    0.4393325
  Z           0.9791694    0.1994572   -0.0380022
```

Euler rotation of hyperfine tensor to g-tensor

Atom	Alpha	Beta	Gamma	Ax	Ay	Az
		[degrees]			[MHz]	
0C	85.7	18.5	-89.9	5.29	1.86	1.59
1C	-83.3	27.5	79.5	-5.74	-0.85	-0.76
2C	-85.0	33.6	83.8	-0.39	-1.98	-1.38
4C	-84.1	23.3	80.6	-15.89	-5.60	-4.79
5C	86.9	32.2	-91.6	-52.83	-24.69	-28.89
6C	84.6	18.7	-88.7	92.21	6.34	5.26
7C	-87.0	40.3	83.6	-33.55	-17.16	-15.59
9C	-58.4	6.0	54.2	6.80	-0.03	0.32
10C	-75.8	8.3	71.6	27.21	2.71	2.64
11C	83.5	27.2	-90.6	-2.82	-2.15	-1.96
12C	-83.5	30.1	80.4	5.24	-0.38	-0.52
13C	85.1	38.8	-89.8	6.17	0.42	1.00
15C	-30.9	3.4	26.6	14.27	1.97	1.75
16C	93.0	26.9	-91.1	-4.49	-2.01	-1.28
17C	-88.7	26.8	86.4	5.07	-0.24	0.02
18C	54.0	2.1	-56.1	-3.30	-2.00	-1.89
19C	-84.1	21.9	80.7	13.36	1.31	1.65
21C	71.0	7.6	-75.6	2.66	-0.46	-0.24
22C	-79.1	11.3	74.8	2.62	-0.85	-0.65
23C	84.4	35.1	-89.1	-12.75	-2.67	-3.30
24C	87.8	40.1	-91.4	-13.27	-13.40	-12.11
25C	87.3	42.8	-93.7	128.72	6.26	7.33
26C	-109.5	11.8	101.4	-20.04	-18.42	-20.76
27C	-120.7	37.2	124.2	-1.03	-0.85	-0.99
28C	131.5	37.9	-89.0	-0.04	-0.07	0.11
29C	-60.2	42.0	25.6	0.08	0.27	0.15
30C	37.0	7.8	-43.6	0.14	0.06	0.20
31C	-97.7	32.8	87.0	-0.83	-0.90	-0.72
32C	-58.3	42.1	29.5	1.59	1.18	1.23
33C	44.5	13.5	-50.5	0.66	0.78	0.75
34C	-12.9	19.7	-12.0	-1.47	-1.17	-1.20
35C	54.8	9.2	-59.2	-0.71	-0.39	-0.63
36C	-30.5	38.3	72.4	42.35	49.93	42.67
39C	71.1	28.4	-109.8	-0.02	-0.01	0.06
42C	-65.6	43.3	75.8	0.14	0.13	0.24
45C	149.6	51.2	-139.2	0.06	0.15	0.05
48H	86.8	42.4	-91.9	-8.81	-10.20	-3.80
49H	71.8	33.1	-91.5	-1.16	-0.45	3.22
50H	-89.2	40.1	83.7	-1.41	-1.98	0.00
51H	82.6	31.8	-86.9	2.33	4.60	1.69
52H	55.2	36.0	-61.1	31.36	33.18	37.54
53H	-87.4	36.7	83.9	-0.75	4.38	-0.48
54H	119.7	33.9	-122.7	31.18	33.03	37.33
55H	85.9	19.3	-92.6	-37.34	-56.27	-16.16
56H	-131.8	31.6	117.4	38.03	45.28	38.52

$A_{iso} = 23.0$ MHz

57H	45.2	24.0	-53.9	3.31	10.81	2.56
58H	-95.1	31.7	94.5	-0.22	0.62	-0.05
59H	-83.8	37.2	63.9	2.83	3.83	3.29
60H	92.3	42.3	-81.9	1.77	2.37	2.98
61H	81.8	40.7	-82.2	-0.40	-0.31	0.21
62H	86.5	25.2	-74.4	-0.20	-0.21	0.46
63H	179.4	29.9	-137.1	-0.17	-0.13	0.38
64H	103.7	41.8	-122.1	-0.23	-0.16	0.32
65H	3.6	2.8	-2.7	-0.29	-0.24	0.10
66H	83.0	19.2	-127.9	-0.18	-0.10	0.31
67H	91.7	22.5	-61.0	-0.30	-0.22	0.20
68H	-111.2	22.1	71.7	0.36	0.38	0.94
69H	79.2	41.6	-90.8	0.70	1.35	0.81
70H	74.1	37.9	-36.1	-0.36	0.14	-0.19
71H	-107.8	36.3	152.1	-0.23	-0.31	0.16
72H	36.9	6.7	-45.2	-0.77	-0.32	-0.74
73H	87.0	32.2	-94.9	-0.90	-0.57	-0.84
74H	67.6	24.4	-60.6	-0.17	0.02	-0.10
75H	133.5	36.1	-131.8	-0.12	0.35	0.05
76H	98.4	4.6	-104.1	-0.08	0.59	-0.07
77H	-19.9	2.2	5.3	0.50	1.76	0.61
78H	39.0	1.7	-32.2	0.69	1.84	0.80
79H	-118.2	18.5	84.5	7.24	-7.34	-9.61
80H	113.3	37.8	-129.6	-0.08	-0.07	0.15
81H	85.1	40.6	-67.7	-0.10	0.15	-0.06
82H	55.8	28.4	-24.4	-0.27	0.28	-0.02
3N	-83.2	24.5	79.4	5.31	-0.00	0.15
8N	76.0	9.5	-80.3	11.47	-0.15	0.09
14N	76.1	12.6	-80.4	-3.13	-0.65	-0.58
20N	-83.7	14.6	79.8	-1.52	-0.35	-0.29
83Zn	-86.0	41.4	86.2	-2.31	-2.57	-2.15

Zn(0)CoproA-β-2

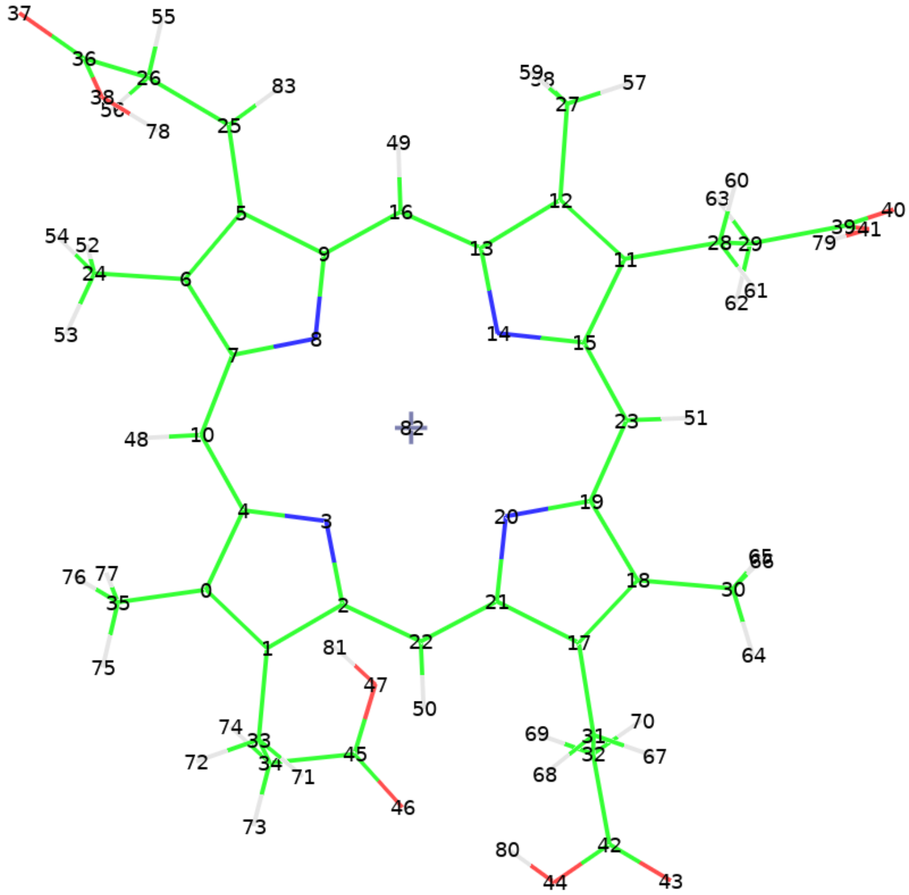


```
--------------------
ELECTRONIC G-MATRIX
--------------------

The g-matrix:
                2.0031296    0.0003689    0.0002114
                0.0002575    2.0031322    0.0002696
                0.0004226    0.0000195    2.0023874

 gel            2.0023193    2.0023193    2.0023193
 gRMC          -0.0001604   -0.0001604   -0.0001604
 gDSO(tot)      0.0003118    0.0001433    0.0003181
 gPSO(tot)     -0.0002012    0.0005394    0.0010613
               ----------   ----------   ----------
 g(tot)         2.0022694    2.0028416    2.0035382 iso=  2.0028831
 Delta-g       -0.0000499    0.0005223    0.0012189 iso=  0.0005638

Orientation:
  X            -0.3338311    0.6193398    0.7106160
  Y            -0.0365867   -0.7618080    0.6467688
  Z             0.9419226    0.1899124    0.2769748
```

Euler rotation of hyperfine tensor to g-tensor

Atom	Alpha	Beta<br>[degrees]	Gamma	Ax	Ay<br>[MHz]	Az
0C	79.5	35.7	-90.7	6.11	1.55	1.82
1C	-64.3	11.8	57.4	-5.97	-1.03	-1.13
2C	-75.5	16.5	68.8	1.61	-0.98	-1.50
4C	-49.5	7.7	40.6	-15.20	-4.69	-5.47
5C	83.2	40.4	-96.7	-54.75	-30.23	-25.12
6C	81.5	37.0	-94.0	92.21	5.80	6.75
7C	-77.7	23.6	67.8	-33.37	-15.60	-17.12
9C	46.6	8.6	-60.0	6.39	0.25	-0.11
10C	56.5	9.1	-67.2	26.97	2.53	2.60
11C	72.4	41.8	-86.4	-3.65	-2.02	-2.20
12C	-59.1	20.1	47.7	6.08	-0.16	0.00
13C	-78.4	41.0	68.8	2.68	-0.34	0.25
15C	56.5	17.7	-68.0	13.75	1.63	1.81
16C	79.3	43.7	-97.3	0.38	-1.18	-2.00
17C	-49.4	12.1	39.4	4.23	-0.28	-0.53
18C	78.6	22.0	-90.1	-2.26	-1.78	-1.91
19C	-26.0	9.4	17.0	13.21	1.71	1.34
21C	71.6	23.9	-81.7	4.71	0.21	-0.06
22C	68.8	22.3	-81.1	-0.08	-1.00	-1.23
23C	-75.2	36.1	68.7	-12.58	-2.62	-3.24
24C	-112.0	35.8	102.5	-12.31	-13.24	-11.99
25C	-69.7	11.0	58.9	131.17	6.08	7.79
26C	89.0	18.3	-94.9	-19.39	-18.28	-20.33
27C	-48.3	14.0	50.4	-1.10	-1.09	-0.92
28C	-41.3	35.4	70.3	0.05	0.08	0.22
29C	-1.8	29.5	24.3	-0.10	0.00	0.11
30C	72.3	26.8	-78.5	-0.01	0.05	-0.10
31C	-49.3	20.3	38.4	-0.76	-0.65	-0.83
32C	-4.2	30.4	29.3	1.46	1.12	1.08
33C	85.1	37.1	-88.3	0.63	0.72	0.76
34C	55.0	33.1	-44.3	-1.35	-1.09	-1.07
35C	82.3	25.1	-88.1	-0.84	-0.77	-0.53
36C	171.5	9.3	-146.8	57.97	49.26	49.55
39C	-28.6	49.6	57.5	-0.01	-0.01	0.06
42C	-115.1	28.6	116.8	0.13	0.22	0.12
45C	-81.0	43.6	38.0	0.05	0.14	0.04
48H	-74.0	29.1	68.0	-8.74	-10.19	-3.74
49H	-80.9	36.7	69.7	-2.52	-1.39	2.21
50H	-49.4	15.3	40.8	-0.75	0.43	-0.96
51H	-77.8	38.3	71.6	2.30	4.56	1.66
52H	87.0	43.4	-70.9	42.03	47.58	43.32
53H	-76.8	16.8	54.7	0.67	1.08	5.65
54H	72.2	41.4	-103.4	26.77	32.55	28.83
55H	-157.2	17.7	155.6	13.69	21.44	14.02

A_{iso} = 25.4 MHz

56H	85.7	32.1	-85.6	20.71	27.89	22.51
57H	-57.0	14.4	44.6	-0.26	-0.13	0.58
58H	-28.9	25.7	31.0	2.78	3.23	3.90
59H	-105.9	19.0	84.8	2.18	2.66	3.34
60H	-81.7	33.2	72.1	-0.47	-0.40	0.10
61H	112.0	49.0	-133.3	-0.30	0.30	-0.32
62H	-145.0	40.8	-175.3	0.43	-0.13	-0.08
63H	-51.9	45.6	36.8	-0.22	-0.14	0.34
64H	85.3	17.8	-96.8	-0.29	0.13	-0.24
65H	128.2	45.2	-169.4	0.30	0.74	0.23
66H	123.2	50.6	-138.0	0.11	0.64	0.20
67H	-4.6	40.2	-1.8	0.24	0.80	0.26
68H	-63.9	33.3	64.4	0.73	1.37	0.83
69H	-136.9	51.3	110.1	-0.34	0.15	-0.18
70H	-169.9	35.5	129.0	-0.23	0.16	-0.30
71H	72.1	29.7	-81.4	-0.69	-0.67	-0.25
72H	-78.5	40.2	74.0	-0.98	-0.62	-0.92
73H	104.0	42.5	-107.9	-0.17	-0.10	0.03
74H	-70.9	48.9	40.3	-0.13	0.35	0.03
75H	62.8	22.7	-83.7	-0.08	-0.06	0.61
76H	45.3	23.1	-58.2	0.83	0.98	2.10
77H	111.3	19.3	-120.5	1.13	1.27	2.26
78H	-144.0	13.8	150.8	6.86	-7.29	-8.15
79H	-52.1	52.3	39.1	-0.08	-0.07	0.15
80H	-121.9	36.5	102.9	-0.10	0.14	-0.06
81H	138.5	49.3	-134.9	-0.28	-0.02	0.31
83H	-70.2	8.9	59.9	-37.97	-58.78	-15.21
3N	-43.9	8.4	36.3	4.74	0.14	-0.00
8N	79.7	28.2	-92.2	10.55	0.05	-0.16
14N	71.1	27.4	-85.0	-2.51	-0.50	-0.58
20N	32.8	9.0	-43.0	-1.95	-0.32	-0.39
82Zn	-92.8	26.0	78.8	-2.03	-1.92	-2.32

Zn(0)CoproB-β-1

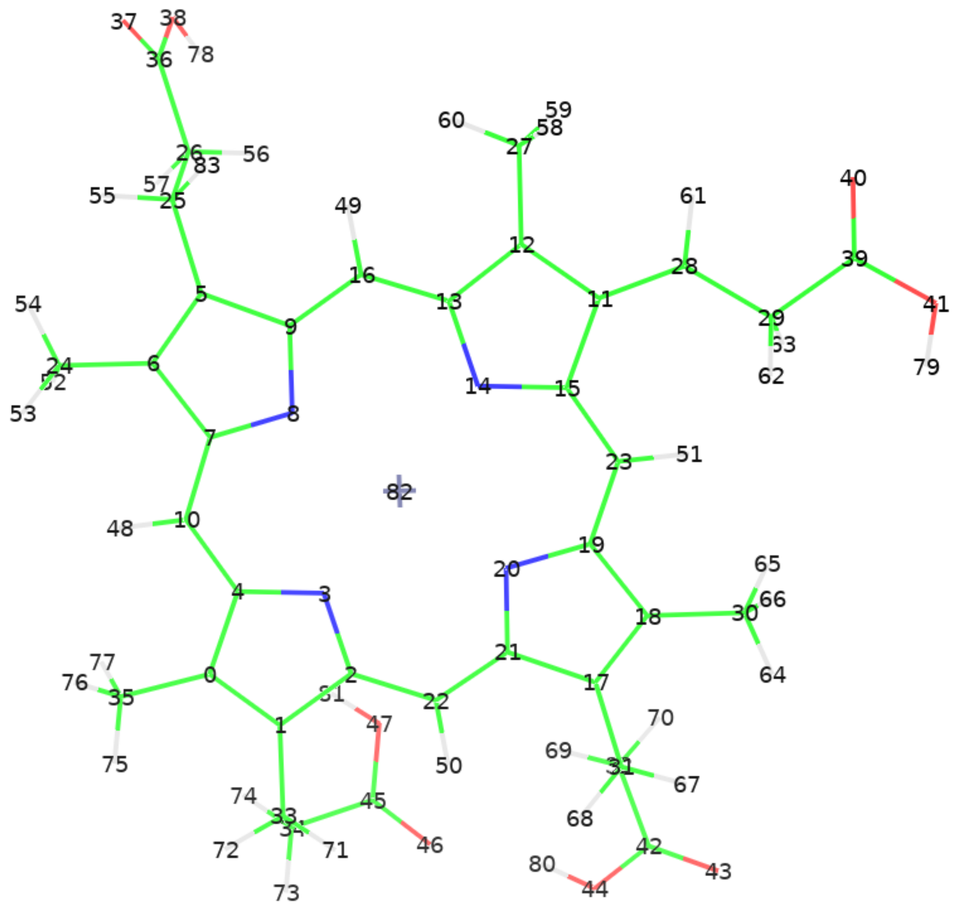

```
--------------------
ELECTRONIC G-MATRIX
--------------------

The g-matrix:
              2.0029455    0.0000131   -0.0000189
              0.0000616    2.0030173    0.0001374
             -0.0000303    0.0001235    2.0021187

 gel          2.0023193    2.0023193    2.0023193
 gRMC        -0.0001537   -0.0001537   -0.0001537
 gDSO(tot)    0.0002966    0.0003100    0.0001570
 gPSO(tot)   -0.0003631    0.0004598    0.0007244
             ----------   ----------   ----------
 g(tot)       2.0020991    2.0029354    2.0030470 iso=  2.0026938
 Delta-g     -0.0002202    0.0006161    0.0007278 iso=  0.0003746

Orientation:
  X           0.0349931   -0.9480574   -0.3161687
  Y          -0.1419742    0.3084380   -0.9405899
  Z           0.9892516    0.0778020   -0.1238065
```

Euler rotation of hyperfine tensor to g-tensor

Atom	Alpha	Beta [degrees]	Gamma	Ax	Ay [MHz]	Az
0C	-91.6	28.8	91.3	7.85	0.02	0.26
1C	92.3	12.8	-95.8	-3.97	-2.39	-2.32
2C	-89.5	28.0	88.7	13.60	1.40	1.76
4C	90.4	17.8	-90.9	-0.35	-1.61	-1.36
5C	90.1	14.0	-93.9	4.28	2.10	1.80
6C	-91.3	20.8	88.8	-5.69	-0.33	-0.24
7C	-90.9	36.5	89.3	-4.93	-3.24	-2.41
9C	-87.8	24.8	85.0	-18.36	-6.34	-5.42
10C	-90.8	38.1	89.3	9.47	-0.32	-0.05
11C	83.1	24.9	-86.5	-57.98	-25.08	-29.50
12C	82.6	18.7	-84.5	99.44	7.24	6.06
13C	-87.2	43.9	85.6	-36.70	-18.66	-16.87
15C	-80.1	21.2	77.8	2.43	-0.01	0.14
16C	-83.9	20.4	81.6	29.50	3.17	3.22
17C	83.6	17.5	-78.0	-3.74	-2.31	-2.13
18C	-84.5	37.0	88.1	6.40	0.00	-0.15
19C	84.6	26.7	-84.5	3.07	-0.23	0.32
21C	-84.3	11.5	87.2	14.87	2.00	1.76
22C	91.9	29.2	-91.2	-13.81	-2.77	-3.44
23C	83.9	18.8	-80.8	0.78	-1.30	-0.72
24C	-93.3	27.1	90.6	0.76	0.89	0.85
25C	173.2	30.6	-174.9	-0.45	-0.15	-0.40
26C	23.5	33.7	-12.4	0.07	0.15	-0.04
27C	84.0	31.2	-89.5	-14.16	-14.39	-13.10
28C	85.9	37.2	-90.7	129.39	5.74	6.95
29C	-84.0	12.9	80.5	-20.84	-19.30	-21.59
30C	-84.1	36.7	86.7	-1.19	-0.97	-1.12
31C	46.1	31.4	-88.9	0.08	0.05	0.22
32C	-116.9	43.1	153.1	-0.10	0.13	0.01
33C	65.4	3.7	-98.6	0.06	0.10	0.22
34C	-1.7	23.9	46.6	-0.04	-0.11	0.04
35C	-84.9	38.7	93.0	-1.31	-1.36	-1.15
36C	-60.6	14.8	45.1	-0.05	0.09	-0.05
39C	71.2	23.6	-69.1	1.06	2.06	0.51
42C	49.9	28.5	-65.4	0.02	0.03	0.10
45C	-5.1	33.8	-30.1	-0.02	-0.03	0.07
48H	91.2	33.0	-92.1	-3.22	-0.90	-4.80
49H	85.3	33.8	-87.9	-9.36	-10.85	-4.18
50H	98.3	27.1	-98.6	2.59	5.05	1.79
51H	83.8	28.3	-82.3	-2.26	-1.18	1.07
52H	-140.6	27.3	132.5	-2.93	-2.48	-2.81
53H	-27.2	34.5	28.5	-2.44	-1.95	-2.35
54H	85.7	27.6	-88.0	-0.22	0.09	-0.13
55H	6.4	13.8	-5.0	0.15	0.81	0.15
56H	-168.8	29.0	143.3	-0.50	0.85	-0.45

57H	7.5	45.0	-30.0	-0.18	0.44	-0.14
58H	44.4	29.1	-49.9	33.54	35.54	39.89
59H	130.5	26.4	-130.1	35.94	37.74	42.17
60H	-78.6	45.4	75.2	-0.62	4.75	-0.37
61H	75.4	10.5	-79.8	-38.56	-56.92	-16.80
62H	-112.2	30.9	95.7	46.66	54.22	47.43
63H	-39.8	39.3	47.7	54.86	62.45	55.51
64H	-83.2	36.1	85.7	-0.28	0.55	-0.13
65H	-85.5	42.7	104.2	2.51	3.62	3.02
66H	85.1	44.5	-99.6	2.35	2.88	3.49
67H	88.2	32.7	-90.0	-0.47	-0.40	0.10
68H	71.8	19.8	-101.4	-0.29	-0.31	0.33
69H	69.3	24.6	-24.6	-0.05	-0.13	0.46
70H	52.5	37.9	-46.6	-0.24	-0.16	0.32
71H	69.0	20.7	-107.2	-0.27	-0.23	0.28
72H	164.3	7.2	-171.3	-0.49	-0.42	-0.02
73H	7.9	11.2	-17.7	-0.13	-0.10	0.13
74H	26.0	20.3	-2.4	-0.19	-0.11	0.24
75H	-111.9	26.6	93.6	-0.09	-0.12	0.58
76H	92.3	38.3	-76.4	2.66	3.47	2.81
77H	92.5	40.1	-112.2	3.50	4.29	3.60
78H	-138.8	17.3	108.4	-0.15	0.28	-0.14
79H	1.7	18.4	-7.5	-0.85	1.34	-0.77
80H	47.9	21.7	-41.5	-0.08	-0.06	0.15
81H	38.3	44.9	-3.4	-0.20	-0.11	0.32
83H	-147.8	14.5	152.8	-0.28	1.14	-0.25
3N	-91.6	26.7	90.0	-1.36	-0.35	-0.29
8N	-90.2	30.0	88.1	7.13	0.13	0.30
14N	70.7	5.3	-72.9	12.00	-0.03	0.24
20N	43.4	3.6	-42.3	-2.75	-0.63	-0.56
82Zn	85.4	36.5	-94.0	-2.86	-2.65	-3.15

A_{iso} = 25.4 MHz

Zn(0)CoproB-β-2

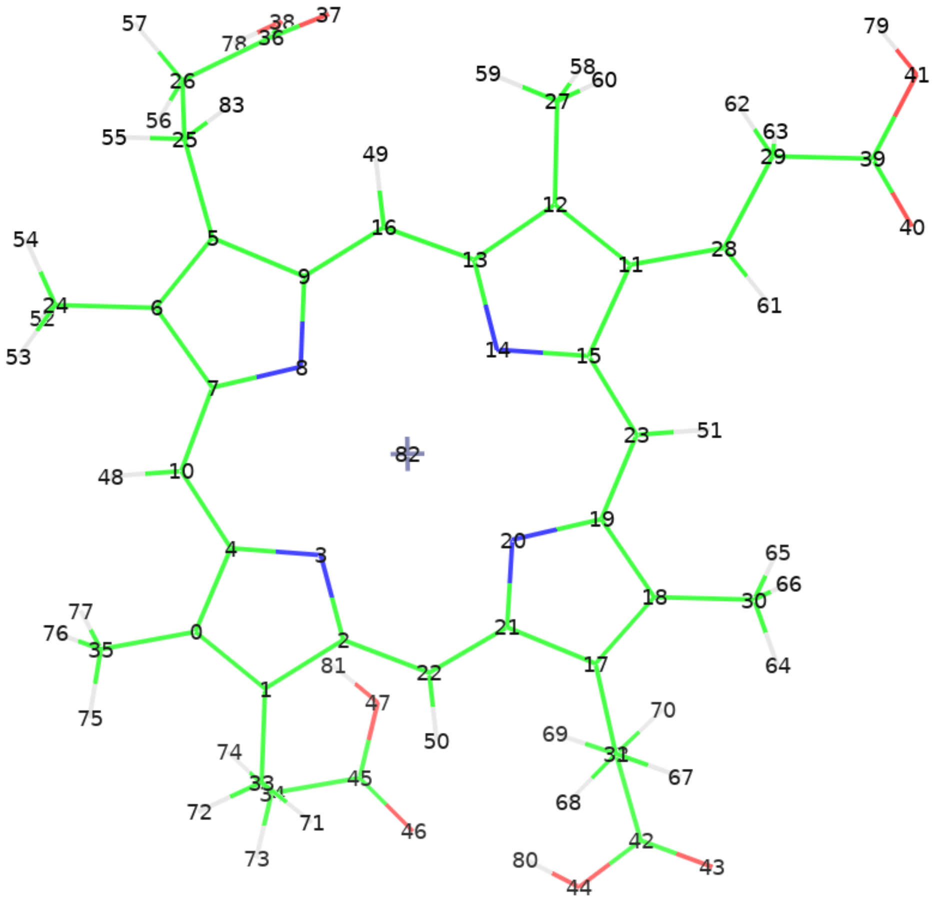

```
--------------------
ELECTRONIC G-MATRIX
--------------------

The g-matrix:
              2.0029357    0.0000355   -0.0000016
             -0.0000226    2.0030169    0.0001157
              0.0000216    0.0001442    2.0021360

 gel          2.0023193    2.0023193    2.0023193
 gRMC        -0.0001544   -0.0001544   -0.0001544
 gDSO(tot)    0.0002667    0.0002270    0.0002114
 gPSO(tot)   -0.0003145    0.0005433    0.0006600
             ----------   ----------   ----------
 g(tot)       2.0021171    2.0029352    2.0030363 iso=  2.0026962
 Delta-g     -0.0002022    0.0006159    0.0007170 iso=  0.0003769

Orientation:
  X          -0.0109439   -0.9969351   -0.0774642
  Y          -0.1428809    0.0782329   -0.9866431
  Z           0.9896794    0.0002704   -0.1432992
```

Euler rotation of hyperfine tensor to g-tensor

Atom	Alpha	Beta	Gamma	Ax	Ay	Az
		[degrees]			[MHz]	
0C	91.5	43.8	-93.2	6.56	-0.19	-0.42
1C	-90.8	15.0	57.3	-2.30	-2.15	-2.08
2C	90.6	43.2	-93.7	12.87	1.76	1.35
4C	-83.8	9.1	82.4	2.85	-0.89	-0.58
5C	-58.2	5.5	55.9	5.29	2.11	1.82
6C	87.1	45.0	-89.3	-6.37	-0.60	-0.69
7C	108.0	35.2	-88.6	-1.88	-1.72	-2.45
9C	-87.4	42.4	86.5	-17.55	-6.22	-5.35
10C	-88.8	36.6	87.1	4.68	-0.82	-0.61
11C	-86.3	1.3	87.8	-59.52	-25.60	-31.02
12C	75.8	0.9	-74.7	97.67	7.58	6.51
13C	89.1	27.4	-88.8	-36.08	-16.48	-18.16
15C	-85.6	43.8	85.4	2.99	-0.03	0.23
16C	-85.1	27.8	85.0	29.26	2.88	2.91
17C	-83.6	6.8	85.5	-4.97	-2.24	-2.06
18C	89.1	30.6	-89.2	7.06	0.27	0.45
19C	15.7	8.8	-16.3	-0.62	-0.97	-0.40
21C	-90.2	31.9	90.7	13.36	1.69	1.56
22C	98.7	15.1	-99.9	-12.59	-2.57	-3.18
23C	-116.5	1.5	116.8	4.98	-1.22	-0.57
24C	-93.8	31.8	93.4	0.83	0.93	0.89
25C	-153.3	31.8	159.1	-0.57	-0.29	-0.53
26C	146.7	56.1	-124.2	0.56	0.67	0.78
27C	146.8	26.8	-150.7	-12.90	-13.97	-12.73
28C	90.6	37.2	-88.7	135.23	5.85	7.64
29C	-88.1	28.0	96.3	-20.48	-21.52	-19.42
30C	98.8	34.4	-91.1	-1.17	-1.15	-0.97
31C	127.6	13.3	-100.6	0.24	0.29	0.41
32C	112.7	35.2	-85.8	-0.54	-0.39	-0.33
33C	-51.4	11.8	90.7	-0.14	-0.17	-0.01
34C	-16.3	38.8	29.6	0.37	0.33	0.50
35C	83.6	33.8	-82.2	-1.18	-1.02	-1.24
36C	-137.7	40.8	119.5	-0.08	0.18	-0.05
39C	39.7	33.4	-44.2	3.45	5.03	2.98
42C	36.1	18.3	-28.1	-0.06	-0.05	0.01
45C	58.0	26.7	-16.9	-0.05	-0.04	0.05
48H	85.6	21.8	-87.3	-2.01	-0.29	-2.83
49H	76.7	18.5	-77.6	-9.43	-10.98	-4.15
50H	109.1	14.1	-110.8	2.31	4.58	1.63
51H	93.8	13.6	-92.8	-3.54	-1.88	0.55
52H	-122.2	32.9	118.1	-2.89	-2.50	-2.77
53H	-48.6	35.6	50.0	-2.42	-1.99	-2.35
54H	80.5	13.0	-81.4	-0.26	0.07	-0.18
55H	-48.1	21.1	52.2	0.33	1.02	0.33

56H	-165.0	44.6	145.0	-0.17	0.44	-0.22
57H	-128.9	33.7	122.9	-0.24	0.31	-0.21
58H	-169.1	21.9	-172.1	44.85	46.33	50.58
59H	75.1	33.2	-85.2	0.61	1.02	5.74
60H	-5.3	17.6	1.2	29.14	31.27	35.07
61H	91.3	42.3	-89.5	-39.99	-61.23	-16.29
62H	-146.0	17.6	150.6	35.02	37.01	42.74
63H	-18.8	27.1	45.0	65.69	66.69	73.42
64H	89.8	36.2	-90.3	-0.24	-0.14	0.59
65H	86.6	31.8	-70.0	2.75	3.19	3.92
66H	94.7	31.5	-110.2	2.51	2.96	3.70
67H	104.3	13.1	-108.2	-0.63	-0.57	-0.10
68H	-173.8	7.1	-142.2	-0.49	-0.47	0.06
69H	-20.8	18.3	-3.5	-0.06	-0.04	0.44
70H	20.2	22.1	-31.7	-0.20	-0.10	0.36
71H	113.4	9.7	-148.5	-0.10	-0.07	0.47
72H	-119.5	18.6	111.6	-0.23	-0.17	0.28
73H	-53.3	15.1	47.5	-0.05	-0.04	0.22
74H	-20.6	28.9	33.0	-0.20	-0.16	0.21
75H	-119.3	44.1	90.2	-0.13	-0.15	0.52
76H	73.8	20.7	-61.3	2.43	3.20	2.56
77H	119.8	25.8	-140.1	3.20	3.96	3.29
78H	-149.7	39.7	119.2	-0.19	0.36	-0.17
79H	-76.0	38.9	101.5	-0.94	-0.74	1.48
80H	11.0	11.9	-3.9	-0.08	-0.06	0.14
81H	35.4	44.2	2.8	-0.21	-0.10	0.28
83H	-73.1	24.9	79.5	-0.22	1.18	-0.14
3N	-91.0	39.9	89.0	-2.02	-0.40	-0.33
8N	87.7	43.8	-90.2	6.42	0.26	0.11
14N	-84.4	11.4	84.7	10.47	-0.07	0.15
20N	-90.2	18.1	89.6	-1.83	-0.51	-0.43
82Zn	84.5	17.3	-89.8	-2.45	-2.32	-2.77

A_{iso} = 22.3 MHz

----

2 Supplementary Figures

Figure S1

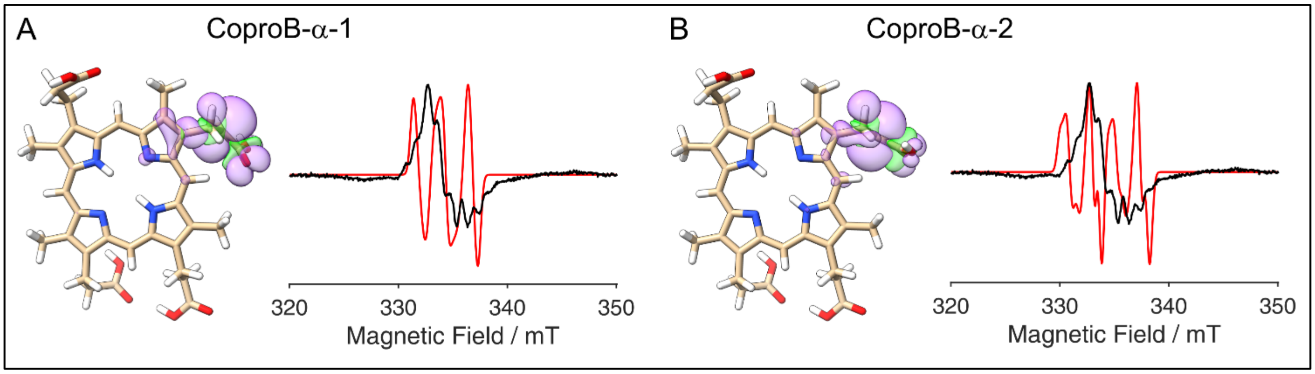

Figure S1. DFT calculated spin density plots and simulation of EPR spectra for hydrogen atom abstraction at the α-position of the ring B propionate side chain of Copro. Positive spin density is shown in purple, negative spin density is depicted in green, both at a cut off level $\sigma = \pm 4.5 \cdot 10^{-4}$. The experimental EPR spectrum is shown in black, the simulated spectra are depicted in red.

Figure S2

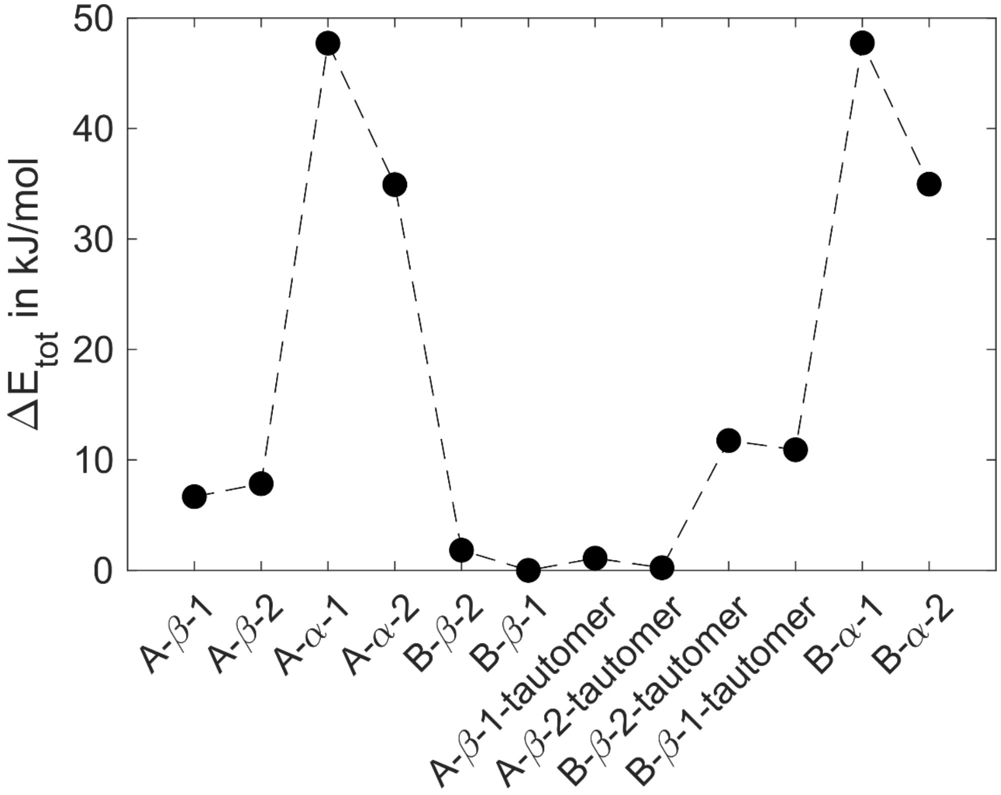

Figure S2. Relative total energy values $\boldsymbol{\Delta E}(\mathbf{Position})$ for the different Copro radical positions. The energy values $\boldsymbol{E}$ are the minimized energies after geometry optimization using a B3LYP functional and def2-TZVP basis set. Shown are relative values $\boldsymbol{\Delta E}(\mathbf{Position}) = \boldsymbol{E}(\mathbf{Position}) - \boldsymbol{E}_{\mathbf{min}}(\mathbf{Position})$ while $\boldsymbol{E}_{\mathbf{min}}(\mathbf{position})$ is the radical position with the lowest inner energy.

Figure S3

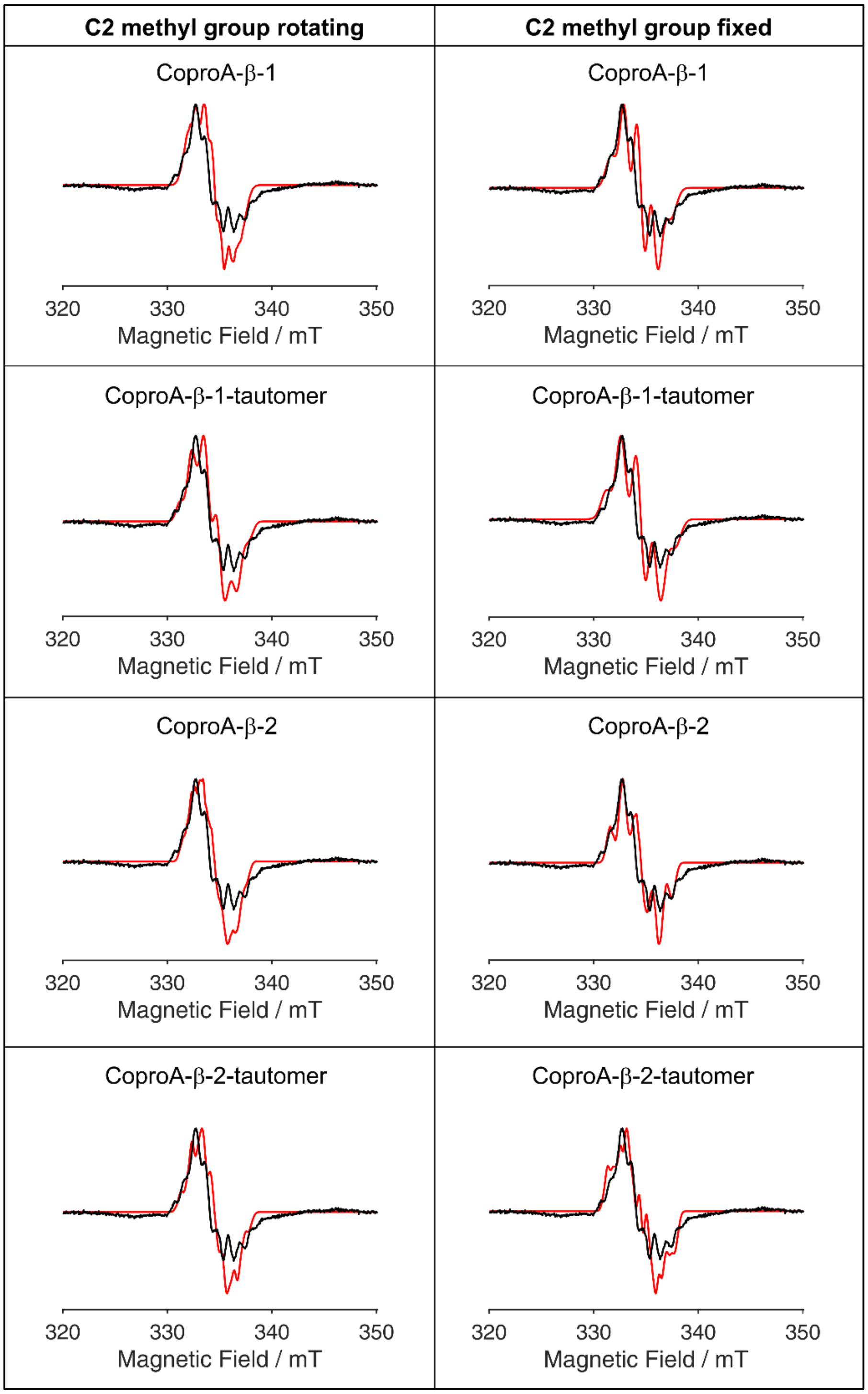

Figure S3. EPR simulations for the different CoproA radical positions. Left: EPR simulation of the DFT results assuming a free rotating methyl group (three equivalent isotropic proton hyperfine couplings). Right: EPR simulation of the DFT results assuming a fixed methyl group (three different anisotropic proton hyperfine couplings).

Figure S4

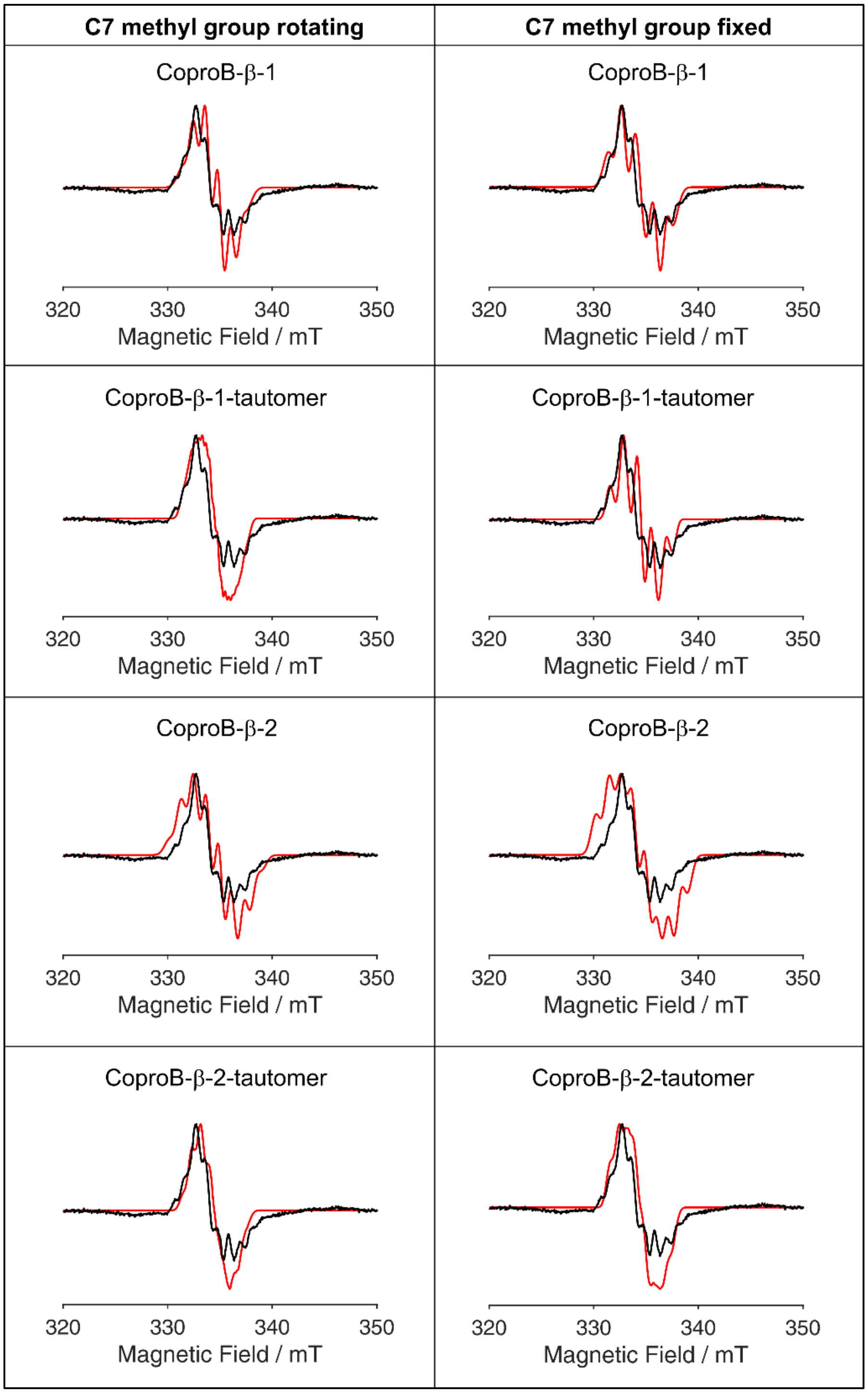

Figure S4. EPR simulations for the different CoproB radical positions. Left: EPR simulation of the DFT results assuming a free rotating methyl group (three equivalent isotropic proton hyperfine couplings). Right: EPR simulation of the DFT results assuming a fixed methyl group (three different anisotropic proton hyperfine couplings).
